# Canine Uterine Bacterial Infection Induces Upregulation of Proteolysis-Related Genes and Downregulation of Homeobox and Zinc Finger Factors

**DOI:** 10.1371/journal.pone.0008039

**Published:** 2009-11-26

**Authors:** Ragnvi Hagman, Elin Rönnberg, Gunnar Pejler

**Affiliations:** 1 Division of Small Animals, Department of Clinical Sciences, Swedish University of Agricultural Sciences, Uppsala, Sweden; 2 Department of Anatomy, Physiology and Biochemistry, Swedish University of Agricultural Sciences, Uppsala, Sweden; Columbia University, United States of America

## Abstract

**Background:**

Bacterial infection with the severe complication of sepsis is a frequent and serious condition, being a major cause of death worldwide. To cope with the plethora of occurring bacterial infections there is therefore an urgent need to identify molecular mechanisms operating during the host response, in order both to identify potential targets for therapeutic intervention and to identify biomarkers for disease. Here we addressed this issue by studying global gene expression in uteri from female dogs suffering from spontaneously occurring uterine bacterial infection.

**Principal Findings:**

The analysis showed that almost 800 genes were significantly (p<0.05) upregulated (>2-fold) in the uteri of diseased animals. Among these were numerous chemokine and cytokine genes, as well as genes associated with inflammatory cell extravasation, anti-bacterial action, the complement system and innate immune responses, as well as proteoglycan-associated genes. There was also a striking representation of genes associated with proteolysis. Robust upregulation of immunoglobulin components and genes involved in antigen presentation was also evident, indicating elaboration of a strong adaptive immune response. The bacterial infection was also associated with a significant downregulation of almost 700 genes, of which various homeobox and zinc finger transcription factors were highly represented.

**Conclusions/Significance:**

Together, these finding outline the molecular patterns involved in bacterial infection of the uterus. The study identified altered expression of numerous genes not previously implicated in bacterial disease, and several of these may be evaluated for potential as biomarkers of disease or as therapeutic targets. Importantly, since humans and dogs show genetic similarity and develop diseases that share many characteristics, the molecular events identified here are likely to reflect the corresponding situation in humans afflicted by similar disease.

## Introduction

Bacterial infection with the severe complication of a systemic inflammatory host response (sepsis) is a serious condition and the most common cause of death in intensive care units at hospitals, with a global incidence that remains rising [1], [2]. Despite this, our knowledge of the complex pathophysiology of sepsis is still is incomplete. Diagnosis of sepsis in critically ill patients is demanding because of unspecific clinical signs and imprecise traditional markers [3]. To improve current diagnostic methods for sepsis, it is therefore central to identify clinically useful biomarkers that may facilitate early and precise diagnosis [4], [5], [6]. Biomarkers may also constitute potential targets for novel treatments of bacterial infections, severe inflammation and sepsis [7].

Dogs are commonly used in experimental studies of sepsis as well as in safety assessment studies of pharmaceuticals since their inflammatory response is similar to humans [8], [9]. It is also important to stress that, following the sequencing of the canine genome [10], dogs are currently emerging as attractive models for studying the genetic background for diseases. Bacterial uterine infection (pyometra) is a common disease that develops in 25% of all intact female dogs [11]. The disease is characterized by mainly Gram-negative infection in combination with severe local and systemic inflammation [12]. Pyometra is lethal if left untreated and patients may develop endotoxemia, sepsis or septic shock [13], [14]. The most effective treatment is acute surgical removal of the uterus and ovaries (ovariohysterectomy).

Bacterial uterine infection in dogs has many similarities with severe bacterial infections in humans. For example, infection in both species is associated with induction of local and systemic inflammation, cytokine production, an acute phase reaction, endotoxemia and induction of subsequent sepsis. Therefore, an examination of disease mechanisms involved in pyometra may provide important insights to the mechanisms operating during human bacterial infection and sepsis [15], [16]. Here we used Affymetrix microarray technology to investigate the mechanisms involved in pyometra. We report that pyometra causes dramatic effects on the uterine gene expression pattern. A large number of genes associated with both innate and adaptive immune responses were upregulated, and there was also a striking upregulation of a wide array of proteases and protease inhibitors. Moreover, the uterine disease was clearly associated with downregulation of a panel of transcription factors of homeobox and zinc-finger type.

## Materials and Methods

### Animals

This research study was conducted according to national regulations (The Animal Welfare Act and Ordinance, The Swedish Ministry of Agriculture) and international guidelines (the European Convention and the European Commissions Directive 86/609/EEC on protection of animals used for experimental and other scientific purposes). The study was covered by an application approved by the Uppsala Animal Ethics Committee, Uppsala, Sweden. The dogs were privately-owned patients admitted and treated according to the routines at the University Animal Hospital, Swedish University of Agricultural Sciences, Uppsala, Sweden. Written owner consent was obtained before any dog was included.

Fifteen female dogs admitted to the University Animal Hospital, Swedish University of Agricultural Sciences, for diagnosis and subsequent surgical treatment (ovariohysterectomy, OHE) of pyometra were included in the study. The control group consisted of 6 healthy female dogs admitted for elective spay (OHE). Case history and physical examination data were noted by the veterinarian in charge on a specific form at admittance, and continued daily during the hospital stay.

### Blood- and Tissue Sampling

Blood samples for analysis of haematological and serum biochemical parameters were collected from all dogs before surgery from the distal cephalic vein into either non-additive, EDTA-containing or heparinized collection tubes (Becton-Dickinson, Stockholm, Sweden), chilled on ice and centrifuged. Plasma and serum were stored at −80°C until analysis. The removed uterus was cut open and a fibre swab (Culturette; Becton Dickinson AG) was used to sample the uterine contents for bacterial culturing. Tissue biopsies were snap-frozen in liquid nitrogen and stored at −80°C. The remaining uterine tissue and the ovaries were formaldehyde-fixated and used for histopathological examination.

### Blood Analyses

The following haematological and biochemical parameters were analysed, using routine methods: Packed cell volume (PCV, %), hemoglobin (Hb), total leukocyte count (WBC), differential count and morphological evaluation of blood smears, alanine aminotransferase (ALAT), alkaline phosphatase (AP), creatinine and urea. Serum levels of the acute phase proteins, C-reactive protein (CRP) and serum amyloid A (SAA), were analyzed in one batch with commercially available kits (Tridelta Phase™ range canine CRP ELISA assay, Tridelta™ Phase range SAA ELISA assay (Tridelta, Wicklow, Ireland)). Prostaglandin 15-keto-(13,14)-dihydro-F_2α_ metabolite (PGFM) levels were analyzed as previously described [17]. Statistically significant (p<0.05) differences in values between the infected and healthy dogs were determined using Student's *t*-tests (Table 1).

**Table 1:** Haematological-, biochemical-, acute phase protein- and inflammatory parameters in 15 female dogs with bacterial uterine infection (pyometra) and 6 healthy control dogs.

		Pyometra		Control	
	Reference value	Mean ± SD (Range)	n	Mean ± SD (Range)	n
Hemoglobin (g l^−1^)	132–199	121±30 (57–175)	15	140±21 (111–162)	5
EVF (10^12^ l^−1^)	0.38–0.57	0.34±0.08 (0.17–0.50)	15	0.39±0.06 (0.3–0.5)	5
WBC* (10^9^ l^−1^)	5.2–14.1	19.5±15.6 (2.8–59.4)	15	8.1±2.1 (6.3–11.0)	5
BN# (10^9^ l^−1^)	0–0.3	3.4±5.0 (0–13.7)	14	0.0±0.0 (0.0–0.0)	5
SN* (10^9^ l^−1^)	3.0–11.5	13.5±9.7 (2.0–35.6)	15	4.7±1.1 (3.7–6.3)	4
EoN (10^9^ l^−1^)	0.1–1.2	0.57±0.7 (0.0–2.4)	15	0.4±0.4 (0.2–1.1)	5
BaN (10^9^ l^−1^)	0–0.1	0.01±0.05 (0–0.2)	14	0.0±0.0 (0.0–0.0)	4
Lymphocytes* (10^9^ l^−1^)	1.4–4.8	1.4±0.9 (0.1–3.2)	15	2.2±0.8 (1.6 –3.6)	5
Monocytes* (10^9^ l^−1^)	0.2–1.4	1.6±1.9 (0.2–7.7)	15	0.4±0.2 (0.3–0.8)	5
Creatinine (µmol l^−1^)	40–130	106±177 (22–747)	15	76±14 (52–89)	6
ALAT (µkat l^−1^)	<1.2	0.5±0.3 (0.3–1.3)	14	0.6±0.2 (0.4–0.9)	6
Urea (mmol l^−1^)	2.5–8.5	7.4±14.0 (1.2–57.5)	15	5.2±1.3 (3.9–7.5)	6
AP* (µkat l^−1^)	<5.0	5.1±4.3 (0.8–18.2)	15	1.4±0.6 (0.8–2.3)	6
Albumin (g l^−1^)	31–43	35±40 (16–174)	14	30±2.2 (26–32)	6
Bile acids (g l^−1^)	<30	4.6±5.7 (0.0–20.4)	15	3.1±1.4 (1.2–4.6)	6
PGFM* (nmol l^−1^)		3695±3674 (464–13000)	15	477±117 (360–640)	6
SAA# (µg ml^−1^)		61.3±31 (<5–>80)	15	All<5	6
CRP* (µg ml^−1^)		38.5±17.5 (0.7–58.2)	15	1.3±1.2 (0.0–2.9)	6

WBC = total white blood cell count; BN = Band neutrophilic granulocytes; SN = segmented neutrophilic granulocytes; BaN = basophilic granulocytes; EoN = eosiniphilic granulocytes; ALAT = alanine aminotransferase; AP = alkaline phosphatase; PGFM = Prostaglandin F_2α_ metabolite, SAA = Serum amyloid A, CRP = C-reactive protein.

* Statistically significant difference between the pyometra group and control group (Student's t-test),

# difference not tested.

### Histopathological Examinations

Pyometra diagnosis was performed by gross and histopathological examinations of haematoxylin-eosin-stained sections of uteri and ovaries. Diagnostic criteria for pyometra with or without concomitant presence of macro- and/or microscopically visible cystic dilatation and epithelial hyperplasia of endometrial glands were uterine distension of a varying degree with macroscopically visible presence of opaque, yellowish to brownish exudates in the uterine lumen and microscopically visible purulent inflammatory changes in the endometrium and cystic glands.

### Microarray Expression Analysis and Data Analysis

Total RNA was prepared from cross-sections of frozen uterine tissue samples, using Nucleospin RNA II (Macherey-Nagel, Düren, Germany). Affymetrix gene chip microarray analysis was performed using the Canine Genome 2.0 Array, as described [18]. The raw data were normalized using the robust multi-array average (RMA) [19] background-adjusted, normalized and log-transformed summarized values. An empirical Bayes moderated *t-*test was applied to search for differentially expressed genes [20]. The *p*-values were adjusted to avoid the problem with multiple testing [21]. The Genesis software, version 1.7.1 (http://genome.tugraz.at/), was used to perform hierarchical clustering and to visualize differentially expressed genes [22]. All data is MIAME compliant and that the raw data has been deposited in a MIAME compliant database (the data is accessible via GEO using the accession no: GSE17878).

### Real-Time PCR

RNA was prepared using NucleoSpin® (Macherey-Nagel, Germany). First-Strand cDNA was synthesized with SuperScript™ II Reverse Transcriptase (Invitrogen, Inchinnan, UK) according to the manufacturers' instructions with 5 µl RNA and a final concentration of 7.5 ng/µl random hexamers (Invitrogen) in a total volume of 20 µl. The cDNA concentration was determined, and the cDNA was diluted to 800 ng/µl. Real-time PCR (qPCR) was performed on a ABI PRISM 7900 HT using iQ™ SYBR Green Supermix (BioRad, CA, USA) in a total volume of 10 µl, containing 80 ng cDNA and a final primer concentration of 100–300 nM. PCR cycling conditions included a 95°C heating step of 10 min at the beginning of every run. The samples were then cycled 40 times at 95°C for 30 s (denaturation), 58°C for 20 s (annealing) and 72°C for 20 s (extension). A melting curve from 60°C to 90°C was generated at the end of every run. Prior to experiments, the primer efficiency for each primer pair was determined with three different dilutions of the cDNA. The C_T_-values were plotted against Log concentrations of the dilutions and primer efficiency was calculated according to following formula: efficiency = 10^(−1/slope)^-1. The results were calculated by the comparative C_T_ method (User Bulletin #2: ABI PRISM 770 Sequence Detection System (P7N 4303859)), using Hypoxanthine guanine phosphoribosyl transferase (Hprt) as housekeeping gene. For primers used and primer efficiency, see Table S1.

## Results

### Clinical Data

Data from analyses of haematological, biochemical, acute phase protein and inflammatory parameters are displayed in Table 1. As shown in Fig. S1, bacterial infection of the uterus was associated with a profound inflammatory reaction, primarily involving infiltration of mononuclear cells, and there was a particular abundance of plasma cells. Neutrophils and eosinophils were rarely present. In contrast, tissue from healthy individuals lacked signs of inflammation or tissue remodeling. Clinical data for the 4 dogs selected for Affymetrix gene chip analysis (see below) are illustrated in Table 2. *Escherichia coli* were isolated from all 4 uteri selected for micro-array analysis, whereof 3 strains were haemolytic. By using clinical criteria for assessment of sepsis/systemic inflammatory response syndrome (SIRS) in dogs with the highest sensitivity (97%) and specificity (64%), three of the four selected dogs were determined SIRS-positive [23]. In one of the dogs (case 3), peritonitis with pus in the abdomen was apparent during surgery.

**Table 2:** Illustration of data from physical examinations, uterine bacterial cultures and postoperative hospitalisation in the 4 female dogs with bacterial uterine infection (pyometra) selected for micro array analysis.

Pyometra case	Uterine diameter (cm)	General condition	Temp(°C)	HR(/min)	RR(/min)	WBC(10^9^ l^−1^)	PBN(%)	SIRS	Animal hospital stay (days)
*Reference values*	*<1*	*Normal*	*38.0–39.0*	*<120*	*20–40*	5.2–14.1	*−*	*−*	*2*
11	3.5	Mildly depressed	39.5	120	46	10.6	12	+	4
12	4.0	Moderately depressed	39.3	100	20	32.7	42	+	2
13	5.0	Mildly depressed	39.6	140	20	26.8	35	+	4
16	2.0	Mildly depressed	38.3	100	20	29.3	0.1	−	2

Temp = Core temperature, HR = heart rate, RR = respiratory rate, WBC = total white blood cell count, PBN = percentage band neutrophils, SIRS = systemic inflammatory response syndrome.

### Affymetrix Gene Chip Analysis

In order to investigate the molecular events associated with the infection, total RNA extracted from uterine tissue of 4 diseased and 4 healthy control animals (blood parameters specified in Table S2) were subjected to Affymetrix gene chip microarray analysis. A hierarchic clustering of the samples revealed that the control and pyometra groups, respectively, clustered well together (Fig. S2). Using moderated t-statistics we analyzed for differentially expressed genes, using a 2-fold change and adj p<0.05 as thresholds for significance. This analysis revealed that almost 800 genes were significantly upregulated more than 2-fold. In Table 3, the 50 genes showing the largest extent of upregulation are listed and Table S3 displays the complete list of significantly upregulated genes. We also found that almost 700 genes were significantly (adj p<0.05) downregulated more than 2-fold. The 50 genes showing the largest extent of downregulation are depicted in Table 4, and Table S4 displays the complete list of downregulated genes. An examination of the significantly up- and downregulated genes revealed distinct gene families that were highly represented, and these were selected for further analysis and visualization.

**Table 3:** 50 genes showing the highest extent of significant (adj p<0.05) upregulation, in comparison with healthy controls, in uterus from animals diagnosed with uterine bacterial infection.

Gene Title	Gene symbol	ID	Fold change	adj. *p*. val
secretory leukocyte peptidase inhibitor	SLPI	CfaAffx.15167.1.S1_s_at	344,8	0,0006
interleukin 8	IL8	Cfa.3510.1.S2_at	242,9	0,0003
sphingomyelin phosphodiesterase, acid-like 3A	SMPDL3A	CfaAffx.2399.1.S1_s_at	194,4	0,0002
S100 calcium binding protein A9	S100A9	CfaAffx.26854.1.S1_at	160,7	0,0002
interferon induced transmembrane protein 2	IFITM2	CfaAffx.10684.1.S1_s_at	137,3	0,0002
similar to Ig lambda chain V region 4A precursor	LOC612066	CfaAffx.345.1.S1_s_at	129,5	0,0018
similar to Ig kappa chain C region, B allele	LOC475754	Cfa.12195.14.S1_s_at	124,8	0,0053
insulin-like growth factor binding protein 1	IGFBP1	CfaAffx.19068.1.S1_s_at	104,4	0,0004
similar to Ig heavy chain V-III region VH26 precursor	LOC490894	Cfa.4556.3.A1_a_at	94,5	0,0010
prostaglandin-endoperoxide synthase 2	PTGS2	Cfa.3449.1.S1_s_at	88,7	0,0017
serum amyloid A protein /// serum amyloid A1	SAA1	CfaAffx.14443.1.S1_at	65,9	0,0031
S100 calcium binding protein A8	S100A8	CfaAffx.26852.1.S1_at	56,6	0,0007
haptoglobin-related protein	HPR	Cfa.12245.2.A1_a_at	56,1	0,0025
similar to Immunoglobulin lambda-like polypeptide 1 precursor	LOC607558	Cfa.4465.2.S1_at	53,3	0,0010
serglycin	SRGN	Cfa.20785.1.S1_s_at	53,2	0,0002
similar to immunoglobulin iota chain preproprotein	LOC486411	Cfa.4465.2.S1_s_at	50,7	0,0010
similar to Ig lambda chain V-I region BL2 precursor	LOC607020	CfaAffx.265.1.S1_s_at	49,8	0,0162
complement component 6	C6	CfaAffx.28425.1.S1_s_at	48,5	0,0014
tissue factor pathway inhibitor 2	TFPI2	CfaAffx.3983.1.S1_at	48,1	0,0016
chemokine (C-X-C motif) ligand 14	CXCL14	CfaAffx.2498.1.S1_s_at	47,0	0,0010
chemokine (C-C motif) ligand 2	CCL2	Cfa.3851.1.S1_s_at	44,8	0,0008
matrix metallopeptidase 1 (interstitial collagenase)	MMP1	CfaAffx.23166.1.S1_s_at	44,7	0,0139
complement component 5a receptor 1	C5AR1	Cfa.3834.1.S1_at	43,4	0,0002
CD5 molecule-like	CD5L	Cfa.5955.1.S1_at	42,6	0,0068
Fc fragment of IgG, high affinity Ia, receptor (CD64)	FCGR1A	Cfa.173.1.A1_s_at	42,5	0,0004
macrophage receptor with collagenous structure	MARCO	Cfa.15713.1.A1_s_at	40,7	0,0002
similar to Small inducible cytokine A23 precursor (CCL23)	LOC480602	Cfa.12237.1.A1_at	40,7	0,0007
neutrophil cytosolic factor 2	NCF2	Cfa.2804.1.S1_at	39,7	0,0001
CD48 molecule	CD48	Cfa.14560.1.S1_at	38,9	0,0001
acyloxyacyl hydrolase (neutrophil)	AOAH	CfaAffx.5812.1.S1_at	37,0	0,0001
thrombospondin 4	THBS4	CfaAffx.14209.1.S1_s_at	31,2	0,0033
chemokine (C-X-C motif) ligand 10	CXCL10	Cfa.16590.1.S2_at	31,1	0,0264
similar to Small inducible cytokine A4 precursor (CCL4)	LOC480601	Cfa.5334.1.A1_s_at	30,8	0,0020
similar to Ig kappa chain V-II region RPMI 6410 precursor	LOC491492	CfaAffx.23613.1.S1_x_at	30,0	0,0114
matrix metallopeptidase 9 (gelatinase B)	MMP9	Cfa.3470.1.S1_s_at	29,4	0,0001
selectin L	SELL	CfaAffx.23335.1.S1_s_at	28,3	0,0004
similar to normal mucosa of esophagus specific 1	LOC478287	CfaAffx.25306.1.S1_x_at	28,2	0,0036
caspase 4, apoptosis-related cysteine peptidase	CASP4	Cfa.3589.1.S1_s_at	28,1	0,0002
similar to immunoglobulin J chain	LOC475166	CfaAffx.5291.1.S1_s_at	27,7	0,0022
secreted phosphoprotein 1	SPP1	Cfa.9240.1.S1_at	27,7	0,0038
CD163 molecule	CD163	Cfa.9647.1.A1_at	27,5	0,0010
chemokine (C-X-C motif) ligand 14	CXCL14	Cfa.21149.1.S1_at	27,5	0,0005
plasminogen activator inhibitor type 1	SERPINE2	CfaAffx.24902.1.S1_at	25,6	0,0004
peptidase inhibitor 3, skin-derived (SKALP)	PI3	CfaAffx.15155.1.S1_s_at	24,1	0,0044
similar to normal mucosa of esophagus specific 1	LOC478287	Cfa.11815.1.A1_at	23,8	0,0067
lymphocyte cytosolic protein 2	LCP2	Cfa.18362.1.S1_at	23,4	0,0002
regenerating islet-derived 3 gamma	REG3G	Cfa.16734.1.S1_s_at	23,2	0,0474
Fc fragment of IgG, low affinity IIIa, receptor (CD16a)	FCGR3A	Cfa.21258.1.S1_at	23,2	0,0010
membrane-spanning 4-domains, subfamily A, member 7	MS4A7	CfaAffx.16226.1.S1_at	22,7	0,0015

**Table 4:** 50 genes showing the highest extent of significant (adj p<0.05) downregulation, in comparison with healthy controls, in uterus from animals diagnosed with uterine bacterial infection.

Gene title	Gene symbol	ID	Fold change	adj. *p*. val
sulfotransferase	SULT1D1	Cfa.3502.1.S1_at	−30,6	0,018
EPH receptor A7	EPHA7	CfaAffx.6057.1.S1_s_at	−26,0	0,003
transcription factor CP2-like 1	TFCP2L1	Cfa.15666.1.A1_at	−15,2	0,002
synuclein, alpha interacting protein	SNCAIP	Cfa.13718.1.S1_s_at	−10,9	0,001
fasciculation and elongation protein zeta 1 (zygin I)	FEZ1	Cfa.18202.2.S1_a_at	−10,7	0,042
similar to phosphatidylethanolamine-binding protein 4	LOC608950	Cfa.17117.1.S1_at	−10,6	0,004
similar to esophageal cancer related gene 4 protein	LOC611190	CfaAffx.4062.1.S1_at	−10,5	0,001
trefoil factor 2	TFF2	Cfa.201.1.S1_at	−9,2	0,048
regulator of G-protein signaling 22	RGS22	CfaAffx.1736.1.S1_at	−8,9	0,002
epoxide hydrolase 2, cytoplasmic	EPHX2	Cfa.574.1.A1_at	−8,3	0,001
Norrie disease (pseudoglioma)	NDP	Cfa.4725.1.S1_at	−8,3	0,007
ankyrin 3, node of Ranvier (ankyrin G)	ANK3	CfaAffx.19834.1.S1_s_at	−8,1	0,003
rhophilin, Rho GTPase binding protein 2	RHPN2	Cfa.15.1.S1_at	−8,0	0,006
hydroxypyruvate isomerase homolog (E. coli)	HYI	CfaAffx.8731.1.S1_s_at	−7,9	0,004
ectonucleotide pyrophosphatase/phosphodiesterase 6	ENPP6	CfaAffx.12520.1.S1_at	−7,9	0,012
msh homeobox 2	MSX2	Cfa.3529.1.S1_at	−7,9	0,014
forkhead box A2	FOXA2	Cfa.13382.1.A1_at	−7,8	0,007
similar to Homeobox protein DLX-6	LOC482312	CfaAffx.4169.1.S1_at	−7,5	0,001
distal-less homeobox 5	DLX5	CfaAffx.4173.1.S1_at	−7,4	0,006
solute carrier family 30 (zinc transporter), member 2	SLC30A2	Cfa.5561.1.A1_at	−7,4	0,014
lymphoid enhancer-binding factor 1	LEF1	CfaAffx.17535.1.S1_s_at	−7,4	0,019
F-box and WD repeat domain containing 10	FBXW10	Cfa.11549.1.A1_at	−7,3	0,026
cholecystokinin	CCK	CfaAffx.8825.1.S1_s_at	−7,1	0,027
aldehyde dehydrogenase 1 family, member A1	ALDH1A1	Cfa.1715.1.S1_at	−7,1	0,018
epoxide hydrolase 2, cytoplasmic	EPHX2	CfaAffx.13394.1.S1_s_at	−7,0	0,001
glutamate-cysteine ligase, catalytic subunit	GCLC	CfaAffx.4309.1.S1_s_at	−7,0	0,014
phosphatidic acid phosphatase type 2 domain containing 1A	PPAPDC1A	Cfa.5652.1.A1_at	−6,9	0,023
similar to RIKEN cDNA 5133401N09	LOC484150	CfaAffx.3054.1.S1_at	−6,6	0,042
protein phosphatase 1, regulatory (inhibitor) subunit 1B	PPP1R1B	Cfa.20636.1.S1_at	−6,6	0,027
similar to Epithelial-cadherin precursor (E-cadherin)	LOC489647	CfaAffx.30291.1.S1_at	−6,6	0,009
DEP domain containing 7	DEPDC7	CfaAffx.11821.1.S1_at	−6,6	0,044
similar to EGFR-coamplified and overexpressed protein	LOC608562	Cfa.20305.1.S1_at	−6,6	0,009
betaine-homocysteine methyltransferase	BHMT	Cfa.11111.1.A1_at	−6,5	0,002
N-acetylated alpha-linked acidic dipeptidase 2	NAALAD2	CfaAffx.7397.1.S1_s_at	−6,4	0,038
similar to dachshund homolog 1 isoform a	LOC485489	CfaAffx.8384.1.S1_at	−6,4	0,016
carboxylesterase 2 (intestine, liver)	CES2	Cfa.19114.1.S1_at	−6,3	0,005
msh homeobox 1	MSX1	CfaAffx.24056.1.S1_at	−6,3	0,010
spermatid perinuclear RNA binding protein	STRBP	Cfa.19369.1.S1_at	−6,2	0,002
EF-hand domain (C-terminal) containing 2	EFHC2	Cfa.9584.1.A1_s_at	−6,1	0,001
cystathionase (cystathionine gamma-lyase)	CTH	Cfa.359.1.S1_at	−6,1	0,005
SH3 domain binding glutamic acid-rich protein like 2	SH3BGRL2	CfaAffx.5151.1.S1_s_at	−6,0	0,033
glutamate receptor interacting protein 1	GRIP1	Cfa.8622.1.A1_s_at	−5,9	0,006
CKLF-like MARVEL transmembrane domain containing 8	CMTM8	Cfa.8530.1.A1_s_at	−5,7	0,017
tumor-associated calcium signal transducer 1	TACSTD1	CfaAffx.4844.1.S1_at	−5,7	0,004
similar to myosin 18A isoform b	LOC475308	Cfa.1186.1.A1_s_at	−5,6	0,003
similar to ankyrin repeat domain 26	LOC610965	CfaAffx.12169.1.S1_at	−5,6	0,009
WNT inhibitory factor 1	WIF1	Cfa.4881.1.A1_at	−5,5	0,018
similar to Arg/Abl-interacting protein 2 isoform 1	LOC482906	CfaAffx.12091.1.S1_s_at	−5,4	0,012
RAS guanyl releasing protein 1 (calcium and DAG-regulated)	RASGRP1	Cfa.7809.1.A1_at	−5,4	0,040
similar to membrane-associated guanylate kinase-related (MAGI-3)	LOC479761	CfaAffx.24689.1.S1_at	−5,4	0,008

### Chemokines and Chemokine Receptors

**Figure 1:**
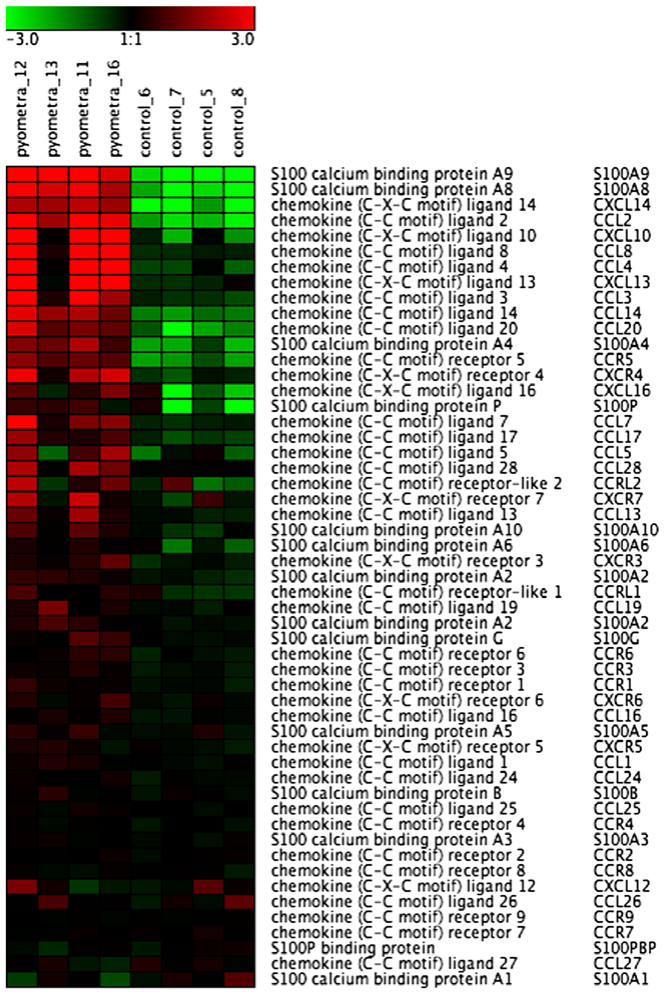
Heat map of differentially expressed chemokine, S100 protein and chemokine receptor genes in uteri from control animals and animals with pyometra (n = 4). The normalized 2 log intensity values were centered to the median value of each probe set and colored on a range of −3 to +3. Red denotes upregulated expression levels, and green denotes downregulated expression levels as compared with the median value.

A large number of chemokine genes were found among the most upregulated genes (Fig. 1; Tables 3 and S3). Not only did we find a number of CCL and CXCL chemokines, we also found a number of chemotactic proteins of the S100 family among the highly upregulated genes. In fact, S100A9 and S100A8 were among the genes showing the highest degree of upregulation of all genes. Out of the CCL and CXCL chemokines, CXCL14/BRAK and CCL2/MCP-1 showed the highest extent of upregulation. Among the chemokine receptors, CCR5 was upregulated to the largest extent.

### Cytokines

**Figure 2:**
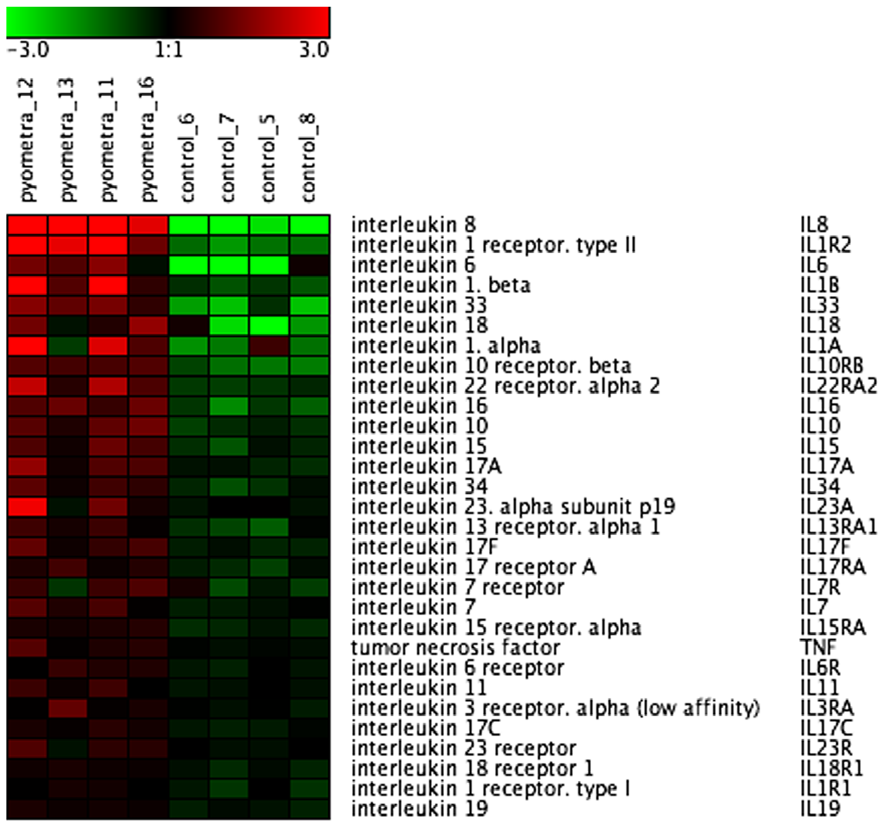
Heat map of differentially expressed cytokine receptor genes in uteri from control animals and animals with pyometra (n = 4). The normalized 2 log intensity values were centered to the median value of each probe set and colored on a range of −3 to +3. Red denotes upregulated expression levels, and green denotes downregulated expression levels as compared with the median value.

As displayed in Fig. 2 and Table 3/S3, a number of cytokines were upregulated in the uteri of diseased animals, with IL-8, IL-1 and IL-6 showing the highest extent of induction and there was also a high extent of IL-1 receptor upregulation. Also IL-33 and IL-18 were markedly upregulated. Somewhat unexpectedly, no significant upregulation of TNF or of any of the interferon family members was seen. In line with the upregulated cytokine expression, we noted a significant (3.3-fold) downregulation of suppressor of cytokine signaling 6 (SOCS6)(Table S4).

### Complement System

**Figure 3:**
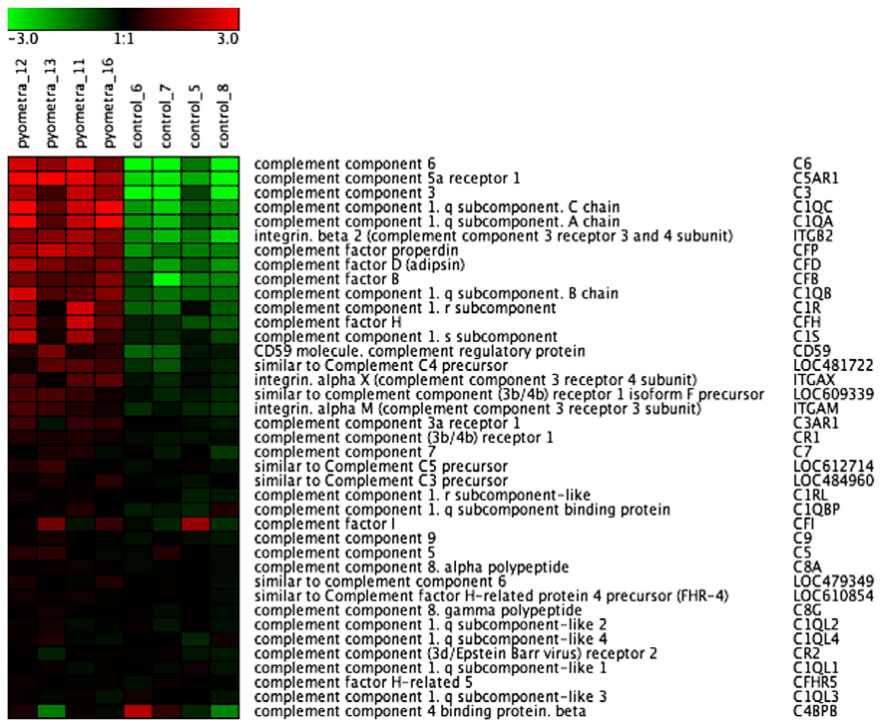
Heat map of differentially expressed complement-related genes in uteri from control animals and animals with pyometra (n = 4). The normalized 2 log intensity values were centered to the median value of each probe set and colored on a range of −3 to +3. Red denotes upregulated expression levels, and green denotes downregulated expression levels as compared with the median value.

The uterine infection caused a marked upregulation of a number of genes related to the complement system (Fig. 3; Tables 3/S3). Notably, both the classical and alternative pathways were represented, as shown by the upregulation of both C1 (classical pathway) as well as of Factors D, B and properdin (alternative pathway). Also C3 and C6, i.e. components that are shared by both pathways, were upregulated. Genes involved in the downregulation of the complement system were also upregulated, as shown by the strong upregulation of the C1 inhibitor: serpin peptidase inhibitor (clade G), member 1, and of Factor H. The C5a receptor was also dramatically upregulated.

### Proteases/Protease Inhibitors

**Figure 4:**
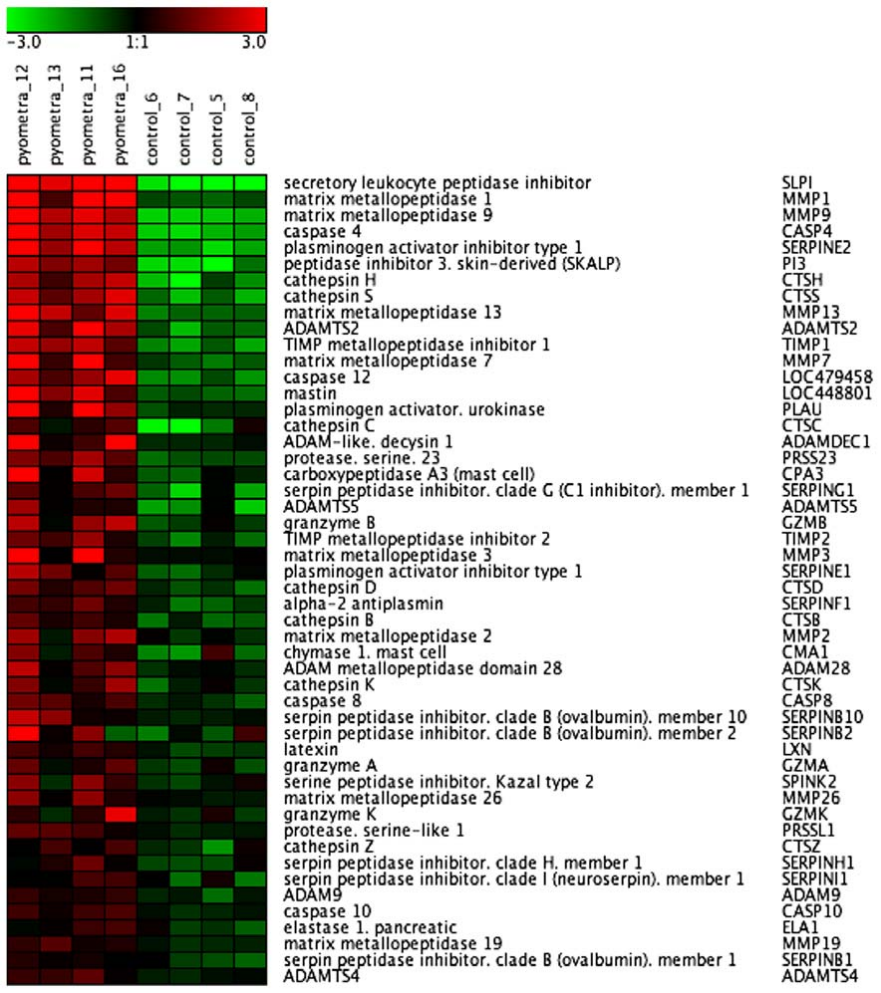
Heat map showing upregulated expression of protease and protease inhibitor genes in uteri from control animals and animals with pyometra (n = 4). The normalized 2 log intensity values for 50 genes (out of total 172 genes filtered as proteases/protease inhibitors) were centered to the median value of each probe set and colored on a range of -3 to +3. Red indicates upregulated expression, and green indicates downregulated expression as compared with the median value.

**Figure 5:**
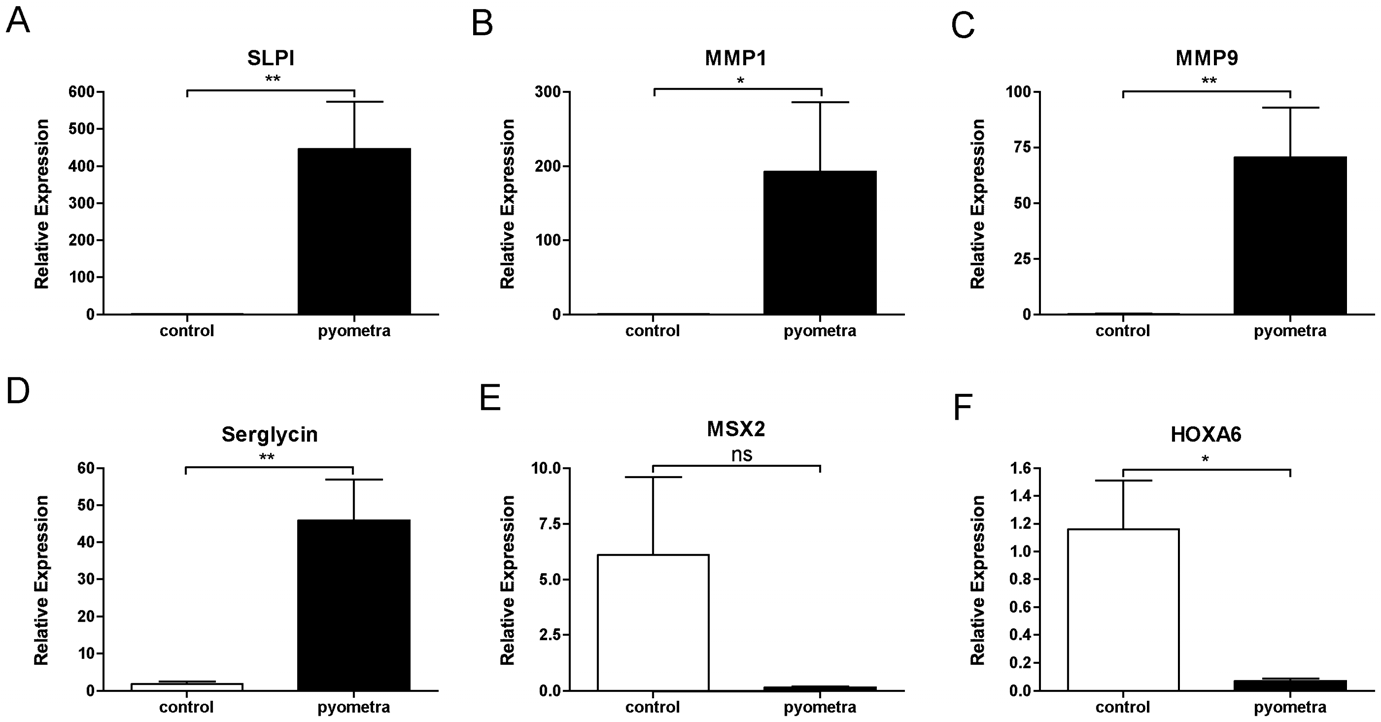
qPCR analysis confirming the up- or downregulation of selected genes in uterine tissue from dogs with pyometra. (A) SLPI; (B) MMP1; (C) MMP9; (D) SRGN; (E) MSX2; (F) HOXA6. All values are relative to one of the control samples. n = 7 (control); n = 5 (pyometra). p<0.05 *; p<0.01 **; p<0.001 ***; Student's t-test

An examination of the list of upregulated genes reveals a striking representation of genes related to proteolysis. Notably, the gene that showed the highest extent of upregulation among all genes was a protease inhibitor, secretory leukocyte peptidase inhibitor (SLPI; Table 3 and Fig. 4), and its profound upregulation in diseased animals was confirmed by qPCR analysis (Fig. 5A). SLPI is an inhibitor of neutrophil elastase and strong upregulation (∼24-fold) was also seen for an additional elastase inhibitor, SKALP/elafin (Table 3 and Fig. 4). Numerous matrix metalloprotease (MMP) members were upregulated, including the collagenases MMP-1 and MMP-13, as well as the gelatinase MMP-9 and MMP-7/matrilysin (Fig. 4 and Table 3/S3). The robust upregulation of MMP-1 and MMP-9 was confirmed by qPCR analysis (Fig. 5B, C). There was also a striking induction of TIMP-1, and -2, i.e. protease inhibitors with specificity for inhibiting proteases belonging to the MMP family (Fig. 4). Several proteases of the a disintegrin and metalloproteinase (ADAM) and ADAM with thrombospondin type 1-like motifs (ADAMTS) families were also markedly upregulated: ADAMTS2, ADAMTS5, ADAMDEC1 and ADAM28. Significant induction was also seen for various cysteine proteases, including caspase 4, -12 and -8, and several cysteine cathepsins, including cathepsin H, -S, -C and –B. Cathepsin D, an aspartic protease, was also significantly upregulated. Out of the large family of serine protease genes, the urokinase plasminogen activator gene showed the highest extent of upregulation and this was also reflected by a large extent of upregulation for the corresponding inhibitor, i.e. plasminogen activator inhibitor 1 (PAI-1). An upregulated expression of various mast cell proteases was also evident. In particular, a significant upregulation of mastin, a tetrameric, tryptase-like protease with gelatinase activity [24] was seen in diseased uteri.

### Proteoglycans/Anticoagulant Pathways

**Figure 6:**
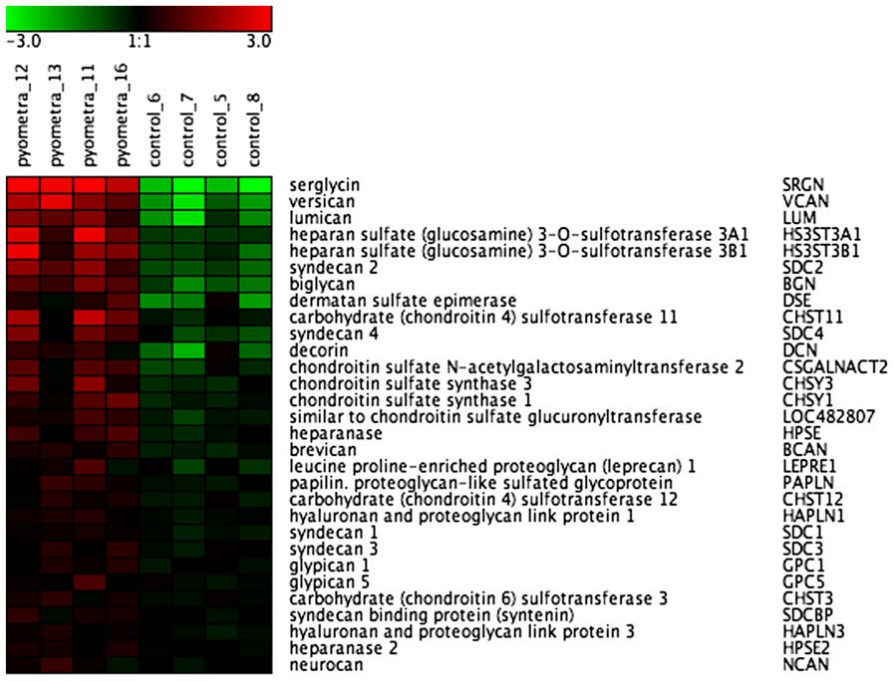
Heat map of differentially expressed proteoglycan genes and genes related to biosynthesis of the glycosaminoglycan component of proteoglycans (n = 4). The normalized 2 log intensity values for 30 genes (out of total 72 filtered genes) were centered to the median value of each probe set and colored on a range of -3 to +3. Red denotes upregulated expression levels, and green denotes downregulated expression levels as compared with the median value.

One of the genes that showed the highest extent of upregulation was the gene coding for the core protein of serglycin proteoglycan (SRGN), as shown both by Affymetrix gene chip analysis (Fig. 6) and by qPCR (Fig. 5D). Significant upregulation was also seen for versican, lumican, syndecan-2, biglycan and syndecan-4 (Fig. 6 and Tables 3/S3). The biological properties of proteoglycans are critically dependent on the nature of the glycosaminoglycan chains (heparan/chondroitin/dermatan/keratan sulfate or heparin) attached to the respective protein cores. Glycosaminoglycan chain synthesis is accomplished through the concerted action of a number of biosynthetic enzymes and we therefore analyzed the expression of the corresponding genes. As shown in Fig. 6, a strong induction of two heparan sulfate 3-O-sulfotransferase isoforms (HS3ST3A1 and HS3ST3B1) was evident in uterus from infected individuals and upregulation of the genes coding for dermatan sulfate epimerase, carbohydrate (chondroitin 4) sulfotransferase 11 and chondroitin sulfate N-acetylgalactosaminyltransferase 2 was also apparent (Fig. 6). Heparan sulfate 3-O-sulfotransferase catalyzes the incorporation of GlcNAc-3-O-sulfate into heparan sulfate and mast cell heparin, thereby conferring the respective glycosaminoglycan with potent anticoagulant activity [25]. Hence, the upregulated expression of the corresponding genes suggests that activation of anticoagulant mechanisms is a feature of bacterial infection of the uterus. In agreement with this notion, strong upregulation (48-fold) of another anticoagulant component, tissue factor pathway inhibitor 2, was also evident (Table 3). The role of anticoagulant pathways in the regulation of bacterial disease is also underscored by the established use of activated protein C, an anticoagulant protein, in treatment of sepsis [26].

### Prostaglandins

The uterine infection caused a massive (∼90-fold) upregulation of the prostaglandin-endoperoxide synthase 2/cyclooxygenase-2 gene (PTGS2)(Table 3). Further, strong upregulation of the prostaglandin D2 receptor (PTGDR) and prostaglandin E synthase (PTGES) genes was evident (Table 3/S3). The upregulation of prostaglandin-related genes is thus in accordance with the increased levels of prostaglandin F_2α_ metabolite in plasma from diseased dogs (Table 1). In contrast, genes related to leukotriene metabolism were not affected to any major extent.

### Immunoglobulins, Antigen Presentation

Various immunoglobulin (Ig)-related genes were highly represented (Table 3/S3), for example, Ig lambda chain V region 4A precursor, Ig kappa chain C region and Ig heavy chain V-III region VH26 precursor. In agreement with a strong upregulation of Ig genes, plasma cells were abundant in afflicted tissue (Fig. S1). Several genes associated with antigen presentation were also upregulated, including MHC class II DR alpha chain and MHC class II DLA DRB1 beta chain, CD48 (adhesion molecule involved in the immunological synapse) as well as cathepsin S, the latter being a cysteine protease implicated in antigen processing [27].

### Anti-Bacterial Genes

A natural consequence of the uterine infection would be an upregulated expression of various anti-bacterial proteins. Indeed, strong upregulated expression of lysozyme was apparent and there was also a marked upregulation of acyloxyacyl hydrolase, a lipase that partially deacylates bacterial lipopolysaccharide (LPS)(Table 3/S3). Strong induction was also seen for regenerating islet-derived 3 gamma (REG3G), an anti-bacterial compound that is expressed in an IL-22 and IL-23p19-dependent fashion [28], [29], and of bactericidal/permeability-increasing protein (BPI), the latter being an antimicrobial protein with LPS-neutralizing activity [30]. Moreover, clear upregulation of the anti-bacterial chemokines [31], CXCL14 [32] and CCL20 was evident. Somewhat unexpectedly, we did not see a significantly upregulated expression of any of the defensin family members.

### Acute-Phase Reactants

Pyometra and other bacterial infections are associated with elevated plasma levels of various acute phase reactants [33], [34]. Although the liver is considered to be the primary source for this group of proteins, it is apparent that several acute-phase reactants are also produced within the uterus, as shown by the dramatic (∼160-fold) upregulation of serum amyloid A (SAA)(Table 3). Notably, the strong upregulation of the SAA gene is consistent with the high levels of SAA found in serum from diseased animals (Table 1).

### Pattern Recognition Receptors

The onset of innate immunity reactions is typically triggered through the various pattern recognition receptors, including the toll-like receptors (TLRs). Out of these, we detected significant upregulation of, in particular, TLR2 (∼9-fold), but also TLR1 (∼5-fold) and, to a somewhat lesser extent, TLR4 (∼2.4-fold)(Table S3). There was also a profound upregulation of the mannose receptor (∼17-fold) and of several C type lectins: CLEC5A (∼14-fold), CLEC2D (13-fold), CLEC12A (7-fold), CLEC4D (4.5-fold) and CLEC4E (3.4-fold)(Table S3), but no significant upregulation of nucleotide-binding and oligomerization (NOD) 1 or NOD2.

### Cell Adhesion/Extravasation

Cell adhesion to the endothelium is a crucial step in the inflammatory process. Accordingly, several endothelial adhesion molecules were significantly upregulated in infected animals, in particular selectin L (28-fold), VCAM-1 (18-fold), selectin P (8.5-fold), ICAM-1 (7.6-fold) and selectin E (4-fold)(Table 3/S3). We also detected significantly downregulated expression of several genes involved in endothelial cell-cell contact formation, including genes coding for E-cadherin (6.6-fold), catenin (cadherin-associated protein), delta 2 (5.1-fold), cadherin 18, type 2 (4.7-fold), protocadherin 11 (3.7-fold) and occludin (3.3-dold). Clearly, the downregulated expression of the latter compounds is likely to enhance endothelial permeability and, hence, promote inflammatory cell infiltration into the uterus.

### Other Upregulated Genes

Among the remaining, upregulated genes, the gene coding for Sphingomyelin phosphodiesterase, acid-like 3A (SMPDL3A) showed the most dramatic extent of induction, being increased almost 200-fold in uteri from diseased animals (Table 3). Sphingomyelin phosphodiesterase is an enzyme that hydrolyzes sphingomyelin to ceramide and phosphorylcholine and, noteworthy, ceramide has been implicated in various pathophysiologial settings including apoptosis and infectious disease [35]. Other examples of profoundly (over 20-fold) upregulated genes included interferon induced transmembrane protein 2 (IFITM2), insulin-like growth factor binding protein 1 (IGFBP1), CD5 molecule-like (CD5L), lymphocyte cytosolic protein 2, CD48, thrombospondin 4, secreted phosphoprotein 1 (osteopontin), CD163, membrane-spanning 4-domains, subfamily A, member 7 (MS4A7) and Growth-associated protein-43 (GAP-43) (Tables 3/S3).

### Downregulated Genes/Homeobox and Zinc Finger Transcription Factors

**Figure 7:**
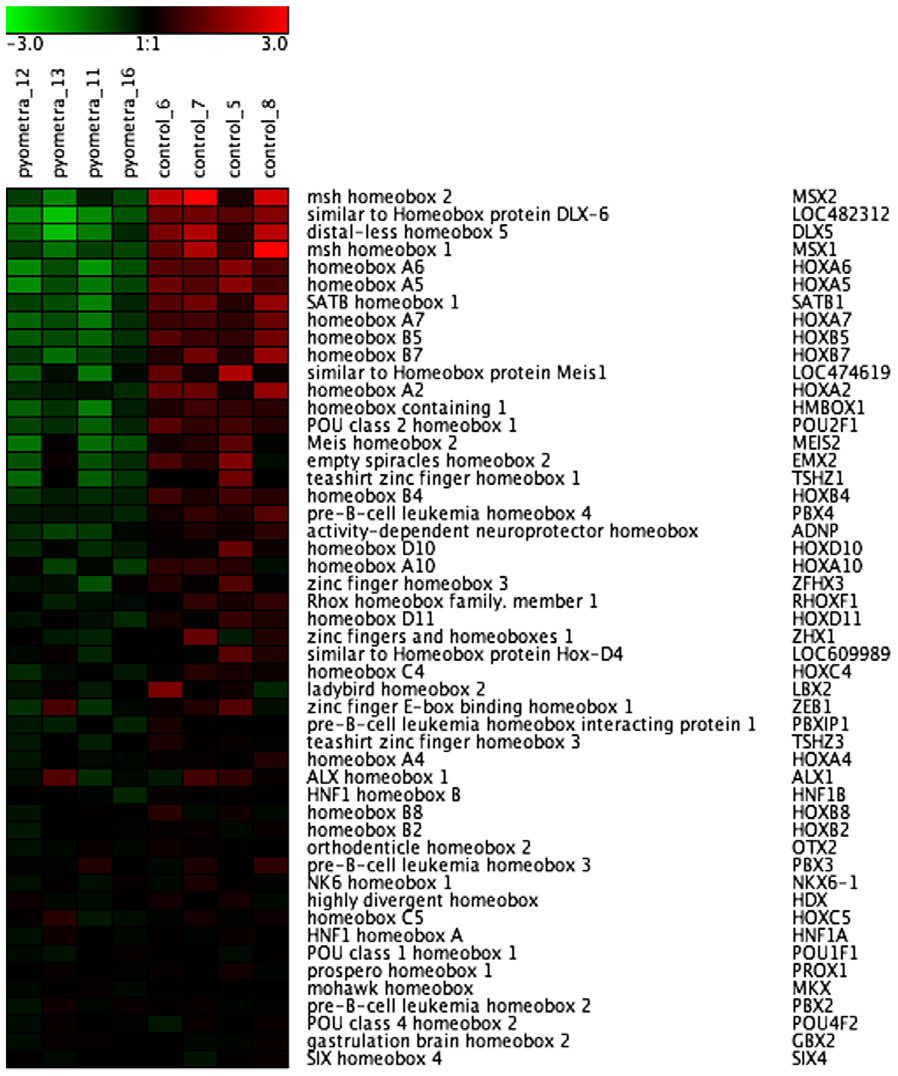
Heat map showing downregulated expression of homeobox genes in uteri from control animals and animals with pyometra (n = 4). The normalized 2 log intensity values for 50 homeobox genes (out of 176) were centered to the median value of each probe set and colored on a range of −3 to +3. Red denotes upregulated expression levels, and green denotes downregulated expression levels as compared with the median value.

**Figure 8:**
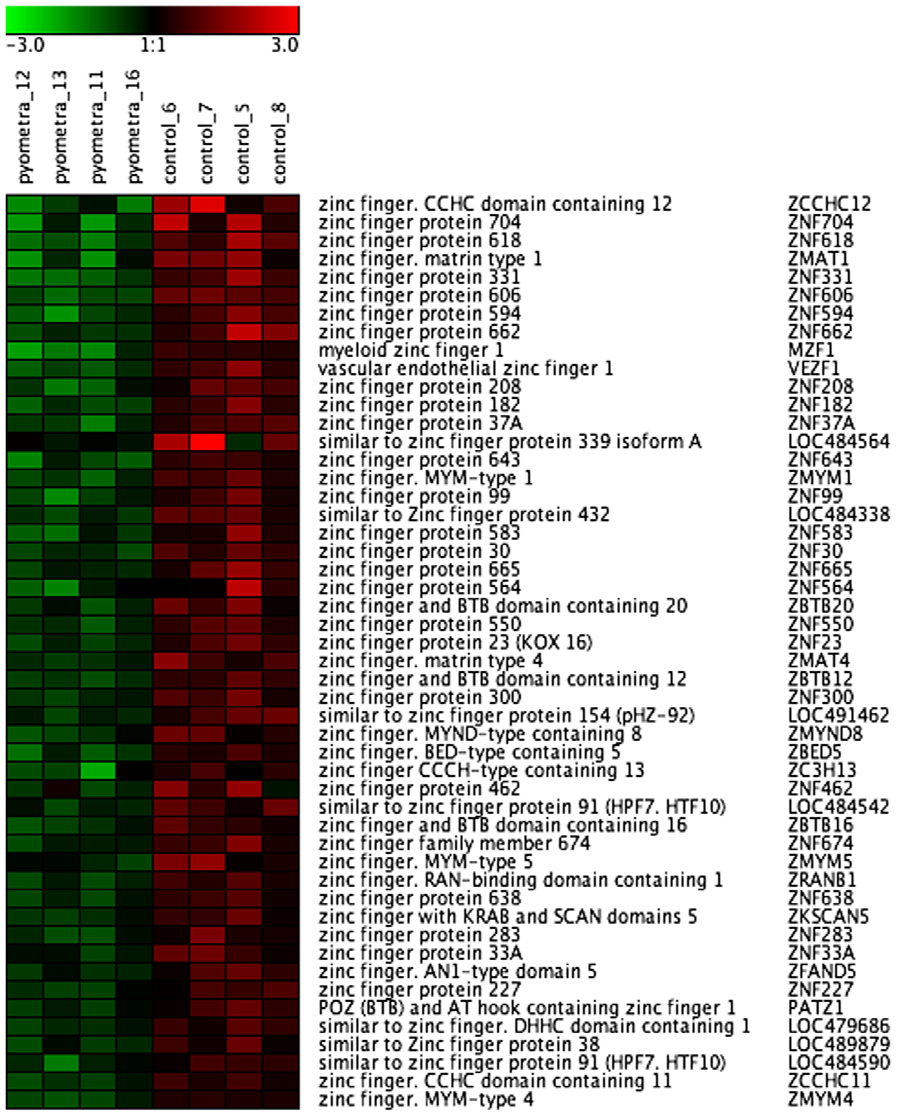
Heat map showing downregulated expression of zinc finger protein genes in uteri from control animals and animals with pyometra (n = 4). The normalized 2 log intensity values for 50 zinc finger genes (out of 489) were centered to the median value of each probe set and colored on a range of −3 to +3. Red denotes upregulated expression levels, and green denotes downregulated expression levels as compared with the median value.

The genes that were significantly downregulated in diseased animals included a number of genes with functions associated with signaling pathways (Table 4/S4). In particular, we note a striking presence of numerous members of the homeobox (Fig. 7) and zinc finger (Fig. 8) transcription factor families. The strong downregulation of homeobox genes in dogs afflicted with pyometra was also verified by qPCR analysis, as shown for MSX2 and HOXA6 (Fig. 5E, F). Notably, of all significantly downregulated (more than 2-fold) genes, 16 homeobox genes and 54 zinc finger genes were found. In contrast, homeobox and zinc finger proteins were only minimally represented (altogether 3 genes) among the significantly upregulated genes, being absent from the 100 most upregulated genes.

Other examples of markedly downregulated genes included SULT1D1 (∼30-fold; coding for sulfotransferase), EPHA7 (∼26-fold; coding for EPH receptor A7), TFCP2L1 (∼15-fold; coding for transcription factor CP2-like 1) and SNCAIP (∼11-fold; coding for synuclein, alpha interacting protein)(Table 4).

## Discussion

To the best of our knowledge, this is the first study in which the global gene expression pattern in the uterus is studied following a naturally occurring bacterial infection. Importantly, since a spontaneous disease rather than experimentally induced infection was used, the findings reflect a clinically relevant situation. Notably, previous attempts to extrapolate findings derived from experimental models of sepsis into a clinical setting have often encountered serious problems [36]. Another advantage of using the canine uterine disease as a model for sepsis is that the surgical treatment of the disease produces tissue samples readily available for studies of the local inflammatory response to bacterial infection. This is in contrast to corresponding human diseases, in which investigations are often limited to the use of blood sampling [37], [38], [39].

The pattern of upregulated genes clearly reflects an ongoing inflammatory response, as shown by the upregulated expression of several endothelial adhesion molecules, chemotactic proteins and cytokines. Among the cytokines, IL-6 and IL-1 were upregulated to the largest extent, and this was also reflected by a strong induction of the IL-1 receptor. Marked upregulation of IL-18 and IL-33 was also evident. Notably, IL-1, IL-18 and IL-33 are closely related cytokines, sharing structural and functional properties and, in addition, they are all activated by a caspase-1/inflammasome-dependent pathway [40]. We may thus suggest that activation of the inflammasome constitutes a major pathway for driving the inflammation seen in diseased animals. Given the wide implication of TNF during the host response to a plethora of pathogens, it was expected that pyometra would be associated with robust upregulation of TNF. However, the TNF gene was only marginally (∼1.5-fold) upregulated, not even reaching statistical significance. Most likely, this apparent paradox may reflect that the samples were taken from animals that had reached a late stage of disease, a stage where the initial rise in TNF levels during the early phase of infection may have declined. Following this, targeting of TNF may not constitute the most optimal regimen for therapy and, indeed, clinical trials for sepsis in which TNF was targeted have shown limited success (discussed in [36]).

As judged from the present study, a major feature of uterine infection is the upregulated expression of a large panel of proteases. In particular, there was a profound upregulation of various MMP members implicated in extracellular matrix (ECM) and chemokine [41] turnover, including collagenases (MMP-1, MMP-13), MMP-9 and MMP-7. We also note a robust upregulation of several caspases, a family of cysteine proteases strongly implicated in apoptotic processes, but also in a variety of other settings such as cancer and inflammation [42]. Out of the caspases, the most dramatic upregulation was seen for caspase-4, an “inflammatory” caspase that has been shown to promote nuclear factor kappa B (NF-κB) signaling and production of pro-inflammatory chemokines [43]. Also caspase-8 was upregulated. Caspase-8 is widely implicated in apoptosis but may also contribute to NF-κB activation through TLR4 [44]. Hence, its robust upregulation during uterine infection is in clear agreement with activation of the NF-κB pathway. Interestingly, strong upregulation was also seen for caspase-12, a protease that was recently shown to downregulate NF-κB signaling, thereby dampening the production of antibacterial peptides [45]. Hence, the uterine infection is associated with caspases capable of both promoting and dampening NF-κB-mediated effects on the immune system.

Uterine infection also caused a strong induction of several cysteine cathepsins. Traditionally, cystein cathepsins are mostly known as lysosomal enzymes involved in intracellular degradation processes. However, more recent data have revealed a much wider repertoire of functions, extending from roles in apoptosis to roles in cancer progression, wound healing and also in inflammatory disorders [46]. The present report thus indicates that cysteine cathepsin induction is a prominent feature of bacterial uterine infection. The uterine infection was also associated with a marked upregulation of several ADAM and ADAMTS metalloproteases, primarily ADAMTS2, ADAMTS5, ADAMDEC1 and ADAM28. Previous studies have implicated ADAMTS proteases mainly in ECM turnover and in regulation of angiogenesis [47] and the present report thus introduces the possibility that certain members of this protease family participate in bacterial disease. Members of the ADAM family have previously been implicated in a variety of disorders, such as asthma, cancer and autoimmune disease [48], but we are not aware of any previous in vivo evidence suggesting an involvement of any of the ADAM proteases in bacterial disease.

Since uncontrolled activation of proteolytic pathways may be harmful, it is critical that proteolytic activities are in balance with corresponding inhibitors. Indeed, a major finding in this study was the strong upregulation of various protease inhibitors. Strikingly, out of all upregulated genes, the gene coding for SLPI showed the highest extent of upregulation (∼340-fold). SLPI is an inhibitor of neutrophil elastase and its dramatic upregulation thus indicates that control of elastase activity is an important feature of the uterine infection. This notion is also supported by the strong upregulation of another elastase inhibitor, SKALP (24-fold). There was also a robust upregulation of MMP inhibitors, TIMP-1 and -2. In addition, a number of serine protease inhibitors of serpin type were induced. Out of these, plasminogen activator inhibitor 1 (PAI-1) showed the largest degree of upregulation and, notably, this was matched by a strong upregulation of the corresponding target, i.e. urokinase-type plasminogen activator.

SRGN, i.e. the gene coding for the core protein of serglycin proteoglycan, was one of the genes showing the largest extent of upregulation in diseased uteri. Serglycin has previously been shown to be critical for maintaining storage of secretory granule proteases in such cells [49] and the upregulated SRGN expression in diseased uteri may therefore be in line with the induction of proteolytic activities. Notably, mice lacking serglycin were previously shown to be more susceptible to *Klebsiella* infection than were wild type animals [50]. The present data thus support a prominent role for serglycin proteoglycan in host defense and also introduce the possibility to utilize serglycin as a biomarker for infection.

The massive downregulation of a number of homeobox and zinc finger genes during uterine infection is intriguing. Homeobox transcription factors have been widely implicated mainly in embryonal development and in cancer [18], [51] and the data presented here thus expand their repertoire of functions by implicating them in bacterial disease. Although we cannot with certainty explain why the homeobox genes are downregulated during disease, we may speculate that homeobox genes, during homeostatic conditions, have a role in suppressing pro-inflammatory pathways, and that downregulated expression of homeobox genes may unleash inflammatory cascades. In agreement with such a scenario, it has been shown that HOXA9 inhibits NF-κB-dependent activation of endothelium [52] and that mice with a reduced expression of Cdx2 are hypersensitive to dextran sodium sulfate-induced acute inflammation [53]. It is also of interest to note that a homeobox gene, TSHZ1 (teashirt), has previously been shown to inhibit caspase-4 gene expression [54]. Thus, the robust upregulation of caspase-4 in diseased animals (Table 3) is clearly compatible with the decreased expression of homeobox factors. There is also evidence suggesting that certain zinc finger proteins may have a homeostatic function by repressing pro-inflammatory responses, including suppression of the NF-κB pathway [55], [56]. Moreover, a recent study indicated that genes involved in zinc-related biology were downregulated during pediatric septic shock [39].

An obvious extension of the present work will be to evaluate whether any of the identified upregulated genes can be utilized either as biomarkers for disease or as therapeutic targets. Moreover, it will be important to address whether the respective identified gene product is specifically associated with uterine bacterial infection or if its upregulation is a general consequence of bacterial insult. We believe that the results presented here may provide a basis for numerous future investigations where the usefulness of the candidate genes/gene products identified are evaluated in both canine and corresponding human disease.

## Supporting Information

Table S1 Primers used in qPCR. Primers were designed for qPCR using the software Primer Express Version 1.0 O or Primer-BLAST, NCBI.
(0.06 MB DOC)

Table S2 Haematological-, biochemical-, acute phase protein- and inflammatory parameters in 4 female dogs with bacterial uterine infection (pyometra) and 4 healthy control dogs subjected to Affymetrix gene chip analysis.
(0.09 MB DOC)

Table S3 All genes showing significant (adj p<0.05) and more than 2-fold upregulation, in comparison with healthy controls, in uteri from animals diagnosed with uterine bacterial infection.
(1.23 MB DOC)

Table S4 All genes showing significant (adj p<0.05) and more than 2-fold downregulation, in comparison with healthy controls, in uteri from animals diagnosed with uterine bacterial infection
(0.88 MB DOC)

Figure S1 Histology of uteri from control (A) dogs and dogs diagnosed with uterine bacterial infection (pyometra; B). Note the extensive infiltration of mononuclear cells in infected animals, as well as the extensive loss of tissue organization.
(0.34 MB TIF)

Figure S2 Unsupervised clustering of gene expression in uteri from control animals and animal diagnosed with uterine bacterial infection (pyometra), data derived from the Affymetrix Canine Genome 2.0 Array.
(0.05 MB TIF)
