# Supplementary material for: Canine Uterine Bacterial Infection Induces Upregulation of Proteolysis-Related Genes and Downregulation of Homeobox and Zinc Finger Factors
Source: PLoS One. 2009 Nov 26;4(11):e8039. doi: 10.1371/journal.pone.0008039 (PMC2777310; doi:10.1371/journal.pone.0008039)
Supplement: Table S1 — Primers used in qPCR. Primers were designed for qPCR using the software Primer Express Version 1.0 O or Primer-BLAST, NCBI. (0.06 MB DOC) [file pone.0008039.s001.doc]

Table S1. **Primers used in qPCR**. Primers were designed for qPCR using the software *Primer Express* Version 1.0 O or Primer-BLAST, NCBI.

| Target | Sequence | Amplicon size (bp) | Efficiency^c^ |
| --- | --- | --- | --- |
| Hprt, *fw*^a^ | 5`-GCTGACCTGCTGGATTATATCAAAG -3` | 101 | 1,05 |
| Hprt, *rev*^b^ | 5`-TGGTCATTACAGTAGCTCTTCAGTCTG-3 |
| Serglycin, *fw* | 5`-ACTGAAGTGAACTGGTCACGATG-3` | 112 | 1,04 |
| Serglycin, *rev* | 5`-CTCTTCACAGGAGAACCTTGAACTG-3 25 |
| MSX2, *fw* | 5`- CGCCTCGGTCAAGTCGGAGA -3` | 102 | 1,09 |
| MSX2, *rev* | 5`- GTGCAGGTGGTGGGGCTCAT -3 |
| HOX6, *fw* | 5`- CCGGGCAAAGCCCTCCATGA -3` | 140 | 1,10 |
| HOX6, *rev* | 5`- TAGCGCGTGTAGGTCTGGCG -3 |
| MMP1, *fw* | 5`- TGGCCAAACCTGCCAAACGG -3` | 110 | 1,07 |
| MMP1, *rev* | 5`- AGCACATCCTGACCCTGAACAACC -3 |
| MMP9, *fw* | 5`- GCCGGCTCGGTGGAACAGAT -3` | 147 | 0,98 |
| MMP9, *rev* | 5`- GCCCACTTCGTCCACCTGGTT -3 |
| SLPI, *fw* | 5`- GCCGGAGTGACTGGCAGTGT -3` | 132 | 1,02 |
| SLPI, *rev* | 5`- GGCCATAGACCACCGGACACC -3 |
| ^a^fw, forward primer, ^b^rev, reverse primer, ^c^ Efficiency=10^(-1/slope)^-1 | | | |
