# Supplementary material for: Canine Uterine Bacterial Infection Induces Upregulation of Proteolysis-Related Genes and Downregulation of Homeobox and Zinc Finger Factors
Source: PLoS One. 2009 Nov 26;4(11):e8039. doi: 10.1371/journal.pone.0008039 (PMC2777310; doi:10.1371/journal.pone.0008039)
Supplement: Table S2 — Haematological-, biochemical-, acute phase protein- and inflammatory parameters in 4 female dogs with bacterial uterine infection (pyometra) and 4 healthy control dogs subjected to Affymetrix gene chip analysis. (0.09 MB DOC) [file pone.0008039.s002.doc]

Table S2. Haematological-, biochemical-, acute phase protein- and inflammatory parameters in 4 female dogs with bacterial uterine infection (pyometra) and 4 healthy control dogs subjected to Affymetrix gene chip analysis.

|  | **Pyometra** | | **Control** | |
| --- | --- | --- | --- | --- |
|  | Mean ± SD (Range) | n | Mean± SD  (Range) | n |
| Hemoglobin  (g l^-1^) | 147±18  (127-162) | 4 | 130±30  (110-175) | 4 |
| EVF  (10^12^ l^-1^) | 0.41±0.06  (0.34-0.47) | 4 | 0.37±0.09  (0.3-0.5) | 4 |
| WBC  (10^9^ l^-1^) | 8.2±2.4  (6.5-11.0) | 3 | 24.8±10  (10.6-32.7) | 4 |
| BN  (10^9^ l^-1^) | 0±0  (0-0) | 3 | 6.7±5.8  (1.3-13.7) | 4 |
| Neutrophils  (10^9^ l^-1^) | 4.8±1.3  (3.7-6.3) | 3 | 13.9±5.7  (7.3-21.1) | 4 |
| Eosinophils  (10^9^ l^-1^) | 0.57±0.5  (0.3-1.1) | 3 | 0.05±0.1  (0.0-0.2) | 4 |
| Basophils  (10^9^ l^-1^) | 0±0  (0-0) | 3 | 0±0  (0-0) | 4 |
| Lymphocytes  (10^9^ l^-1^) | 2.4±1.1  (1.6-3.6) | 3 | 1.9±1.1  (0.5 -3.2) | 4 |
| Monocytes  (10^9^ l^-1^) | 0.5±0.3  (0.3-0.8) | 3 | 2.2±0.8  (1.3-3.2) | 4 |
| Creatinine  (μmol l^-1^) | 77±17  (52-89) | 3 | 60±12  (44-72) | 4 |
| ALAT  (μkat l^-1^) | 0.6±0.1  (0.4-0.7) | 3 | 0.5±0.3  (0.3-0.9) | 4 |
| Urea  (mmol l^-1^) | 5.5±1.4  (4.4-7.5) | 3 | 3.9±1.8  (2.8-6.5) | 4 |
| Albumin  (g l^-1^) | 30±1  (29-31) | 3 | 22±4  (16-25) | 4 |
| Bile acids  (g l^-1^) | 3.1±1.0  (2.0-4.3) | 3 | 6.6±9.3  (0.6-20.4) | 4 |
| PGFM  (nmol l^-1^) | 4.62±5.64  (0.78-13.0) | 4 | 0.45±0.10  (0.36-0.60) | 4 |
| SAA  (μg ml^-1^) | 76- >80 | 4 | <5 | 4 |
| CRP  (μg ml^-1^) | 38.9±11.6  (25.2-48.8) | 4 | 2.02±3.4  (0.2-7.1) | 4 |

WBC=total white blood cell count; BN=Band neutrophilic granulocytes; SN=segmented neutrophilic granulocytes; BaN=basophilic granulocytes; EoN=eosinophilic granulocytes; ALAT=alanine aminotransferase; AP=alkaline phosphatase; PGFM= Prostaglandin F_2α_ metabolite, SAA= Serum amyloid A, CRP=C-reactive protein.
