# Supplementary material for: Canine Uterine Bacterial Infection Induces Upregulation of Proteolysis-Related Genes and Downregulation of Homeobox and Zinc Finger Factors
Source: PLoS One. 2009 Nov 26;4(11):e8039. doi: 10.1371/journal.pone.0008039 (PMC2777310; doi:10.1371/journal.pone.0008039)
Supplement: Table S3 — All genes showing significant (adj p<0.05) and more than 2-fold upregulation, in comparison with healthy controls, in uteri from animals diagnosed with uterine bacterial infection. (1.23 MB DOC) [file pone.0008039.s003.doc]

Table S3. All genes showing significant (adj p<0.05) and more than 2-fold upregulation, in comparison with healthy controls, in uteri from animals diagnosed with uterine bacterial infection

| **Gene Title** | **Gene**  **Symbol** | **ID** | **Fold**  **change** | **adj.P.**  **Val** |
| --- | --- | --- | --- | --- |
| secretory leukocyte peptidase inhibitor | SLPI | CfaAffx.15167.1.S1_s_at | 344,8 | 0,0006 |
| interleukin 8 | IL8 | Cfa.3510.1.S2_at | 242,9 | 0,0003 |
| sphingomyelin phosphodiesterase, acid-like 3A | SMPDL3A | CfaAffx.2399.1.S1_s_at | 194,4 | 0,0002 |
| S100 calcium binding protein A9 | S100A9 | CfaAffx.26854.1.S1_at | 160,7 | 0,0002 |
| interferon induced transmembrane protein 2 | IFITM2 | CfaAffx.10684.1.S1_s_at | 137,3 | 0,0002 |
| similar to Ig lambda chain V region 4A precursor | LOC612066 | CfaAffx.345.1.S1_s_at | 129,5 | 0,0018 |
| similar to Ig kappa chain C region, B allele | LOC475754 | Cfa.12195.14.S1_s_at | 124,8 | 0,0053 |
| insulin-like growth factor binding protein 1 | IGFBP1 | CfaAffx.19068.1.S1_s_at | 104,4 | 0,0004 |
| similar to Ig heavy chain V-III region VH26 precursor | LOC490894 | Cfa.4556.3.A1_a_at | 94,5 | 0,0010 |
| prostaglandin-endoperoxide synthase 2 | PTGS2 | Cfa.3449.1.S1_s_at | 88,7 | 0,0017 |
| serum amyloid A protein /// serum amyloid A1 | SAA1 | CfaAffx.14443.1.S1_at | 65,9 | 0,0031 |
| S100 calcium binding protein A8 | S100A8 | CfaAffx.26852.1.S1_at | 56,6 | 0,0007 |
| haptoglobin-related protein | HPR | Cfa.12245.2.A1_a_at | 56,1 | 0,0025 |
| similar to Immunoglobulin lambda-like polypeptide 1 precursor | LOC607558 | Cfa.4465.2.S1_at | 53,3 | 0,0010 |
| serglycin | SRGN | Cfa.20785.1.S1_s_at | 53,2 | 0,0002 |
| similar to immunoglobulin iota chain preproprotein | LOC486411 | Cfa.4465.2.S1_s_at | 50,7 | 0,0010 |
| similar to Ig lambda chain V-I region BL2 precursor | LOC607020 | CfaAffx.265.1.S1_s_at | 49,8 | 0,0162 |
| complement component 6 | C6 | CfaAffx.28425.1.S1_s_at | 48,5 | 0,0014 |
| tissue factor pathway inhibitor 2 | TFPI2 | CfaAffx.3983.1.S1_at | 48,1 | 0,0016 |
| chemokine (C-X-C motif) ligand 14 | CXCL14 | CfaAffx.2498.1.S1_s_at | 47,0 | 0,0010 |
| chemokine (C-C motif) ligand 2 | CCL2 | Cfa.3851.1.S1_s_at | 44,8 | 0,0008 |
| matrix metallopeptidase 1 (interstitial collagenase) | MMP1 | CfaAffx.23166.1.S1_s_at | 44,7 | 0,0139 |
| complement component 5a receptor 1 | C5AR1 | Cfa.3834.1.S1_at | 43,4 | 0,0002 |
| CD5 molecule-like | CD5L | Cfa.5955.1.S1_at | 42,6 | 0,0068 |
| Fc fragment of IgG, high affinity Ia, receptor (CD64) | FCGR1A | Cfa.173.1.A1_s_at | 42,5 | 0,0004 |
| macrophage receptor with collagenous structure | MARCO | Cfa.15713.1.A1_s_at | 40,7 | 0,0002 |
| similar to Small inducible cytokine A23 precursor (CCL23) | LOC480602 | Cfa.12237.1.A1_at | 40,7 | 0,0007 |
| neutrophil cytosolic factor 2 | NCF2 | Cfa.2804.1.S1_at | 39,7 | 0,0001 |
| CD48 molecule | CD48 | Cfa.14560.1.S1_at | 38,9 | 0,0001 |
| acyloxyacyl hydrolase (neutrophil) | AOAH | CfaAffx.5812.1.S1_at | 37,0 | 0,0001 |
| thrombospondin 4 | THBS4 | CfaAffx.14209.1.S1_s_at | 31,2 | 0,0033 |
| chemokine (C-X-C motif) ligand 10 | CXCL10 | Cfa.16590.1.S2_at | 31,1 | 0,0264 |
| similar to Small inducible cytokine A4 precursor (CCL4) | LOC480601 | Cfa.5334.1.A1_s_at | 30,8 | 0,0020 |
| similar to Ig kappa chain V-II region RPMI 6410 precursor | LOC491492 | CfaAffx.23613.1.S1_x_at | 30,0 | 0,0114 |
| matrix metallopeptidase 9 (gelatinase B) | MMP9 | Cfa.3470.1.S1_s_at | 29,4 | 0,0001 |
| selectin L | SELL | CfaAffx.23335.1.S1_s_at | 28,3 | 0,0004 |
| similar to normal mucosa of esophagus specific 1 | LOC478287 | CfaAffx.25306.1.S1_x_at | 28,2 | 0,0036 |
| caspase 4, apoptosis-related cysteine peptidase | CASP4 | Cfa.3589.1.S1_s_at | 28,1 | 0,0002 |
| similar to immunoglobulin J chain | LOC475166 | CfaAffx.5291.1.S1_s_at | 27,7 | 0,0022 |
| secreted phosphoprotein 1 | SPP1 | Cfa.9240.1.S1_at | 27,7 | 0,0038 |
| CD163 molecule | CD163 | Cfa.9647.1.A1_at | 27,5 | 0,0010 |
| chemokine (C-X-C motif) ligand 14 | CXCL14 | Cfa.21149.1.S1_at | 27,5 | 0,0005 |
| plasminogen activator inhibitor type 1 | SERPINE2 | CfaAffx.24902.1.S1_at | 25,6 | 0,0004 |
| peptidase inhibitor 3, skin-derived (SKALP) | PI3 | CfaAffx.15155.1.S1_s_at | 24,1 | 0,0044 |
| similar to normal mucosa of esophagus specific 1 | LOC478287 | Cfa.11815.1.A1_at | 23,8 | 0,0067 |
| lymphocyte cytosolic protein 2 | LCP2 | Cfa.18362.1.S1_at | 23,4 | 0,0002 |
| regenerating islet-derived 3 gamma | REG3G | Cfa.16734.1.S1_s_at | 23,2 | 0,0474 |
| Fc fragment of IgG, low affinity IIIa, receptor (CD16a) | FCGR3A | Cfa.21258.1.S1_at | 23,2 | 0,0010 |
| membrane-spanning 4-domains, subfamily A, member 7 | MS4A7 | CfaAffx.16226.1.S1_at | 22,7 | 0,0015 |
| ATPase, H+/K+ transporting, nongastric, alpha polypeptide | ATP12A | Cfa.3683.1.A1_at | 21,7 | 0,0215 |
| branched chain aminotransferase 1, cytosolic | BCAT1 | CfaAffx.17875.1.S1_at | 20,6 | 0,0003 |
| similar to embigin homolog | LOC479340 | CfaAffx.28283.1.S1_at | 20,2 | 0,0009 |
| growth associated protein 43 | GAP43 | Cfa.9841.1.S1_at | 20,2 | 0,0015 |
| complement component 1, q subcomponent, C chain | C1QC | Cfa.10921.1.S1_at | 20,1 | 0,0012 |
| podoplanin | PDPN | Cfa.3749.1.S1_at | 20,0 | 0,0028 |
| interleukin 1 receptor, type II | IL1R2 | Cfa.5221.1.A1_s_at | 19,7 | 0,0024 |
| TYRO protein tyrosine kinase binding protein | TYROBP | Cfa.12235.1.A1_s_at | 19,7 | 0,0003 |
| pro-platelet basic protein (chemokine (C-X-C motif) ligand 7) | PPBP | CfaAffx.5498.1.S1_at | 18,7 | 0,0009 |
| bactericidal/permeability-increasing protein | BPI | CfaAffx.14056.1.S1_s_at | 18,6 | 0,0007 |
| similar to egf-like module containing, mucin-like, hormone receptor-like sequence 2 isoform d | LOC484897 | Cfa.17456.1.S1_at | 18,4 | 0,0203 |
| similar to Ig lambda chain V-III region LOI | LOC486394 | CfaAffx.22878.1.S1_at | 18,3 | 0,0113 |
| vascular cell adhesion molecule 1 | VCAM1 | CfaAffx.30622.1.S1_s_at | 18,3 | 0,0138 |
| complement component 3 | C3 | Cfa.13267.1.A1_s_at | 18,0 | 0,0025 |
| interleukin 6 (interferon, beta 2) | IL6 | Cfa.3528.1.S1_s_at | 17,8 | 0,0324 |
| chemokine (C-C motif) ligand 8 | CCL8 | CfaAffx.28084.1.S1_s_at | 17,7 | 0,0344 |
| uridine phosphorylase 1 | UPP1 | CfaAffx.22028.1.S1_at | 17,5 | 0,0002 |
| complement component 1, q subcomponent, A chain | C1QA | CfaAffx.22561.1.S1_s_at | 17,1 | 0,0021 |
| similar to mannose receptor, C type 1-like 1 | LOC487114 | CfaAffx.7698.1.S1_at | 16,9 | 0,0007 |
| capping protein (actin filament), gelsolin-like | CAPG | CfaAffx.12503.1.S1_s_at | 16,8 | 0,0015 |
| lysozyme (renal amyloidosis) | LYZ | CfaAffx.1598.1.S1_s_at | 16,5 | 0,0006 |
| cytidine deaminase | CDA | Cfa.14519.1.A1_at | 16,4 | 0,0002 |
| SAM domain, SH3 domain and nuclear localization signals 1 | SAMSN1 | CfaAffx.12896.1.S1_s_at | 16,2 | 0,0015 |
| allograft inflammatory factor 1 | AIF1 | Cfa.14366.1.S1_s_at | 16,2 | 0,0002 |
| chemokine (C-C motif) ligand 4 | CCL4 | Cfa.15795.1.A1_s_at | 16,0 | 0,0312 |
| coagulation factor XIII, A1 polypeptide | F13A1 | Cfa.2335.1.A1_at | 15,9 | 0,0146 |
| ecotropic viral integration site 2B | EVI2B | CfaAffx.28458.1.S1_at | 15,7 | 0,0002 |
| cytochrome b-245, beta polypeptide (chronic granulomatous disease) | CYBB | CfaAffx.21514.1.S1_s_at | 15,6 | 0,0003 |
| indoleamine-pyrrole 2,3 dioxygenase | INDO | Cfa.19648.1.S1_at | 15,6 | 0,0066 |
| Fc receptor-like A | FCRLA | CfaAffx.20171.1.S1_s_at | 15,0 | 0,0006 |
| prostaglandin D2 receptor (DP) | PTGDR | CfaAffx.22642.1.S1_at | 14,9 | 0,0056 |
| coronin, actin binding protein, 1A | CORO1A | Cfa.17115.1.S1_s_at | 14,8 | 0,0004 |
| Bruton agammaglobulinemia tyrosine kinase | BTK | Cfa.16454.1.S1_at | 14,7 | 0,0001 |
| nicotinamide N-methyltransferase | NNMT | CfaAffx.20901.1.S1_at | 14,6 | 0,0042 |
| similar to Ig kappa chain V-II region 26-10 | LOC491391 | CfaAffx.23584.1.S1_at | 14,3 | 0,0045 |
| similar to mast cell antigen 32 | LOC480473 | CfaAffx.18235.1.S1_at | 13,9 | 0,0005 |
| major histocompatibility complex, class II, DQ alpha 1 | DLA-DQA1 | Cfa.182.1.S2_at | 13,8 | 0,0085 |
| C-type lectin domain family 5, member A | CLEC5A | CfaAffx.6745.1.S1_s_at | 13,7 | 0,0038 |
| triggering receptor expressed on myeloid cells 1 | TREM1 | CfaAffx.3292.1.S1_at | 13,7 | 0,0013 |
| similar to Ig lambda chain V-IV region Bau | LOC607368 | CfaAffx.21059.1.S1_at | 13,6 | 0,0158 |
| interferon stimulated exonuclease gene 20kDa | ISG20 | CfaAffx.17917.1.S1_at | 13,5 | 0,0003 |
| Rho GTPase activating protein 9 | ARHGAP9 | Cfa.11933.1.A1_at | 13,2 | 0,0004 |
| free fatty acid receptor 2 | FFAR2 | CfaAffx.11413.1.S1_at | 13,1 | 0,0002 |
| versican | VCAN | CfaAffx.13597.1.S1_s_at | 13,0 | 0,0018 |
| hemopoietic cell kinase | HCK | CfaAffx.11556.1.S1_at | 13,0 | 0,0002 |
| C-type lectin domain family 2, member D | CLEC2D | Cfa.18933.2.S1_s_at | 13,0 | 0,0050 |
| lymphocyte cytosolic protein 1 (L-plastin) | LCP1 | Cfa.19021.2.S1_s_at | 12,9 | 0,0006 |
| cathepsin S | CTSS | CfaAffx.18761.1.S1_s_at | 12,8 | 0,0015 |
| deoxyribonuclease I-like 3 | DNASE1L3 | CfaAffx.12004.1.S1_s_at | 12,7 | 0,0126 |
| ER degradation enhancer, mannosidase alpha-like 1 | EDEM1 | CfaAffx.9356.1.S1_at | 12,6 | 0,0053 |
| CD44 molecule (Indian blood group) | CD44 | Cfa.3800.2.S1_at | 12,6 | 0,0020 |
| guanylate binding protein 1, interferon-inducible, 67kDa | GBP1 | Cfa.18819.1.S1_at | 12,6 | 0,0007 |
| serum amyloid A1 | SAA1 | Cfa.3173.2.A1_x_at | 12,3 | 0,0162 |
| cathepsin H | CTSH | Cfa.2521.1.S1_at | 12,3 | 0,0031 |
| kynurenine 3-monooxygenase (kynurenine 3-hydroxylase) | KMO | Cfa.14036.1.A1_s_at | 12,3 | 0,0005 |
| matrix metallopeptidase 13 (collagenase 3) | MMP13 | CfaAffx.23153.1.S1_at | 12,1 | 0,0016 |
| lysosomal multispanning membrane protein 5 | LAPTM5 | Cfa.9004.1.S1_at | 11,9 | 0,0013 |
| solute carrier family 2 (facilitated glucose transporter), member 9 | SLC2A9 | Cfa.7132.1.A1_at | 11,8 | 0,0161 |
| integrin, beta 2 (complement component 3 receptor 3 and 4 subunit) | ITGB2 | Cfa.3634.1.S1_at | 11,8 | 0,0002 |
| chemokine (C-C motif) ligand 3 | CCL3 | Cfa.14352.1.A1_at | 11,8 | 0,0395 |
| ADAM metallopeptidase with thrombospondin type 1 motif, 2 | ADAMTS2 | Cfa.6326.1.A1_s_at | 11,5 | 0,0039 |
| CD53 molecule | CD53 | CfaAffx.30242.1.S1_at | 11,5 | 0,0002 |
| superoxide dismutase 2, mitochondrial | SOD2 | CfaAffx.1930.1.S1_s_at | 11,4 | 0,0003 |
| TIMP metallopeptidase inhibitor 1 | TIMP1 | Cfa.3680.1.S1_s_at | 11,3 | 0,0008 |
| apolipoprotein E | APOE | CfaAffx.7867.1.S1_s_at | 11,3 | 0,0180 |
| glia maturation factor, gamma | GMFG | CfaAffx.9311.1.S1_s_at | 11,2 | 0,0002 |
| G protein-coupled receptor 65 | GPR65 | CfaAffx.26530.1.S1_at | 11,2 | 0,0010 |
| interleukin 18 binding protein | IL18BP | Cfa.14516.1.S1_at | 11,1 | 0,0012 |
| major histocompatibility complex, class II, DQ beta 1 | DLA-DQB1 | CfaAffx.2152.1.S1_s_at | 11,0 | 0,0101 |
| coagulation factor II (thrombin) receptor-like 2 | F2RL2 | Cfa.10039.1.A1_at | 10,9 | 0,0227 |
| GTPase, IMAP family member 5 | GIMAP5 | CfaAffx.7820.1.S1_at | 10,9 | 0,0038 |
| MHC class II DR alpha chain | DLA-DRA1 | CfaAffx.2126.1.S1_s_at | 10,9 | 0,0047 |
| chemokine (C-C motif) ligand 14 | CCL14 | CfaAffx.27812.1.S1_s_at | 10,8 | 0,0005 |
| similar to Cytochrome P450 4F3 (CYPIVF3) (Leukotriene-B(4) omega-hydroxylase) | LOC484866 | CfaAffx.24426.1.S1_s_at | 10,8 | 0,0007 |
| chemokine (C-C motif) ligand 20 | CCL20 | CfaAffx.16422.1.S1_s_at | 10,7 | 0,0041 |
| complement factor properdin | CFP | CfaAffx.23322.1.S1_at | 10,5 | 0,0002 |
| dermatopontin | DPT | CfaAffx.23464.1.S1_s_at | 10,4 | 0,0214 |
| Gardner-Rasheed feline sarcoma viral (v-fgr) oncogene homolog | FGR | Cfa.13869.1.A1_at | 10,4 | 0,0002 |
| solute carrier family 2 (facilitated glucose transporter), member 3 | SLC2A3 | Cfa.825.1.S2_at | 10,4 | 0,0092 |
| solute carrier family 46, member 2 | SLC46A2 | Cfa.3.1.S1_s_at | 10,3 | 0,0237 |
| matrix metallopeptidase 7 (matrilysin, uterine) | MMP7 | CfaAffx.23201.1.S1_at | 10,2 | 0,0313 |
| plasminogen activator, urokinase receptor | PLAUR | CfaAffx.8028.1.S1_s_at | 10,2 | 0,0137 |
| CD68 molecule | CD68 | Cfa.13370.1.A1_at | 10,1 | 0,0008 |
| caspase 12 | LOC479458 | Cfa.13715.1.A1_at | 10,1 | 0,0018 |
| RAB20, member RAS oncogene family | RAB20 | Cfa.5163.1.A1_at | 10,0 | 0,0025 |
| mastin | LOC448801 | Cfa.6002.1.S1_at | 10,0 | 0,0074 |
| interleukin 1, beta | IL1B | Cfa.3554.1.S1_at | 9,9 | 0,0401 |
| transcobalamin I (vitamin B12 binding protein, R binder family) | TCN1 | CfaAffx.16182.1.S1_at | 9,7 | 0,0085 |
| fermitin family homolog 3 (Drosophila) | FERMT3 | CfaAffx.22546.1.S1_s_at | 9,7 | 0,0002 |
| MHC class Ib | DLA-79 | Cfa.14528.1.A1_at | 9,5 | 0,0157 |
| Rho GTPase activating protein 15 | ARHGAP15 | CfaAffx.9163.1.S1_at | 9,5 | 0,0002 |
| egf-like module containing, mucin-like, hormone receptor-like 3 | EMR3 | CfaAffx.24913.1.S1_at | 9,4 | 0,0002 |
| G protein-coupled receptor 110 | GPR110 | Cfa.14245.1.A1_s_at | 9,4 | 0,0151 |
| tumor necrosis factor (ligand) superfamily, member 13b | TNFSF13B | CfaAffx.10035.1.S1_s_at | 9,3 | 0,0103 |
| ankyrin repeat domain 22 | ANKRD22 | CfaAffx.24031.1.S1_at | 9,2 | 0,0002 |
| tenascin C | TNC | CfaAffx.6119.1.S1_s_at | 9,2 | 0,0118 |
| basic leucine zipper transcription factor, ATF-like | BATF | CfaAffx.26067.1.S1_s_at | 9,1 | 0,0040 |
| paired immunoglobin-like type 2 receptor alpha | PILRA | CfaAffx.22371.1.S1_at | 8,9 | 0,0005 |
| arachidonate 5-lipoxygenase-activating protein | ALOX5AP | CfaAffx.10828.1.S1_s_at | 8,9 | 0,0014 |
| guanine nucleotide binding protein (G protein), gamma 2 | GNG2 | CfaAffx.22567.1.S1_at | 8,8 | 0,0007 |
| toll-like receptor 2 | TLR2 | Cfa.15802.1.S1_at | 8,8 | 0,0002 |
| leukocyte immunoglobulin-like receptor, subfamily B (with TM and ITIM domains), member 2 | LILRB2 | CfaAffx.4831.1.S1_s_at | 8,7 | 0,0029 |
| similar to HLA class II histocompatibility antigen, gamma chain | LOC479329 | Cfa.1333.3.S1_s_at | 8,6 | 0,0090 |
| similar to Interferon-induced transmembrane protein 3 (Interferon-inducible protein 1-8U) | LOC475935 | Cfa.12215.1.A1_at | 8,6 | 0,0106 |
| solute carrier family 43, member 3 | SLC43A3 | Cfa.15550.1.A1_at | 8,6 | 0,0082 |
| ripply1 homolog (zebrafish) | RIPPLY1 | Cfa.17122.1.S1_at | 8,6 | 0,0400 |
| S100 calcium binding protein A4 | S100A4 | Cfa.3649.1.S1_s_at | 8,6 | 0,0025 |
| similar to T-cell surface glycoprotein CD1a precursor (CD1a antigen) | LOC608848 | Cfa.17811.1.S1_at | 8,5 | 0,0002 |
| selectin P (granule membrane protein 140kDa, antigen CD62) | SELP | CfaAffx.23346.1.S1_s_at | 8,5 | 0,0008 |
| FYN binding protein (FYB-120/130) | FYB | CfaAffx.741.1.S1_at | 8,5 | 0,0006 |
| prostaglandin E synthase | PTGES | CfaAffx.30585.1.S1_s_at | 8,5 | 0,0038 |
| Rho family GTPase 1 | RND1 | CfaAffx.13934.1.S1_at | 8,5 | 0,0038 |
| CD37 molecule | CD37 | CfaAffx.6506.1.S1_at | 8,4 | 0,0004 |
| peptidyl arginine deiminase, type IV | PADI4 | CfaAffx.24170.1.S1_at | 8,3 | 0,0031 |
| cystic fibrosis transmembrane conductance regulator | CFTR | Cfa.16181.1.S1_s_at | 8,3 | 0,0209 |
| fatty acid binding protein 5-like 2 | FABP5L2 | CfaAffx.109.1.S1_s_at | 8,3 | 0,0368 |
| TNFAIP3 interacting protein 3 | TNIP3 | CfaAffx.7159.1.S1_at | 8,2 | 0,0152 |
| diacylglycerol O-acyltransferase homolog 2 (mouse) | DGAT2 | Cfa.14378.1.S1_at | 8,2 | 0,0228 |
| parathyroid hormone-like hormone | PTHLH | Cfa.3856.1.S1_s_at | 8,1 | 0,0066 |
| similar to Glutathione S-transferase theta 2 (GST class-theta 2) | LOC477558 | Cfa.14362.1.S1_s_at | 8,1 | 0,0106 |
| complement factor D (adipsin) | CFD | Cfa.21381.1.S1_s_at | 8,1 | 0,0010 |
| acyl-Coenzyme A oxidase-like | ACOXL | CfaAffx.11490.1.S1_s_at | 7,9 | 0,0068 |
| leucine rich repeat containing 25 | LRRC25 | CfaAffx.22912.1.S1_at | 7,8 | 0,0029 |
| solute carrier family 1 (glutamate/neutral amino acid transporter), member 4 | SLC1A4 | Cfa.14889.1.A1_at | 7,8 | 0,0005 |
| MHC class II DLA DRB1 beta chain | DLA-DRB1 | Cfa.181.1.S1_at | 7,8 | 0,0052 |
| similar to FXYD domain-containing ion transport regulator 5 precursor (Dysadherin) | LOC612666 | Cfa.14945.1.S1_at | 7,7 | 0,0008 |
| lymphocyte antigen 9 | LY9 | CfaAffx.19583.1.S1_s_at | 7,7 | 0,0005 |
| CD38 molecule | CD38 | Cfa.3619.1.S1_s_at | 7,7 | 0,0268 |
| lymphocyte antigen 96 | LY96 | CfaAffx.816.1.S1_at | 7,6 | 0,0088 |
| fatty acid desaturase 1 | FADS1 | CfaAffx.24518.1.S1_at | 7,6 | 0,0015 |
| intercellular adhesion molecule 1 | ICAM1 | Cfa.3842.1.S1_at | 7,6 | 0,0062 |
| CD86 molecule | CD86 | Cfa.3629.2.S1_s_at | 7,6 | 0,0044 |
| interleukin 33 | IL33 | Cfa.3672.1.S1_s_at | 7,5 | 0,0039 |
| similar to basement membrane-induced gene | LOC612065 | Cfa.5919.1.A1_at | 7,5 | 0,0002 |
| linker for activation of T cells family, member 2 | LAT2 | CfaAffx.18652.1.S1_s_at | 7,5 | 0,0004 |
| spleen tyrosine kinase | SYK | CfaAffx.4145.1.S1_at | 7,4 | 0,0002 |
| phosphoprotein associated with glycosphingolipid microdomains 1 | PAG1 | Cfa.5284.1.A1_at | 7,4 | 0,0231 |
| RAB31, member RAS oncogene family | RAB31 | CfaAffx.28604.1.S1_s_at | 7,3 | 0,0014 |
| similar to family with sequence similarity 20, member A | LOC480458 | CfaAffx.17165.1.S1_at | 7,3 | 0,0387 |
| hydroxysteroid (11-beta) dehydrogenase 1 | HSD11B1 | CfaAffx.18536.1.S1_s_at | 7,3 | 0,0101 |
| complement factor B | CFB | Cfa.8846.1.A1_s_at | 7,3 | 0,0042 |
| adenosine deaminase | ADA | CfaAffx.14947.1.S1_at | 7,3 | 0,0004 |
| nidogen 2 (osteonidogen) | NID2 | Cfa.20443.1.S1_s_at | 7,2 | 0,0120 |
| dickkopf homolog 3 (Xenopus laevis) | DKK3 | Cfa.4454.1.S1_at | 7,2 | 0,0099 |
| regulator of G-protein signaling 1 | RGS1 | CfaAffx.16575.1.S1_s_at | 7,2 | 0,0109 |
| similar to C53D5.1a | LOC490155 | CfaAffx.30854.1.S1_at | 7,1 | 0,0013 |
| lymphocyte antigen 86 | LY86 | Cfa.15473.1.A1_at | 7,0 | 0,0008 |
| plasminogen activator, urokinase | PLAU | Cfa.127.1.S1_s_at | 7,0 | 0,0220 |
| lumican | LUM | CfaAffx.10198.1.S1_s_at | 7,0 | 0,0060 |
| solute carrier family 6 (amino acid transporter), member 14 | SLC6A14 | CfaAffx.27957.1.S1_at | 7,0 | 0,0096 |
| C-type lectin domain family 12, member A | CLEC12A | Cfa.9474.1.A1_at | 7,0 | 0,0027 |
| cholesterol 25-hydroxylase | CH25H | Cfa.16947.1.A1_at | 6,9 | 0,0178 |
| CD40 molecule, TNF receptor superfamily member 5 | CD40 | Cfa.180.1.S1_s_at | 6,7 | 0,0053 |
| neutrophil cytosolic factor 4, 40kDa | NCF4 | CfaAffx.3174.1.S1_s_at | 6,7 | 0,0002 |
| carbohydrate (N-acetylglucosamine 6-O) sulfotransferase 4 | CHST4 | CfaAffx.30912.1.S1_at | 6,7 | 0,0022 |
| phospholipid scramblase 1 | PLSCR1 | CfaAffx.13019.1.S1_s_at | 6,6 | 0,0062 |
| DEAD (Asp-Glu-Ala-Asp) box polypeptide 60 | DDX60 | CfaAffx.14223.1.S1_at | 6,5 | 0,0105 |
| major histocompatibility complex, class II, DM alpha | HLA-DMA | Cfa.18297.1.S1_at | 6,5 | 0,0038 |
| chemokine (C-C motif) receptor 5 | CCR5 | CfaAffx.21302.1.S1_s_at | 6,5 | 0,0002 |
| transcription factor EC | TFEC | Cfa.1175.1.A1_s_at | 6,4 | 0,0050 |
| complement component 1, q subcomponent, B chain | C1QB | Cfa.16857.1.S1_at | 6,4 | 0,0024 |
| ras-related C3 botulinum toxin substrate 2 (rho family, small GTP binding protein Rac2) | RAC2 | CfaAffx.3153.1.S1_s_at | 6,4 | 0,0004 |
| solute carrier family 2 (facilitated glucose transporter), member 6 | SLC2A6 | Cfa.6832.1.A1_at | 6,4 | 0,0002 |
| fibroblast activation protein, alpha | FAP | CfaAffx.16274.1.S1_at | 6,3 | 0,0007 |
| similar to glutathione S-transferase, pi 1 | LOC476006 | CfaAffx.17685.1.S1_s_at | 6,3 | 0,0013 |
| cadherin 11, type 2, OB-cadherin (osteoblast) | CDH11 | Cfa.458.2.S1_s_at | 6,3 | 0,0007 |
| interleukin 18 (interferon-gamma-inducing factor) | IL18 | Cfa.40.1.S1_s_at | 6,2 | 0,0461 |
| similar to retinoic acid receptor responder (tazarotene induced) 1 isoform 1 | LOC612298 | Cfa.12143.1.A1_at | 6,2 | 0,0048 |
| angiopoietin-like 4 | ANGPTL4 | Cfa.12323.1.A1_at | 6,2 | 0,0015 |
| cartilage oligomeric matrix protein | COMP | CfaAffx.22549.1.S1_s_at | 6,2 | 0,0313 |
| sorting nexin 10 | SNX10 | Cfa.17084.1.S1_at | 6,2 | 0,0020 |
| amyloid beta (A4) precursor protein-binding, family B, member 1 interacting protein | APBB1IP | CfaAffx.7409.1.S1_s_at | 6,1 | 0,0018 |
| lysyl oxidase-like 2 | LOXL2 | Cfa.12318.1.A1_at | 6,1 | 0,0009 |
| dual specificity phosphatase 5 | DUSP5 | CfaAffx.16846.1.S1_at | 6,1 | 0,0072 |
| prostate transmembrane protein, androgen induced 1 | PMEPA1 | Cfa.14230.1.A1_at | 6,0 | 0,0044 |
| GLI pathogenesis-related 1 | GLIPR1 | Cfa.5134.1.A1_s_at | 6,0 | 0,0002 |
| phosphotyrosine interaction domain containing 1 | PID1 | Cfa.11275.2.A1_s_at | 6,0 | 0,0106 |
| leucine-rich alpha-2-glycoprotein 1 | LRG1 | CfaAffx.29041.1.S1_at | 6,0 | 0,0011 |
| dehydrogenase/reductase (SDR family) member 9 | DHRS9 | CfaAffx.18687.1.S1_at | 5,9 | 0,0180 |
| surfactant protein D | SFTPD | CfaAffx.24148.1.S1_s_at | 5,9 | 0,0408 |
| ERO1-like (S. cerevisiae) | ERO1L | CfaAffx.22747.1.S1_s_at | 5,9 | 0,0183 |
| protein tyrosine phosphatase, non-receptor type 6 | PTPN6 | CfaAffx.22324.1.S1_s_at | 5,8 | 0,0001 |
| biliverdin reductase B (flavin reductase (NADPH)) | BLVRB | Cfa.14564.1.S1_s_at | 5,8 | 0,0049 |
| solute carrier family 11 (proton-coupled divalent metal ion transporters), member 1 | SLC11A1 | CfaAffx.22578.1.S1_s_at | 5,7 | 0,0002 |
| vav 1 guanine nucleotide exchange factor | VAV1 | CfaAffx.28423.1.S1_s_at | 5,7 | 0,0009 |
| lymphatic vessel endothelial hyaluronan receptor 1 | LYVE1 | CfaAffx.12229.1.S1_at | 5,6 | 0,0252 |
| tumor necrosis factor, alpha-induced protein 6 | TNFAIP6 | CfaAffx.9427.1.S1_at | 5,6 | 0,0072 |
| chemokine (C-X-C motif) receptor 4 | CXCR4 | CfaAffx.8579.1.S1_at | 5,6 | 0,0179 |
| V-set and immunoglobulin domain containing 4 | VSIG4 | CfaAffx.25438.1.S1_at | 5,5 | 0,0291 |
| TRAF3 interacting protein 3 | TRAF3IP3 | CfaAffx.18554.1.S1_at | 5,5 | 0,0004 |
| solute carrier family 46, member 3 | SLC46A3 | CfaAffx.10961.1.S1_at | 5,5 | 0,0091 |
| heparan sulfate (glucosamine) 3-O-sulfotransferase 3A1 | HS3ST3A1 | CfaAffx.27384.1.S1_s_at | 5,5 | 0,0114 |
| regulator of G-protein signaling 18 | RGS18 | CfaAffx.16582.1.S1_at | 5,5 | 0,0005 |
| CD14 molecule | CD14 | Cfa.6175.1.A1_at | 5,5 | 0,0008 |
| integrin, beta 7 | ITGB7 | Cfa.11961.1.A1_at | 5,4 | 0,0023 |
| ethanolamine kinase 1 | ETNK1 | CfaAffx.18122.1.S1_at | 5,4 | 0,0038 |
| ectonucleotide pyrophosphatase/phosphodiesterase 2 | ENPP2 | CfaAffx.2218.1.S1_s_at | 5,4 | 0,0332 |
| toll-like receptor 1 | TLR1 | CfaAffx.24815.1.S1_at | 5,4 | 0,0010 |
| transmembrane protein 173 | TMEM173 | CfaAffx.9512.1.S1_at | 5,4 | 0,0016 |
| protein tyrosine phosphatase, receptor type, C | PTPRC | CfaAffx.17631.1.S1_s_at | 5,4 | 0,0032 |
| heparan sulfate (glucosamine) 3-O-sulfotransferase 3B1 | HS3ST3B1 | CfaAffx.27403.1.S1_at | 5,4 | 0,0120 |
| actin related protein 2/3 complex, subunit 1B, 41kDa | ARPC1B | CfaAffx.23207.1.S1_s_at | 5,4 | 0,0015 |
| chondroitin sulfate synthase 3 | CHSY3 | Cfa.1562.1.A1_at | 5,3 | 0,0495 |
| proteasome (prosome, macropain) subunit, beta type, 8 (large multifunctional peptidase 7) | PSMB8 | Cfa.12298.1.A1_a_at | 5,3 | 0,0153 |
| fibulin 1 | FBLN1 | CfaAffx.2129.1.S1_s_at | 5,2 | 0,0487 |
| chemokine (C-X-C motif) ligand 13 (B-cell chemoattractant) | CXCL13 | CfaAffx.13739.1.S1_s_at | 5,2 | 0,0444 |
| T-cell immunoglobulin and mucin domain containing 4 | TIMD4 | CfaAffx.26902.1.S1_at | 5,1 | 0,0096 |
| 6-phosphofructo-2-kinase/fructose-2,6-biphosphatase 3 | PFKFB3 | Cfa.10187.1.A1_at | 5,1 | 0,0083 |
| nuclear receptor subfamily 1, group H, member 3 | NR1H3 | CfaAffx.13949.1.S1_s_at | 5,1 | 0,0168 |
| protease, serine, 23 | PRSS23 | CfaAffx.7537.1.S1_at | 5,1 | 0,0010 |
| crystallin, gamma S | CRYGS | CfaAffx.20903.1.S1_at | 5,1 | 0,0404 |
| growth differentiation factor 15 | GDF15 | CfaAffx.22917.1.S1_at | 5,0 | 0,0280 |
| nuclear factor of kappa light polypeptide gene enhancer in B-cells inhibitor, zeta | NFKBIZ | CfaAffx.15144.1.S1_s_at | 5,0 | 0,0016 |
| bridging integrator 2 | BIN2 | CfaAffx.12490.1.S1_at | 5,0 | 0,0002 |
| CD80 molecule | CD80 | Cfa.3596.2.S1_s_at | 5,0 | 0,0239 |
| similar to HLA class II histocompatibility antigen, DM beta chain precursor | LOC607827 | CfaAffx.2192.1.S1_at | 5,0 | 0,0030 |
| phosphoprotein associated with glycosphingolipid microdomains 1 | PAG1 | CfaAffx.13368.1.S1_s_at | 4,9 | 0,0097 |
| GLI pathogenesis-related 2 | GLIPR2 | CfaAffx.4299.1.S1_s_at | 4,9 | 0,0033 |
| protein S (alpha) | PROS1 | CfaAffx.14233.1.S1_s_at | 4,9 | 0,0044 |
| mannosidase, alpha, class 1A, member 1 | MAN1A1 | CfaAffx.2330.1.S1_s_at | 4,9 | 0,0065 |
| coagulation factor III (thromboplastin, tissue factor) | F3 | Cfa.16548.1.S1_at | 4,8 | 0,0428 |
| egf-like module containing, mucin-like, hormone receptor-like 4 pseudogene | EMR4P | Cfa.6138.1.A1_at | 4,8 | 0,0231 |
| opioid growth factor receptor-like 1 | OGFRL1 | CfaAffx.4788.1.S1_at | 4,8 | 0,0014 |
| sushi, von Willebrand factor type A, EGF and pentraxin domain containing 1 | SVEP1 | CfaAffx.5312.1.S1_s_at | 4,8 | 0,0317 |
| retinoic acid receptor responder (tazarotene induced) 3 | RARRES3 | CfaAffx.23214.1.S1_at | 4,8 | 0,0320 |
| serpin peptidase inhibitor, clade G (C1 inhibitor), member 1 | SERPING1 | CfaAffx.12561.1.S1_s_at | 4,8 | 0,0227 |
| 2'-5'-oligoadenylate synthetase 2, 69/71kDa | OAS2 | CfaAffx.14097.1.S1_s_at | 4,8 | 0,0107 |
| similar to serine/threonine protein kinase MASK | LOC492140 | Cfa.338.1.S1_a_at | 4,7 | 0,0022 |
| protein kinase C, beta | PRKCB | CfaAffx.26986.1.S1_at | 4,7 | 0,0004 |
| complement factor H | CFH | Cfa.20016.1.S1_s_at | 4,6 | 0,0107 |
| similar to immunity-related GTPase family, cinema 1 | LOC606875 | CfaAffx.19610.1.S1_at | 4,6 | 0,0279 |
| colony stimulating factor 2 receptor, alpha, low-affinity (granulocyte-macrophage) | CSF2RA | CfaAffx.17240.1.S1_at | 4,6 | 0,0022 |
| MHC class I DLA-12 /// MHC class I DLA-88 | DLA-12 /// dla88 | CfaAffx.1697.1.S1_s_at | 4,6 | 0,0206 |
| syndecan 2 | SDC2 | Cfa.5258.1.A1_at | 4,6 | 0,0013 |
| ADAM metallopeptidase with thrombospondin type 1 motif, 5 | ADAMTS5 | CfaAffx.13587.1.S1_at | 4,6 | 0,0351 |
| similar to angiopoietin-like 5 | LOC607055 | CfaAffx.1975.1.S1_at | 4,5 | 0,0357 |
| Fc fragment of IgE, high affinity I, receptor for; alpha polypeptide | FCER1A | Cfa.3663.1.A1_s_at | 4,5 | 0,0214 |
| MAX dimerization protein 1 | MXD1 | CfaAffx.5901.1.S1_at | 4,5 | 0,0054 |
| protein tyrosine phosphatase, receptor type, E | PTPRE | CfaAffx.20484.1.S1_s_at | 4,5 | 0,0055 |
| biglycan | BGN | Cfa.3763.1.S2_at | 4,5 | 0,0017 |
| C-type lectin domain family 4, member D | CLEC4D | CfaAffx.847.1.S1_at | 4,5 | 0,0180 |
| similar to CG4025-PA | LOC612179 | Cfa.6525.2.A1_s_at | 4,5 | 0,0074 |
| T-cell, immune regulator 1, ATPase, H+ transporting, lysosomal V0 subunit A3 | TCIRG1 | Cfa.11351.1.A1_at | 4,4 | 0,0004 |
| hexokinase 3 (white cell) | HK3 | CfaAffx.25391.1.S1_s_at | 4,4 | 0,0154 |
| integrin, alpha 1 | ITGA1 | CfaAffx.28209.1.S1_at | 4,4 | 0,0182 |
| granzyme B (granzyme 2, cytotoxic T-lymphocyte-associated serine esterase 1) | GZMB | CfaAffx.19332.1.S1_at | 4,4 | 0,0275 |
| phospholipase A2, group VII (platelet-activating factor acetylhydrolase, plasma) | PLA2G7 | Cfa.3709.1.S1_s_at | 4,4 | 0,0242 |
| interleukin 10 receptor, beta | IL10RB | Cfa.4778.1.A1_at | 4,4 | 0,0003 |
| chemokine (C-C motif) ligand 7 | CCL7 | Cfa.16337.1.S1_s_at | 4,4 | 0,0387 |
| complement component 1, r subcomponent | C1R | Cfa.4589.1.A1_s_at | 4,4 | 0,0203 |
| gamma-glutamyl hydrolase (conjugase, folylpolygammaglutamyl hydrolase) | GGH | Cfa.2326.1.S1_at | 4,4 | 0,0003 |
| baculoviral IAP repeat-containing 3 | BIRC3 | Cfa.18376.1.S1_at | 4,3 | 0,0367 |
| collagen, type V, alpha 2 | COL5A2 | Cfa.496.1.A1_s_at | 4,3 | 0,0014 |
| phospholipase C, gamma 2 (phosphatidylinositol-specific) | PLCG2 | CfaAffx.30601.1.S1_s_at | 4,3 | 0,0042 |
| gliomedin | GLDN | CfaAffx.23602.1.S1_s_at | 4,3 | 0,0209 |
| transmembrane protein 49 | TMEM49 | CfaAffx.27036.1.S1_at | 4,3 | 0,0018 |
| similar to mast cell-expressed membrane protein 1 | LOC611542 | CfaAffx.28021.1.S1_at | 4,3 | 0,0024 |
| G protein-coupled receptor 137B | GPR137B | CfaAffx.17337.1.S1_at | 4,2 | 0,0038 |
| monoamine oxidase B | MAOB | Cfa.14297.1.A1_s_at | 4,2 | 0,0041 |
| lysyl oxidase | LOX | CfaAffx.1719.1.S1_at | 4,2 | 0,0252 |
| similar to calmodulin-like 4 | LOC478351 | CfaAffx.26728.1.S1_at | 4,2 | 0,0051 |
| tetraspanin 4 | TSPAN4 | CfaAffx.15029.1.S1_at | 4,2 | 0,0339 |
| transferrin | TF | Cfa.2217.1.A1_at | 4,1 | 0,0040 |
| egf-like module containing, mucin-like, hormone receptor-like 1 | EMR1 | CfaAffx.28381.1.S1_s_at | 4,1 | 0,0134 |
| GRINL1A complex locus | GCOM1 | Cfa.7499.1.S1_at | 4,1 | 0,0090 |
| resistin | RETN | CfaAffx.28020.1.S1_s_at | 4,1 | 0,0295 |
| similar to Phosphatidylinositol 3,4,5-trisphosphate-dependent Rac exchanger 1 protein | LOC485917 | Cfa.19402.1.S1_at | 4,1 | 0,0007 |
| TIMP metallopeptidase inhibitor 2 | TIMP2 | Cfa.3497.1.A1_s_at | 4,1 | 0,0098 |
| coagulation factor II (thrombin) receptor | F2R | CfaAffx.14622.1.S1_at | 4,1 | 0,0341 |
| interleukin 22 receptor, alpha 2 | IL22RA2 | CfaAffx.1352.1.S1_s_at | 4,1 | 0,0120 |
| procollagen C-endopeptidase enhancer | PCOLCE | Cfa.21161.1.S1_s_at | 4,1 | 0,0139 |
| pyruvate dehydrogenase kinase, isozyme 1 | PDK1 | CfaAffx.20244.1.S1_at | 4,1 | 0,0004 |
| dihydropyrimidine dehydrogenase | DPYD | Cfa.20077.1.S1_at | 4,1 | 0,0033 |
| interleukin 16 (lymphocyte chemoattractant factor) | IL16 | CfaAffx.21374.1.S1_at | 4,1 | 0,0025 |
| acyl-CoA thioesterase 7 | ACOT7 | Cfa.11699.1.A1_at | 4,1 | 0,0095 |
| placenta-specific 8 | PLAC8 | CfaAffx.956.1.S1_at | 4,1 | 0,0045 |
| selectin E | SELE | Cfa.3868.1.S1_at | 4,1 | 0,0007 |
| NCK-associated protein 1-like | NCKAP1L | Cfa.17214.1.S1_s_at | 4,0 | 0,0008 |
| acyl-CoA synthetase long-chain family member 4 | ACSL4 | CfaAffx.27683.1.S1_s_at | 4,0 | 0,0260 |
| solute carrier family 39 (zinc transporter), member 8 | SLC39A8 | Cfa.12906.1.A1_s_at | 4,0 | 0,0278 |
| similar to [Pyruvate dehydrogenase [lipoamide]] kinase isozyme 1, mitochondrial precursor | PDK1 | CfaAffx.10453.1.S1_s_at | 4,0 | 0,0029 |
| cytokine receptor-like factor 3 | CRLF3 | Cfa.536.1.S1_s_at | 4,0 | 0,0003 |
| CD209 molecule | CD209 | Cfa.14490.1.S1_at | 4,0 | 0,0078 |
| transmembrane protein 45A | TMEM45A | CfaAffx.14696.1.S1_at | 3,9 | 0,0354 |
| nicotinamide phosphoribosyltransferase | NAMPT | Cfa.18345.1.S1_s_at | 3,9 | 0,0314 |
| PYD and CARD domain containing | PYCARD | CfaAffx.25782.1.S1_s_at | 3,9 | 0,0182 |
| major facilitator superfamily domain containing 2 | MFSD2 | CfaAffx.5474.1.S1_s_at | 3,8 | 0,0199 |
| basic leucine zipper transcription factor, ATF-like 3 | BATF3 | CfaAffx.19154.1.S1_at | 3,8 | 0,0183 |
| adipose differentiation-related protein | ADFP | Cfa.6339.1.A1_at | 3,8 | 0,0193 |
| myxovirus (influenza virus) resistance 2 (mouse) | MX2 | Cfa.3609.1.S1_s_at | 3,8 | 0,0171 |
| Rho GTPase activating protein 9 | ARHGAP9 | CfaAffx.1301.1.S1_s_at | 3,8 | 0,0011 |
| leucine rich repeat containing 8 family, member C | LRRC8C | CfaAffx.30934.1.S1_s_at | 3,8 | 0,0021 |
| collagen, type IV, alpha 1 | COL4A1 | CfaAffx.10085.1.S1_s_at | 3,8 | 0,0044 |
| secreted protein, acidic, cysteine-rich (osteonectin) | SPARC | CfaAffx.27328.1.S1_s_at | 3,8 | 0,0478 |
| procollagen C-endopeptidase enhancer 2 | PCOLCE2 | CfaAffx.12701.1.S1_at | 3,7 | 0,0260 |
| carbonic anhydrase IV | CA4 | Cfa.4077.1.S1_at | 3,7 | 0,0017 |
| collagen, type XV, alpha 1 | COL15A1 | CfaAffx.4658.1.S1_s_at | 3,7 | 0,0060 |
| glutathione S-transferase M1 | GSTM1 | Cfa.13231.1.A1_at | 3,7 | 0,0391 |
| endoglin (Osler-Rendu-Weber syndrome 1) | ENG | Cfa.13094.1.A1_at | 3,7 | 0,0068 |
| interleukin 1 receptor antagonist | IL1RN | Cfa.3496.1.S1_s_at | 3,6 | 0,0224 |
| peptidoglycan recognition protein 1 | PGLYRP1 | CfaAffx.7442.1.S1_at | 3,6 | 0,0105 |
| fatty acyl CoA reductase 1 | FAR1 | Cfa.1879.1.S1_s_at | 3,6 | 0,0004 |
| thioredoxin domain containing 16 | TXNDC16 | CfaAffx.22684.1.S1_at | 3,6 | 0,0291 |
| transmembrane protein 176A | TMEM176A | CfaAffx.7919.1.S1_at | 3,6 | 0,0068 |
| similar to sperm specific antigen 2 | LOC486489 | Cfa.11899.1.A1_at | 3,6 | 0,0070 |
| interleukin 18 receptor accessory protein | IL18RAP | CfaAffx.4100.1.S1_at | 3,6 | 0,0062 |
| calcium regulated heat stable protein 1, 24kDa | CARHSP1 | Cfa.8104.2.S1_s_at | 3,6 | 0,0046 |
| cysteine-rich protein 1 (intestinal) | CRIP1 | Cfa.6267.3.S1_s_at | 3,6 | 0,0187 |
| cathepsin D | CTSD | Cfa.17547.1.S1_at | 3,6 | 0,0044 |
| signal-regulatory protein alpha | SIRPA | CfaAffx.11069.1.S1_s_at | 3,6 | 0,0195 |
| annexin A1 | ANXA1 | CfaAffx.3571.1.S1_s_at | 3,6 | 0,0165 |
| Fc fragment of IgE, high affinity I, receptor for; gamma polypeptide | FCER1G | CfaAffx.19951.1.S1_s_at | 3,5 | 0,0015 |
| transforming growth factor, beta receptor II (70/80kDa) | TGFBR2 | CfaAffx.9108.1.S1_at | 3,5 | 0,0196 |
| sulfatase 1 | SULF1 | CfaAffx.12369.1.S1_s_at | 3,5 | 0,0015 |
| DEAD (Asp-Glu-Ala-Asp) box polypeptide 3, X-linked | DDX3X | CfaAffx.22006.1.S1_s_at | 3,5 | 0,0021 |
| TEA domain family member 4 | TEAD4 | CfaAffx.23839.1.S1_s_at | 3,5 | 0,0022 |
| similar to response gene to complement 32 | LOC609534 | Cfa.11204.1.A1_at | 3,5 | 0,0047 |
| WDFY family member 4 | WDFY4 | CfaAffx.10845.1.S1_at | 3,5 | 0,0014 |
| serine/threonine kinase 17b | STK17B | CfaAffx.16656.1.S1_s_at | 3,5 | 0,0389 |
| lipase, hepatic | LIPC | Cfa.13284.1.A1_at | 3,5 | 0,0457 |
| FK506 binding protein 1A, 12kDa | FKBP1A | CfaAffx.11117.1.S1_s_at | 3,5 | 0,0012 |
| myocyte enhancer factor 2C | MEF2C | Cfa.9073.1.A1_s_at | 3,4 | 0,0205 |
| poly (ADP-ribose) polymerase family, member 8 | PARP8 | Cfa.17149.1.S1_s_at | 3,4 | 0,0140 |
| aldehyde dehydrogenase 1 family, member L2 | ALDH1L2 | Cfa.11405.1.A1_at | 3,4 | 0,0075 |
| tumor necrosis factor receptor superfamily, member 14 (herpesvirus entry mediator) | TNFRSF14 | Cfa.6225.1.A1_at | 3,4 | 0,0022 |
| multiple EGF-like-domains 9 | MEGF9 | CfaAffx.6283.1.S1_at | 3,4 | 0,0136 |
| lymphoid-restricted membrane protein | LRMP | CfaAffx.17813.1.S1_s_at | 3,4 | 0,0246 |
| glutamate-cysteine ligase, modifier subunit | GCLM | Cfa.2376.1.A1_at | 3,4 | 0,0057 |
| CD180 molecule | CD180 | CfaAffx.12242.1.S1_at | 3,4 | 0,0013 |
| peptidase M20 domain containing 2 | PM20D2 | CfaAffx.5584.1.S1_at | 3,4 | 0,0259 |
| proline-serine-threonine phosphatase interacting protein 2 | PSTPIP2 | CfaAffx.27020.1.S1_at | 3,4 | 0,0103 |
| C-type lectin domain family 4, member E | CLEC4E | CfaAffx.21504.1.S1_s_at | 3,4 | 0,0252 |
| v-ral simian leukemia viral oncogene homolog A (ras related) | RALA | Cfa.9739.1.S1_at | 3,4 | 0,0030 |
| plasminogen activator, tissue | PLAT | CfaAffx.9314.1.S1_s_at | 3,4 | 0,0309 |
| transmembrane protein 2 | TMEM2 | Cfa.21578.1.S1_at | 3,4 | 0,0049 |
| similar to membrane cofactor protein isoform 12 precursor | LOC609365 | Cfa.21507.1.S1_at | 3,3 | 0,0039 |
| sterile alpha motif domain containing 9-like | SAMD9L | CfaAffx.3929.1.S1_at | 3,3 | 0,0200 |
| platelet derived growth factor D | PDGFD | Cfa.7017.1.A1_s_at | 3,3 | 0,0238 |
| olfactomedin-like 2B | OLFML2B | Cfa.14800.1.S1_at | 3,3 | 0,0044 |
| proteasome (prosome, macropain) subunit, beta type, 10 | PSMB10 | CfaAffx.31132.1.S1_at | 3,3 | 0,0030 |
| calmodulin 3 (phosphorylase kinase, delta) | CALM3 | Cfa.12086.1.S1_at | 3,3 | 0,0150 |
| solute carrier family 43, member 2 | SLC43A2 | Cfa.10890.1.A1_at | 3,3 | 0,0008 |
| fibronectin type III domain containing 3B | FNDC3B | CfaAffx.23611.1.S1_s_at | 3,3 | 0,0073 |
| fibulin 2 | FBLN2 | Cfa.6427.1.A1_at | 3,3 | 0,0280 |
| complement component 1, s subcomponent | C1S | CfaAffx.22221.1.S1_s_at | 3,3 | 0,0188 |
| mannosyl (alpha-1,3-)-glycoprotein beta-1,4-N-acetylglucosaminyltransferase, isozyme A | MGAT4A | CfaAffx.4348.1.S1_s_at | 3,3 | 0,0245 |
| cAMP responsive element modulator | CREM | Cfa.855.1.S1_at | 3,3 | 0,0019 |
| similar to tropomyosin 1 (alpha) | LOC609879 | CfaAffx.24216.1.S1_at | 3,3 | 0,0024 |
| basic helix-loop-helix domain containing, class B, 3 | BHLHB3 | Cfa.168.1.S1_at | 3,3 | 0,0342 |
| milk fat globule-EGF factor 8 protein | MFGE8 | Cfa.21061.1.S1_at | 3,3 | 0,0035 |
| insulin induced gene 1 | INSIG1 | Cfa.14020.1.A1_at | 3,2 | 0,0413 |
| SH3-domain binding protein 1 | SH3BP1 | CfaAffx.3122.1.S1_at | 3,2 | 0,0028 |
| serpin peptidase inhibitor, clade F, member 1 | SERPINF1 | CfaAffx.29358.1.S1_s_at | 3,2 | 0,0070 |
| regulator of G-protein signaling 19 | RGS19 | CfaAffx.23338.1.S1_at | 3,2 | 0,0011 |
| pleckstrin | PLEK | CfaAffx.5820.1.S1_s_at | 3,2 | 0,0269 |
| gap junction protein, alpha 5, 40kDa | GJA5 | Cfa.13792.1.A1_at | 3,2 | 0,0082 |
| membrane protein, palmitoylated 1, 55kDa | MPP1 | CfaAffx.30008.1.S1_s_at | 3,2 | 0,0030 |
| DnaJ (Hsp40) homolog, subfamily C, member 3 | DNAJC3 | Cfa.16320.1.A1_s_at | 3,2 | 0,0455 |
| transketolase | TKT | CfaAffx.13684.1.S1_s_at | 3,2 | 0,0118 |
| regulator of G-protein signaling 10 | RGS10 | Cfa.12424.1.A1_at | 3,2 | 0,0164 |
| selenoprotein X, 1 | SEPX1 | CfaAffx.29792.1.S1_s_at | 3,2 | 0,0307 |
| hydroxysteroid (17-beta) dehydrogenase 11 | HSD17B11 | CfaAffx.14976.1.S1_at | 3,2 | 0,0313 |
| von Willebrand factor | VWF | Cfa.111.1.A1_s_at | 3,2 | 0,0047 |
| CD248 molecule, endosialin | CD248 | Cfa.6421.1.A1_at | 3,2 | 0,0187 |
| similar to 3-oxo-5-beta-steroid 4-dehydrogenase | LOC609092 | CfaAffx.21321.1.S1_at | 3,1 | 0,0251 |
| vav 3 guanine nucleotide exchange factor | VAV3 | CfaAffx.30544.1.S1_s_at | 3,1 | 0,0295 |
| protein tyrosine phosphatase, receptor type, G | PTPRG | Cfa.10499.1.A1_at | 3,1 | 0,0116 |
| mitogen-activated protein kinase-activated protein kinase 2 | MAPKAPK2 | Cfa.21562.1.S1_s_at | 3,1 | 0,0030 |
| insulin-like growth factor binding protein 4 | IGFBP4 | CfaAffx.24660.1.S1_s_at | 3,1 | 0,0021 |
| cathepsin B | CTSB | Cfa.21450.1.S1_s_at | 3,1 | 0,0078 |
| carcinoembryonic antigen-related cell adhesion molecule 21 | CEACAM21 | CfaAffx.21987.1.S1_at | 3,1 | 0,0005 |
| dermatan sulfate epimerase | DSE | Cfa.12509.1.A1_s_at | 3,1 | 0,0404 |
| nuclear factor (erythroid-derived 2), 45kDa | NFE2 | CfaAffx.10806.1.S1_at | 3,1 | 0,0051 |
| PR domain containing 1, with ZNF domain | PRDM1 | CfaAffx.6485.1.S1_s_at | 3,1 | 0,0407 |
| insulin-like growth factor 2 mRNA binding protein 3 | IGF2BP3 | CfaAffx.5081.1.S1_at | 3,1 | 0,0007 |
| interferon gamma receptor 2 (interferon gamma transducer 1) | IFNGR2 | Cfa.8736.1.A1_x_at | 3,1 | 0,0018 |
| cylindromatosis (turban tumor syndrome) | CYLD | CfaAffx.15385.1.S1_s_at | 3,1 | 0,0038 |
| secretogranin III | SCG3 | Cfa.11197.1.A1_s_at | 3,1 | 0,0130 |
| PDZ and LIM domain 1 | PDLIM1 | CfaAffx.12933.1.S1_s_at | 3,1 | 0,0051 |
| 1-acylglycerol-3-phosphate O-acyltransferase 9 | AGPAT9 | Cfa.21318.2.S1_at | 3,1 | 0,0480 |
| carbohydrate (chondroitin 4) sulfotransferase 11 | CHST11 | CfaAffx.3818.1.S1_at | 3,1 | 0,0448 |
| lymphotoxin beta (TNF superfamily, member 3) | LTB | CfaAffx.1728.1.S1_s_at | 3,0 | 0,0212 |
| BCL2/adenovirus E1B 19kDa interacting protein 2 | BNIP2 | CfaAffx.25505.1.S1_s_at | 3,0 | 0,0082 |
| immediate early response 3 | IER3 | Cfa.16863.1.S1_at | 3,0 | 0,0447 |
| N-acylsphingosine amidohydrolase (acid ceramidase) 1 | ASAH1 | Cfa.1326.1.S1_at | 3,0 | 0,0179 |
| colony stimulating factor 3 receptor (granulocyte) | CSF3R | CfaAffx.5992.1.S1_at | 3,0 | 0,0018 |
| src kinase associated phosphoprotein 2 | SKAP2 | Cfa.18928.1.S1_at | 3,0 | 0,0472 |
| lysosomal-associated membrane protein 2 | LAMP2 | CfaAffx.28310.1.S1_at | 3,0 | 0,0088 |
| solute carrier family 1 (neutral amino acid transporter), member 5 | SLC1A5 | CfaAffx.7221.1.S1_s_at | 3,0 | 0,0017 |
| cyclin L2 | CCNL2 | Cfa.5916.1.A1_at | 3,0 | 0,0063 |
| similar to Ly6-B antigen gene | LOC475113 | Cfa.6278.1.S1_at | 3,0 | 0,0200 |
| similar to macrophage expressed gene 1 | LOC475960 | Cfa.20353.1.S1_at | 3,0 | 0,0095 |
| glutaminyl-peptide cyclotransferase | QPCT | Cfa.3719.1.S1_s_at | 3,0 | 0,0204 |
| ADAM metallopeptidase domain 28 | ADAM28 | CfaAffx.14362.1.S1_at | 3,0 | 0,0394 |
| Epstein-Barr virus induced gene 3 | EBI3 | CfaAffx.29182.1.S1_at | 3,0 | 0,0093 |
| chemokine (C-C motif) ligand 17 | CCL17 | Cfa.3450.1.S1_s_at | 3,0 | 0,0149 |
| similar to coactosin-like 1 | LOC489680 | CfaAffx.30530.1.S1_at | 2,9 | 0,0012 |
| interferon regulatory factor 1 | IRF1 | Cfa.18304.1.S1_at | 2,9 | 0,0092 |
| ST3 beta-galactoside alpha-2,3-sialyltransferase 6 | ST3GAL6 | CfaAffx.14496.1.S1_at | 2,9 | 0,0268 |
| claudin 1 | CLDN1 | CfaAffx.21607.1.S1_at | 2,9 | 0,0160 |
| plexin domain containing 2 | PLXDC2 | CfaAffx.7077.1.S1_at | 2,9 | 0,0025 |
| ferritin, light polypeptide | FTL | CfaAffx.6601.1.S1_x_at | 2,9 | 0,0049 |
| UDP-glucose ceramide glucosyltransferase | UGCG | CfaAffx.5454.1.S1_at | 2,9 | 0,0180 |
| metallothionein 3 | MT3 | Cfa.3275.1.S1_at | 2,9 | 0,0260 |
| SEC24 related gene family, member A (S. cerevisiae) | SEC24A | CfaAffx.2445.1.S1_at | 2,9 | 0,0070 |
| spleen focus forming virus (SFFV) proviral integration oncogene spi1 | SPI1 | CfaAffx.13801.1.S1_at | 2,9 | 0,0007 |
| beta-2-microglobulin | B2M | CfaAffx.21053.1.S1_s_at | 2,9 | 0,0154 |
| phospholipase A2, group XIIA | PLA2G12A | Cfa.8242.1.A1_s_at | 2,9 | 0,0056 |
| deoxyribonuclease II, lysosomal | DNASE2 | Cfa.4392.1.S1_at | 2,9 | 0,0013 |
| enolase 1, (alpha) | ENO1 | CfaAffx.30133.1.S1_s_at | 2,9 | 0,0062 |
| matrix metallopeptidase 2 (gelatinase A, 72kDa gelatinase, 72kDa type IV collagenase) | MMP2 | CfaAffx.14851.1.S1_s_at | 2,9 | 0,0324 |
| caspase 8, apoptosis-related cysteine peptidase | CASP8 | CfaAffx.18878.1.S1_s_at | 2,9 | 0,0070 |
| ubiquitin-conjugating enzyme E2 variant 1 | UBE2V1 | Cfa.11552.1.S1_a_at | 2,9 | 0,0009 |
| Lix1 homolog (mouse)-like | LIX1L | Cfa.11490.1.A1_at | 2,9 | 0,0137 |
| programmed cell death 1 ligand 2 | PDCD1LG2 | CfaAffx.4087.1.S1_at | 2,9 | 0,0178 |
| guanylate binding protein family, member 6 | GBP6 | CfaAffx.30939.1.S1_at | 2,9 | 0,0329 |
| ceroid-lipofuscinosis, neuronal 5 | cln5 | CfaAffx.8533.1.S1_s_at | 2,9 | 0,0057 |
| protein C receptor, endothelial (EPCR) | PROCR | CfaAffx.12741.1.S1_at | 2,9 | 0,0266 |
| fibronectin 1 | FN1 | Cfa.3707.3.S1_s_at | 2,9 | 0,0157 |
| integrin, alpha 9 | ITGA9 | CfaAffx.8185.1.S1_at | 2,9 | 0,0304 |
| formin-like 1 | FMNL1 | CfaAffx.21350.1.S1_s_at | 2,9 | 0,0012 |
| Ras association (RalGDS/AF-6) domain family (N-terminal) member 8 | RASSF8 | CfaAffx.17746.1.S1_s_at | 2,9 | 0,0122 |
| fibrillin 1 | FBN1 | Cfa.13066.1.A1_at | 2,9 | 0,0075 |
| GRAM domain containing 1A | GRAMD1A | CfaAffx.11622.1.S1_at | 2,8 | 0,0015 |
| similar to serine/threonine kinase 22 substrate 1 | LOC611897 | CfaAffx.6259.1.S1_at | 2,8 | 0,0276 |
| zinc finger, AN1-type domain 3 | ZFAND3 | CfaAffx.3116.1.S1_s_at | 2,8 | 0,0034 |
| myeloid cell nuclear differentiation antigen | MNDA | Cfa.16589.1.S1_at | 2,8 | 0,0135 |
| similar to AP2 associated kinase 1 | LOC612621 | Cfa.5958.1.A1_at | 2,8 | 0,0064 |
| hydrogen voltage-gated channel 1 | HVCN1 | Cfa.6077.1.S1_at | 2,8 | 0,0070 |
| interleukin 10 | IL10 | Cfa.38.1.S1_s_at | 2,8 | 0,0034 |
| actin binding LIM protein family, member 3 | ABLIM3 | Cfa.19264.1.S1_s_at | 2,8 | 0,0294 |
| syndecan 4 | SDC4 | CfaAffx.15202.1.S1_s_at | 2,8 | 0,0239 |
| transducin-like enhancer of split 4 (E(sp1) homolog, Drosophila) | TLE4 | Cfa.2411.1.A1_at | 2,8 | 0,0336 |
| 5-hydroxytryptamine (serotonin) receptor 2A | HTR2A | Cfa.3746.1.S1_s_at | 2,8 | 0,0352 |
| endothelin receptor type A | EDNRA | Cfa.500.1.S1_at | 2,8 | 0,0361 |
| hematological and neurological expressed 1 | HN1 | Cfa.10754.1.S1_at | 2,8 | 0,0336 |
| phytanoyl-CoA 2-hydroxylase interacting protein-like | PHYHIPL | Cfa.10925.1.S1_at | 2,8 | 0,0150 |
| collagen, type IV, alpha 2 | COL4A2 | CfaAffx.10091.1.S1_s_at | 2,8 | 0,0023 |
| sorting nexin 8 | SNX8 | CfaAffx.25156.1.S1_at | 2,8 | 0,0194 |
| succinate receptor 1 | SUCNR1 | CfaAffx.13553.1.S1_at | 2,8 | 0,0238 |
| G protein-coupled receptor 84 | GPR84 | CfaAffx.10729.1.S1_at | 2,7 | 0,0079 |
| similar to phosphoglycerate kinase 1 | LOC486305 | CfaAffx.16483.1.S1_s_at | 2,7 | 0,0203 |
| phosphoinositide-3-kinase adaptor protein 1 | PIK3AP1 | CfaAffx.14033.1.S1_at | 2,7 | 0,0296 |
| chloride intracellular channel 1 | CLIC1 | CfaAffx.4386.1.S1_s_at | 2,7 | 0,0044 |
| LIM and cysteine-rich domains 1 | LMCD1 | Cfa.12345.1.A1_at | 2,7 | 0,0239 |
| platelet/endothelial cell adhesion molecule | PECAM1 | CfaAffx.18281.1.S1_at | 2,7 | 0,0036 |
| ras homolog gene family, member H | RHOH | CfaAffx.24400.1.S1_at | 2,7 | 0,0377 |
| matrix-remodelling associated 5 | MXRA5 | CfaAffx.17427.1.S1_s_at | 2,7 | 0,0184 |
| ATPase type 13A4 | ATP13A4 | CfaAffx.476.1.S1_at | 2,7 | 0,0145 |
| transmembrane protein 165 | TMEM165 | Cfa.10592.1.A1_s_at | 2,7 | 0,0007 |
| Src-like-adaptor | SLA | CfaAffx.2569.1.S1_s_at | 2,7 | 0,0111 |
| ATPase, H+ transporting, lysosomal 56/58kDa, V1 subunit B2 | ATP6V1B2 | CfaAffx.15827.1.S1_s_at | 2,7 | 0,0220 |
| laminin, alpha 4 | LAMA4 | CfaAffx.6983.1.S1_at | 2,7 | 0,0044 |
| cathepsin K | CTSK | Cfa.588.1.S1_at | 2,7 | 0,0480 |
| arachidonate 5-lipoxygenase | ALOX5 | Cfa.8118.1.A1_s_at | 2,7 | 0,0105 |
| similar to ST3 beta-galactoside alpha-2,3-sialyltransferase 4 | LOC609517 | CfaAffx.16277.1.S1_at | 2,7 | 0,0096 |
| CD300 molecule-like family member f | CD300LF | CfaAffx.7838.1.S1_s_at | 2,7 | 0,0073 |
| transcobalamin II; macrocytic anemia | TCN2 | CfaAffx.19852.1.S1_s_at | 2,7 | 0,0038 |
| 2',5'-oligoadenylate synthetase 1, 40/46kDa | OAS1 | Cfa.21191.1.S1_a_at | 2,7 | 0,0273 |
| diacylglycerol lipase, beta | DAGLB | Cfa.2772.1.A1_at | 2,7 | 0,0407 |
| solute carrier family 39 (zinc transporter), member 14 | SLC39A14 | CfaAffx.14855.1.S1_s_at | 2,6 | 0,0025 |
| golgi transport 1 homolog B (S. cerevisiae) | GOLT1B | CfaAffx.19038.1.S1_s_at | 2,6 | 0,0497 |
| transient receptor potential cation channel, subfamily V, member 2 | TRPV2 | CfaAffx.27595.1.S1_at | 2,6 | 0,0007 |
| RAP1B, member of RAS oncogene family | RAP1B | Cfa.21068.1.S1_s_at | 2,6 | 0,0015 |
| serine hydroxymethyltransferase 2 (mitochondrial) | SHMT2 | Cfa.4302.1.S1_at | 2,6 | 0,0118 |
| fatty acid desaturase 3 | FADS3 | CfaAffx.24504.1.S1_s_at | 2,6 | 0,0206 |
| eukaryotic translation initiation factor 4E binding protein 1 | EIF4EBP1 | Cfa.11962.1.A1_at | 2,6 | 0,0142 |
| stress 70 protein chaperone, microsome-associated, 60kDa | STCH | Cfa.1481.1.S1_at | 2,6 | 0,0038 |
| arylsulfatase family, member J | ARSJ | CfaAffx.18890.1.S1_at | 2,6 | 0,0072 |
| ubiquitin-conjugating enzyme E2, J1 (UBC6 homolog, yeast) | UBE2J1 | Cfa.11661.1.A1_s_at | 2,6 | 0,0247 |
| transmembrane protein 154 | TMEM154 | CfaAffx.12992.1.S1_at | 2,6 | 0,0015 |
| regulator of G-protein signaling 2, 24kDa | RGS2 | Cfa.11085.1.A1_at | 2,6 | 0,0394 |
| leukocyte-associated immunoglobulin-like receptor 1 | LAIR1 | CfaAffx.4852.1.S1_at | 2,6 | 0,0336 |
| MHC class I DLA-12 | DLA-12 | Cfa.20996.1.S1_at | 2,6 | 0,0241 |
| coiled-coil domain containing 90A /// hypothetical LOC490542 | CCDC90A | CfaAffx.556.1.S1_s_at | 2,6 | 0,0473 |
| Niemann-Pick disease, type C2 | NPC2 | Cfa.3781.1.S1_at | 2,6 | 0,0497 |
| dedicator of cytokinesis 8 | DOCK8 | CfaAffx.3864.1.S1_at | 2,6 | 0,0292 |
| CD59 molecule, complement regulatory protein | CD59 | CfaAffx.11657.1.S1_s_at | 2,6 | 0,0259 |
| peripherin 2 (retinal degeneration, slow) | PRPH2 | Cfa.3520.1.S2_s_at | 2,6 | 0,0031 |
| BTB and CNC homology 1, basic leucine zipper transcription factor 1 | BACH1 | CfaAffx.13809.1.S1_s_at | 2,6 | 0,0158 |
| regulator of G-protein signaling 5 | RGS5 | Cfa.21159.1.S1_at | 2,6 | 0,0096 |
| aminoadipate aminotransferase | AADAT | CfaAffx.12380.1.S1_at | 2,6 | 0,0070 |
| similar to TCDD-inducible poly(ADP-ribose) polymerase | LOC608673 | CfaAffx.18567.1.S1_s_at | 2,6 | 0,0235 |
| aminolevulinate, delta-, synthase 1 | ALAS1 | Cfa.2383.1.S1_s_at | 2,6 | 0,0475 |
| DENN/MADD domain containing 4A | DENND4A | CfaAffx.26383.1.S1_s_at | 2,6 | 0,0169 |
| dual adaptor of phosphotyrosine and 3-phosphoinositides | DAPP1 | CfaAffx.16527.1.S1_at | 2,6 | 0,0124 |
| polo-like kinase 3 (Drosophila) | PLK3 | Cfa.12767.1.A1_at | 2,6 | 0,0116 |
| proprotein convertase subtilisin/kexin type 5 | PCSK5 | Cfa.12028.1.A1_s_at | 2,6 | 0,0098 |
| intersectin 1 (SH3 domain protein) | ITSN1 | Cfa.15687.1.A1_at | 2,6 | 0,0018 |
| lipopolysaccharide-induced TNF factor /// similar to LPS-induced TN factor | LITAF | CfaAffx.28872.1.S1_at | 2,6 | 0,0010 |
| tyrosine 3-monooxygenase/tryptophan 5-monooxygenase activation protein, gamma polypeptide | YWHAG | Cfa.15085.1.A1_at | 2,6 | 0,0022 |
| malic enzyme 1, NADP(+)-dependent, cytosolic | ME1 | Cfa.3572.1.S1_s_at | 2,6 | 0,0339 |
| signal transducer and activator of transcription 1, 91kDa | STAT1 | Cfa.18084.1.S1_s_at | 2,5 | 0,0173 |
| ARP2 actin-related protein 2 homolog (yeast) | ACTR2 | CfaAffx.5751.1.S1_at | 2,5 | 0,0151 |
| Rab interacting lysosomal protein-like 2 | RILPL2 | CfaAffx.12099.1.S1_at | 2,5 | 0,0155 |
| oxysterol binding protein-like 3 | OSBPL3 | CfaAffx.5199.1.S1_s_at | 2,5 | 0,0327 |
| similar to CG32066-PB, isoform B | LOC482041 | Cfa.20646.1.S1_s_at | 2,5 | 0,0014 |
| ATPase, Na+/K+ transporting, beta 3 polypeptide | ATP1B3 | Cfa.9984.1.A1_at | 2,5 | 0,0261 |
| interleukin 15 | IL15 | CfaAffx.6380.1.S1_s_at | 2,5 | 0,0109 |
| stabilin 1 | STAB1 | CfaAffx.14701.1.S1_s_at | 2,5 | 0,0018 |
| calumenin | CALU | Cfa.3402.3.S1_a_at | 2,5 | 0,0020 |
| neuregulin 1 | NRG1 | Cfa.9043.1.A1_at | 2,5 | 0,0102 |
| acyl-CoA synthetase short-chain family member 1 | ACSS1 | Cfa.16751.1.S1_at | 2,5 | 0,0370 |
| apolipoprotein B mRNA editing enzyme, catalytic polypeptide-like 3H | APOBEC3H | CfaAffx.2928.1.S1_at | 2,5 | 0,0057 |
| ubiquitin-conjugating enzyme E2H (UBC8 homolog, yeast) | UBE2H | CfaAffx.3148.1.S1_at | 2,5 | 0,0049 |
| N-acetylneuraminate pyruvate lyase (dihydrodipicolinate synthase) | NPL | CfaAffx.20382.1.S1_at | 2,5 | 0,0252 |
| ATP citrate lyase | ACLY | CfaAffx.24270.1.S1_s_at | 2,5 | 0,0172 |
| SAR1 gene homolog A (S. cerevisiae) | SAR1A | Cfa.6062.1.A1_s_at | 2,5 | 0,0069 |
| MHC class I DLA-12 /// MHC class I DLA-64 /// MHC class I DLA-88 | DLA-12 | Cfa.280.1.S1_s_at | 2,5 | 0,0175 |
| similar to Complement C4 precursor | LOC481722 | CfaAffx.1993.1.S1_s_at | 2,5 | 0,0096 |
| guanine nucleotide binding protein (G protein), gamma 11 | GNG11 | Cfa.16313.1.S1_at | 2,5 | 0,0259 |
| solute carrier family 16, member 13 (monocarboxylic acid transporter 13) | SLC16A13 | Cfa.4733.1.S1_at | 2,5 | 0,0090 |
| major vault protein | MVP | Cfa.20295.1.S1_s_at | 2,5 | 0,0130 |
| TBC1 domain family, member 14 | TBC1D14 | CfaAffx.22154.1.S1_at | 2,5 | 0,0243 |
| microfibrillar-associated protein 4 | MFAP4 | CfaAffx.27870.1.S1_s_at | 2,4 | 0,0500 |
| similar to putative MAPK activating protein PM20,PM21 isoform 2 | LOC608518 | Cfa.15402.1.A1_s_at | 2,4 | 0,0099 |
| interleukin 17A | IL17A | CfaAffx.4159.1.S1_at | 2,4 | 0,0202 |
| similar to BTB (POZ) domain containing 1 isoform 1 | LOC479057 | Cfa.12692.1.A1_at | 2,4 | 0,0139 |
| toll-like receptor 4 | TLR4 | Cfa.118.1.S1_at | 2,4 | 0,0409 |
| mitogen-activated protein kinase 13 | MAPK13 | CfaAffx.2951.1.S1_at | 2,4 | 0,0026 |
| intercellular adhesion molecule 2 | ICAM2 | CfaAffx.18328.1.S1_s_at | 2,4 | 0,0042 |
| actin binding LIM protein family, member 3 | ABLIM3 | CfaAffx.28057.1.S1_x_at | 2,4 | 0,0163 |
| phosphoinositide-3-kinase, catalytic, delta polypeptide | PIK3CD | CfaAffx.30189.1.S1_s_at | 2,4 | 0,0014 |
| uridine-cytidine kinase 2 | UCK2 | CfaAffx.20837.1.S1_at | 2,4 | 0,0126 |
| similar to fasting-inducible integral membrane protein TM6P1 | LOC611328 | CfaAffx.4754.1.S1_at | 2,4 | 0,0295 |
| SH3-domain kinase binding protein 1 | SH3KBP1 | Cfa.10263.1.A1_at | 2,4 | 0,0031 |
| BTG family, member 2 | BTG2 | CfaAffx.14883.1.S1_at | 2,4 | 0,0263 |
| RAS p21 protein activator 3 | RASA3 | Cfa.539.1.S1_at | 2,4 | 0,0158 |
| NAD(P)H dehydrogenase, quinone 1 | NQO1 | Cfa.16827.1.A1_at | 2,4 | 0,0230 |
| S100 calcium binding protein A10 | S100A10 | Cfa.8946.1.A1_at | 2,4 | 0,0084 |
| solute carrier family 16, member 3 (monocarboxylic acid transporter 4) | SLC16A3 | CfaAffx.10063.1.S1_at | 2,4 | 0,0066 |
| ring finger protein 19B | RNF19B | CfaAffx.16271.1.S1_s_at | 2,4 | 0,0332 |
| similar to NIMA (never in mitosis gene a)-related expressed kinase 6 | NEK6 | CfaAffx.30976.1.S1_at | 2,4 | 0,0135 |
| phosphoglycerate mutase 1 (brain) | PGAM1 | CfaAffx.14307.1.S1_s_at | 2,4 | 0,0490 |
| similar to Thioredoxin (ATL-derived factor) (ADF) (Surface associated sulphydryl protein) (SASP) | LOC474798 | Cfa.9924.1.A1_at | 2,4 | 0,0091 |
| dedicator of cytokinesis 2 | DOCK2 | CfaAffx.25977.1.S1_at | 2,4 | 0,0062 |
| sushi-repeat-containing protein, X-linked 2 | SRPX2 | CfaAffx.26798.1.S1_at | 2,4 | 0,0118 |
| sema domain, seven thrombospondin repeats (type 1 and type 1-like) | SEMA5A | CfaAffx.15830.1.S1_s_at | 2,4 | 0,0062 |
| transmembrane 4 L six family member 18 | TM4SF18 | Cfa.1980.1.S1_at | 2,4 | 0,0125 |
| ST6 (alpha-N-acetyl-neuraminyl-2,3-beta-galactosyl-1,3)-N-acetylgalactosaminide alpha-2,6-sialyltransferase 4 | ST6GALNAC4 | CfaAffx.30768.1.S1_s_at | 2,4 | 0,0055 |
| procollagen-proline, 2-oxoglutarate 4-dioxygenase (proline 4-hydroxylase), alpha polypeptide I | P4HA1 | CfaAffx.22481.1.S1_at | 2,4 | 0,0193 |
| colony stimulating factor 1 receptor | CSF1R | CfaAffx.27899.1.S1_at | 2,4 | 0,0023 |
| actin related protein 2/3 complex, subunit 5, 16kDa | ARPC5 | Cfa.18603.1.S1_at | 2,4 | 0,0175 |
| similar to glutathione S-transferase, theta 3 | LOC477555 | Cfa.15273.1.A1_at | 2,4 | 0,0353 |
| ras homolog gene family, member G (rho G) | RHOG | Cfa.7898.1.A1_at | 2,4 | 0,0007 |
| mitogen-activated protein kinase kinase 3 | MAP2K3 | Cfa.18291.1.S1_s_at | 2,4 | 0,0070 |
| meteorin, glial cell differentiation regulator-like | METRNL | CfaAffx.20296.1.S1_at | 2,4 | 0,0103 |
| ethylmalonic encephalopathy 1 | ETHE1 | CfaAffx.8070.1.S1_at | 2,4 | 0,0066 |
| lysophosphatidylcholine acyltransferase 2 | LPCAT2 | CfaAffx.14833.1.S1_at | 2,4 | 0,0247 |
| G-protein signaling modulator 3 (AGS3-like, C. elegans) | GPSM3 | Cfa.10002.1.A1_at | 2,4 | 0,0051 |
| CD97 molecule | CD97 | CfaAffx.25091.1.S1_s_at | 2,4 | 0,0006 |
| HUS1 checkpoint homolog (S. pombe) | HUS1 | Cfa.11015.1.A1_at | 2,4 | 0,0225 |
| GTPase, IMAP family member 7 | GIMAP7 | Cfa.19158.1.S1_s_at | 2,3 | 0,0482 |
| RAB8B, member RAS oncogene family | RAB8B | Cfa.15215.1.A1_at | 2,3 | 0,0352 |
| protein kinase, cAMP-dependent, regulatory, type I, alpha (tissue specific extinguisher 1) | PRKAR1A | Cfa.11995.1.A1_at | 2,3 | 0,0015 |
| ST8 alpha-N-acetyl-neuraminide alpha-2,8-sialyltransferase 4 | ST8SIA4 | CfaAffx.12274.1.S1_at | 2,3 | 0,0349 |
| ubiquitin-fold modifier 1 | UFM1 | Cfa.11736.1.A1_at | 2,3 | 0,0033 |
| similar to eukaryotic translation termination factor 1 (predicted) | LOC608717 | CfaAffx.2814.1.S1_s_at | 2,3 | 0,0087 |
| 4-aminobutyrate aminotransferase | ABAT | Cfa.4397.1.A1_s_at | 2,3 | 0,0037 |
| transmembrane protein 156 | TMEM156 | CfaAffx.24785.1.S1_at | 2,3 | 0,0189 |
| transforming growth factor, beta 1 | TGFB1 | Cfa.3509.1.S1_s_at | 2,3 | 0,0082 |
| amyloid beta (A4) precursor protein | APP | Cfa.106.1.S1_s_at | 2,3 | 0,0258 |
| immunoglobulin superfamily, member 9 | IGSF9 | CfaAffx.18427.1.S1_at | 2,3 | 0,0124 |
| kinase insert domain receptor (a type III receptor tyrosine kinase) | KDR | CfaAffx.4040.1.S1_s_at | 2,3 | 0,0239 |
| phosphatidylglycerophosphate synthase 1 | PGS1 | CfaAffx.8916.1.S1_at | 2,3 | 0,0164 |
| latexin | LXN | Cfa.5195.1.A1_s_at | 2,3 | 0,0056 |
| phospholipase A2, group IVA (cytosolic, calcium-dependent) | PLA2G4A | Cfa.2937.1.A1_at | 2,3 | 0,0302 |
| moesin | MSN | CfaAffx.25424.1.S1_s_at | 2,3 | 0,0004 |
| coatomer protein complex, subunit zeta 2 | COPZ2 | CfaAffx.25723.1.S1_s_at | 2,3 | 0,0430 |
| NLR family, pyrin domain containing 3 | NLRP3 | CfaAffx.16724.1.S1_s_at | 2,3 | 0,0017 |
| solute carrier family 36 (proton/amino acid symporter), member 4 | SLC36A4 | CfaAffx.7283.1.S1_at | 2,3 | 0,0158 |
| interferon gamma receptor 1 | IFNGR1 | CfaAffx.1357.1.S1_at | 2,3 | 0,0247 |
| inter-alpha (globulin) inhibitor H3 | ITIH3 | Cfa.8496.1.A1_at | 2,3 | 0,0406 |
| CAP, adenylate cyclase-associated protein 1 (yeast) | CAP1 | Cfa.18169.1.S1_s_at | 2,3 | 0,0077 |
| cytoskeleton-associated protein 4 | CKAP4 | CfaAffx.3733.1.S1_at | 2,3 | 0,0207 |
| WAS/WASL interacting protein family, member 1 | WIPF1 | CfaAffx.20533.1.S1_at | 2,3 | 0,0019 |
| signal transducing adaptor molecule (SH3 domain and ITAM motif) 2 | STAM2 | Cfa.13041.1.A1_s_at | 2,3 | 0,0062 |
| phosphoglycerate kinase 1 | PGK1 | CfaAffx.26428.1.S1_s_at | 2,3 | 0,0113 |
| glycyl-tRNA synthetase | GARS | Cfa.3929.1.A1_a_at | 2,3 | 0,0010 |
| zinc finger, AN1-type domain 2A | ZFAND2A | Cfa.5462.1.A1_at | 2,3 | 0,0291 |
| chondroitin sulfate N-acetylgalactosaminyltransferase 2 | CSGALNACT2 | CfaAffx.11464.1.S1_at | 2,3 | 0,0325 |
| lactamase, beta | LACTB | CfaAffx.25961.1.S1_at | 2,3 | 0,0072 |
| transmembrane 4 L six family member 1 | TM4SF1 | CfaAffx.13236.1.S1_at | 2,3 | 0,0163 |
| signaling lymphocytic activation molecule family member 1 | SLAMF1 | CfaAffx.19514.1.S1_s_at | 2,3 | 0,0024 |
| mucin 5B, oligomeric mucus/gel-forming | MUC5B | CfaAffx.15591.1.S1_s_at | 2,3 | 0,0007 |
| transmembrane protein 30A | TMEM30A | CfaAffx.4943.1.S1_s_at | 2,3 | 0,0042 |
| potassium voltage-gated channel, Isk-related family, member 3 | KCNE3 | CfaAffx.9180.1.S1_s_at | 2,3 | 0,0249 |
| insulin-like growth factor binding protein 7 | IGFBP7 | CfaAffx.4359.1.S1_s_at | 2,3 | 0,0037 |
| solute carrier family 23 (nucleobase transporters), member 2 | SLC23A2 | CfaAffx.10036.1.S1_at | 2,3 | 0,0095 |
| RAS guanyl releasing protein 3 (calcium and DAG-regulated) | RASGRP3 | CfaAffx.9761.1.S1_at | 2,3 | 0,0488 |
| growth factor receptor-bound protein 2 | GRB2 | Cfa.2172.1.S1_at | 2,3 | 0,0043 |
| WD repeat domain 1 | WDR1 | Cfa.1345.1.A1_at | 2,3 | 0,0103 |
| MAP/microtubule affinity-regulating kinase 1 | MARK1 | Cfa.5423.1.A1_s_at | 2,3 | 0,0343 |
| ERO1-like beta (S. cerevisiae) | ERO1LB | CfaAffx.17310.1.S1_s_at | 2,3 | 0,0063 |
| N-acetylglucosaminidase, alpha- | NAGLU | CfaAffx.23020.1.S1_at | 2,3 | 0,0116 |
| abhydrolase domain containing 2 | ABHD2 | Cfa.436.3.S1_a_at | 2,3 | 0,0105 |
| MIF4G domain containing | MIF4GD | Cfa.4092.1.S1_at | 2,3 | 0,0039 |
| glycosyltransferase 25 domain containing 1 | GLT25D1 | Cfa.18369.1.S1_s_at | 2,3 | 0,0076 |
| cOR51C4 olfactory receptor family 51 subfamily C-like | cOR51C4 | Cfa.15865.1.S1_s_at | 2,3 | 0,0030 |
| protein tyrosine phosphatase type IVA, member 3 | PTP4A3 | Cfa.16315.1.S1_at | 2,3 | 0,0049 |
| mitogen-activated protein kinase kinase kinase kinase 4 | MAP4K4 | CfaAffx.4144.1.S1_s_at | 2,3 | 0,0397 |
| proenkephalin | PENK | Cfa.1221.1.S1_s_at | 2,2 | 0,0301 |
| serine/threonine kinase 10 | STK10 | Cfa.15271.1.A1_at | 2,2 | 0,0066 |
| karyopherin alpha 4 (importin alpha 3) | KPNA4 | CfaAffx.375.1.S1_at | 2,2 | 0,0295 |
| interleukin 34 | IL34 | CfaAffx.30861.1.S1_at | 2,2 | 0,0123 |
| lactate dehydrogenase A | LDHA | Cfa.300.1.S1_at | 2,2 | 0,0053 |
| similar to Tumor necrosis factor receptor superfamily member Fn14 precursor | LOC610734 | CfaAffx.118.1.S1_s_at | 2,2 | 0,0011 |
| similar to Ferritin light chain 2 (Ferritin L subunit 2) (Ferritin subunit LG) | LOC479746 | CfaAffx.900.1.S1_at | 2,2 | 0,0047 |
| glucosidase, beta; acid (includes glucosylceramidase) | GBA | CfaAffx.25986.1.S1_s_at | 2,2 | 0,0041 |
| adenylate cyclase 4 | ADCY4 | CfaAffx.19151.1.S1_s_at | 2,2 | 0,0046 |
| chloride intracellular channel 4 | CLIC4 | CfaAffx.20009.1.S1_at | 2,2 | 0,0178 |
| STEAP family member 4 | STEAP4 | CfaAffx.3699.1.S1_s_at | 2,2 | 0,0345 |
| solute carrier family 25 (mitochondrial carrier; phosphate carrier), member 24 | SLC25A24 | CfaAffx.30511.1.S1_s_at | 2,2 | 0,0177 |
| protein disulfide isomerase family A, member 5 | PDIA5 | Cfa.10786.1.A1_s_at | 2,2 | 0,0259 |
| similar to cat eye syndrome critical region protein 1 isoform a precursor | LOC484249 | Cfa.7093.1.A1_at | 2,2 | 0,0054 |
| phosphogluconate dehydrogenase | PGD | Cfa.11536.1.A1_at | 2,2 | 0,0020 |
| CD164 molecule, sialomucin | CD164 | CfaAffx.6642.1.S1_s_at | 2,2 | 0,0340 |
| sorting nexin 7 | SNX7 | CfaAffx.30694.1.S1_s_at | 2,2 | 0,0270 |
| triosephosphate isomerase 1 | TPI1 | Cfa.6532.1.A1_at | 2,2 | 0,0055 |
| signal sequence receptor, alpha | SSR1 | Cfa.1295.1.S1_at | 2,2 | 0,0441 |
| lymphocyte antigen 75 | LY75 | CfaAffx.15106.1.S1_s_at | 2,2 | 0,0468 |
| protease, serine-like 1 | PRSSL1 | CfaAffx.30141.1.S1_at | 2,2 | 0,0112 |
| ankyrin repeat domain 10 | ANKRD10 | CfaAffx.10140.1.S1_at | 2,2 | 0,0286 |
| ribonuclease T2 | RNASET2 | CfaAffx.2188.1.S1_at | 2,2 | 0,0250 |
| CDC42 effector protein (Rho GTPase binding) 5 | CDC42EP5 | CfaAffx.856.1.S1_at | 2,2 | 0,0093 |
| cOR9K3 olfactory receptor family 9 subfamily K-like | cOR9K3 | CfaAffx.14945.1.S1_s_at | 2,2 | 0,0104 |
| ST3 beta-galactoside alpha-2,3-sialyltransferase 4 | ST3GAL4 | CfaAffx.16277.1.S1_s_at | 2,2 | 0,0320 |
| adenosine A2a receptor | ADORA2A | Cfa.3818.1.S1_s_at | 2,2 | 0,0017 |
| spermatogenesis associated 6 | SPATA6 | Cfa.7626.1.S1_s_at | 2,2 | 0,0372 |
| similar to septin 4 isoform 2 | LOC480571 | Cfa.1791.1.A1_s_at | 2,2 | 0,0205 |
| FXYD domain containing ion transport regulator 6 | FXYD6 | Cfa.9325.1.A1_s_at | 2,2 | 0,0338 |
| solute carrier family 38, member 1 | SLC38A1 | Cfa.10484.1.S1_s_at | 2,2 | 0,0344 |
| actin related protein 2/3 complex, subunit 2, 34kDa | ARPC2 | CfaAffx.22531.1.S1_at | 2,2 | 0,0036 |
| proteolipid protein 2 (colonic epithelium-enriched) | PLP2 | Cfa.5295.1.A1_at | 2,2 | 0,0068 |
| collagen, type XVI, alpha 1 | COL16A1 | CfaAffx.17101.1.S1_s_at | 2,2 | 0,0262 |
| thiamin pyrophosphokinase 1 | TPK1 | Cfa.7319.1.A1_at | 2,2 | 0,0198 |
| sulfatase 2 | SULF2 | Cfa.16398.1.S1_at | 2,2 | 0,0230 |
| coiled-coil domain containing 50 | CCDC50 | Cfa.10657.1.A1_at | 2,2 | 0,0015 |
| peptidylprolyl isomerase F (cyclophilin F) | PPIF | Cfa.16705.1.S1_at | 2,2 | 0,0313 |
| CD200 receptor 1 | CD200R1 | CfaAffx.16494.1.S1_at | 2,2 | 0,0289 |
| phosphofructokinase, platelet | PFKP | Cfa.10684.2.A1_s_at | 2,2 | 0,0071 |
| phosphatase and tensin homolog | PTEN | Cfa.3695.1.S1_s_at | 2,2 | 0,0062 |
| carnitine palmitoyltransferase 1C | CPT1C | Cfa.20638.1.S1_at | 2,2 | 0,0118 |
| laminin, gamma 1 (formerly LAMB2) | LAMC1 | CfaAffx.20472.1.S1_s_at | 2,1 | 0,0058 |
| hematopoietically expressed homeobox | HHEX | CfaAffx.12123.1.S1_at | 2,1 | 0,0216 |
| Nedd4 family interacting protein 2 | NDFIP2 | CfaAffx.8839.1.S1_s_at | 2,1 | 0,0453 |
| similar to CG30152-PA | LOC478335 | Cfa.10504.1.S1_s_at | 2,1 | 0,0391 |
| ribonuclease, RNase A family, 1 (pancreatic) | RNASE1 | Cfa.6188.1.A1_at | 2,1 | 0,0078 |
| alanyl (membrane) aminopeptidase | ANPEP | Cfa.3774.1.A1_s_at | 2,1 | 0,0126 |
| microtubule-associated protein tau | MAPT | Cfa.6505.1.A1_at | 2,1 | 0,0146 |
| SH3 domain binding glutamic acid-rich protein like 3 | SH3BGRL3 | CfaAffx.19489.1.S1_s_at | 2,1 | 0,0313 |
| cell division cycle 42 (GTP binding protein, 25kDa) /// similar to cell division cycle 42 | CDC42 | CfaAffx.12195.1.S1_s_at | 2,1 | 0,0076 |
| mitogen-activated protein kinase 14 | MAPK14 | Cfa.1239.1.S1_s_at | 2,1 | 0,0046 |
| cathelicidin antimicrobial peptide | CAMP | Cfa.4552.1.S1_s_at | 2,1 | 0,0224 |
| tyrosylprotein sulfotransferase 2 | TPST2 | CfaAffx.18284.1.S1_at | 2,1 | 0,0201 |
| RCSD domain containing 1 | RCSD1 | CfaAffx.23665.1.S1_at | 2,1 | 0,0129 |
| nuclear factor, interleukin 3 regulated | NFIL3 | CfaAffx.4141.1.S1_s_at | 2,1 | 0,0076 |
| similar to down-regulated by Ctnnb1, a | LOC475254 | CfaAffx.5005.1.S1_at | 2,1 | 0,0095 |
| leptin receptor overlapping transcript-like 1 | LEPROTL1 | Cfa.9151.1.A1_s_at | 2,1 | 0,0085 |
| mediator of cell motility 1 | MEMO1 | CfaAffx.9510.1.S1_s_at | 2,1 | 0,0160 |
| KIT ligand | KITLG | CfaAffx.10131.1.S1_s_at | 2,1 | 0,0391 |
| signal transducer and activator of transcription 5B | STAT5B | CfaAffx.23894.1.S1_at | 2,1 | 0,0167 |
| integrin, alpha X (complement component 3 receptor 4 subunit) | ITGAX | CfaAffx.25820.1.S1_at | 2,1 | 0,0155 |
| similar to phosphoglycerate kinase 1 /// phosphoglycerate kinase 1 | LOC486305 | Cfa.1363.1.A1_x_at | 2,1 | 0,0317 |
| similar to proline-serine-threonine phosphatase interacting protein 1 | LOC611757 | CfaAffx.27686.1.S1_at | 2,1 | 0,0025 |
| aldolase A, fructose-bisphosphate | ALDOA | CfaAffx.26135.1.S1_s_at | 2,1 | 0,0402 |
| lysosomal-associated membrane protein 2 | LAMP2 | CfaAffx.28308.1.S1_s_at | 2,1 | 0,0128 |
| calreticulin | CALR | Cfa.4277.1.A1_s_at | 2,1 | 0,0209 |
| heme oxygenase (decycling) 1 | HMOX1 | Cfa.4568.1.S1_s_at | 2,1 | 0,0026 |
| similar to Tyrosine-protein phosphatase non-receptor type substrate 1 precursor (SHP substrate-1) | SIRPA | Cfa.14744.1.S1_s_at | 2,1 | 0,0388 |
| G1 to S phase transition 1 | GSPT1 | Cfa.20162.1.S1_s_at | 2,1 | 0,0066 |
| similar to neurobeachin-like 1 | LOC488482 | CfaAffx.19773.1.S1_s_at | 2,1 | 0,0367 |
| transmembrane protein 167A | TMEM167A | Cfa.10458.1.S1_at | 2,1 | 0,0366 |
| RAP2C, member of RAS oncogene family | RAP2C | CfaAffx.28755.1.S1_at | 2,1 | 0,0078 |
| interleukin 13 receptor, alpha 1 | IL13RA1 | Cfa.866.1.S1_at | 2,1 | 0,0235 |
| NECAP endocytosis associated 2 | NECAP2 | Cfa.10675.1.S1_at | 2,1 | 0,0085 |
| multiple C2 domains, transmembrane 1 | MCTP1 | CfaAffx.12817.1.S1_s_at | 2,1 | 0,0202 |
| 4-hydroxyphenylpyruvate dioxygenase | HPD | CfaAffx.12947.1.S1_s_at | 2,1 | 0,0390 |
| capping protein (actin filament) muscle Z-line, alpha 1 | CAPZA1 | CfaAffx.20828.1.S1_s_at | 2,1 | 0,0285 |
| glutaredoxin (thioltransferase) | GLRX | Cfa.21025.1.S1_at | 2,1 | 0,0463 |
| similar to ninjurin 1 | LOC610587 | CfaAffx.4282.1.S1_at | 2,1 | 0,0065 |
| protein tyrosine phosphatase, non-receptor type 9 | PTPN9 | Cfa.15034.1.A1_at | 2,1 | 0,0052 |
| ROD1 regulator of differentiation 1 (S. pombe) | ROD1 | CfaAffx.5464.1.S1_at | 2,1 | 0,0321 |
| amyloid beta (A4) precursor-like protein 2 | APLP2 | CfaAffx.15873.1.S1_s_at | 2,1 | 0,0493 |
| mucosa associated lymphoid tissue lymphoma translocation gene 1 | MALT1 | Cfa.11840.1.A1_at | 2,1 | 0,0178 |
| actin, beta | ACTB | CfaAffx.23258.1.S1_x_at | 2,1 | 0,0139 |
| tescalcin | TESC | CfaAffx.14651.1.S1_at | 2,0 | 0,0454 |
| chondroitin sulfate synthase 1 | CHSY1 | CfaAffx.16537.1.S1_at | 2,0 | 0,0218 |
| p21 protein (Cdc42/Rac)-activated kinase 2 | PAK2 | CfaAffx.20252.1.S1_s_at | 2,0 | 0,0114 |
| reticulon 3 | RTN3 | Cfa.13648.1.A1_at | 2,0 | 0,0085 |
| similar to Type III iodothyronine deiodinase (Type-III 5deiodinase) (DIOIII) (Type 3 DI) (5DIII) | LOC612596 | CfaAffx.27457.1.S1_at | 2,0 | 0,0398 |
| similar to atlastin-like | LOC476044 | Cfa.21027.1.S1_s_at | 2,0 | 0,0282 |
| crystallin, beta B1 | CRYBB1 | CfaAffx.18332.1.S1_at | 2,0 | 0,0480 |
| purinergic receptor P2Y, G-protein coupled, 13 | P2RY13 | CfaAffx.13506.1.S1_at | 2,0 | 0,0295 |
| CD93 molecule | CD93 | CfaAffx.8676.1.S1_at | 2,0 | 0,0057 |
| mitogen-activated protein kinase kinase 1 | MAP2K1 | CfaAffx.26470.1.S1_s_at | 2,0 | 0,0067 |
| serine/threonine kinase 38 like | STK38L | CfaAffx.17409.1.S1_at | 2,0 | 0,0225 |
| solute carrier family 9 (sodium/hydrogen exchanger), member 6 | SLC9A6 | Cfa.13988.1.A1_at | 2,0 | 0,0044 |
| frizzled homolog 2 (Drosophila) | FZD2 | CfaAffx.21807.1.S1_at | 2,0 | 0,0456 |
| transmembrane 6 superfamily member 1 | TM6SF1 | CfaAffx.20430.1.S1_at | 2,0 | 0,0200 |
| calponin 2 | CNN2 | CfaAffx.30039.1.S1_at | 2,0 | 0,0421 |
| protein phosphatase 2, regulatory subunit B', gamma isoform | PPP2R5C | Cfa.10495.1.A1_at | 2,0 | 0,0192 |
| interleukin 17F | IL17F | CfaAffx.4163.1.S1_at | 2,0 | 0,0121 |
| myotrophin | MTPN | CfaAffx.5970.1.S1_s_at | 2,0 | 0,0019 |
| c-src tyrosine kinase | CSK | CfaAffx.27473.1.S1_s_at | 2,0 | 0,0043 |
| similar to Reticulon protein 3 (Neuroendocrine-specific protein-like 2) (NSP-like protein II) (NSPLII) | RTN3 | CfaAffx.2236.1.S1_s_at | 2,0 | 0,0085 |
| similar to cytochrome b5 outer mitochondrial membrane precursor | LOC610942 | Cfa.15386.1.A1_at | 2,0 | 0,0193 |
| YKT6 v-SNARE homolog (S. cerevisiae) | YKT6 | Cfa.10602.1.A1_at | 2,0 | 0,0106 |
| ribosomal protein L22-like 1 | RPL22L1 | CfaAffx.23004.1.S1_s_at | 2,0 | 0,0385 |
| transmembrane protein 71 | TMEM71 | CfaAffx.2549.1.S1_at | 2,0 | 0,0082 |
| centrosomal protein 170kDa | CEP170 | CfaAffx.24199.1.S1_s_at | 2,0 | 0,0317 |
| cOR9S10 olfactory receptor family 9 subfamily S-like | cOR9S10 | CfaAffx.631.1.S1_x_at | 2,0 | 0,0313 |
| EGF-like repeats and discoidin I-like domains 3 | EDIL3 | CfaAffx.13441.1.S1_s_at | 2,0 | 0,0184 |
| OAF homolog (Drosophila) | OAF | Cfa.10215.1.A1_at | 2,0 | 0,0127 |
| maltase-glucoamylase (alpha-glucosidase) | MGAM | CfaAffx.6714.1.S1_at | 2,0 | 0,0077 |
| myeloid cell leukemia sequence 1 (BCL2-related) | MCL1 | Cfa.34.1.S1_s_at | 2,0 | 0,0099 |
| transgelin 2 | TAGLN2 | Cfa.19077.1.S1_s_at | 2,0 | 0,0247 |
| eukaryotic translation initiation factor 5A2 | EIF5A2 | CfaAffx.23010.1.S1_s_at | 2,0 | 0,0307 |
| elongation factor RNA polymerase II | ELL | CfaAffx.22762.1.S1_s_at | 2,0 | 0,0077 |
| DENN/MADD domain containing 2A | DENND2A | CfaAffx.6850.1.S1_s_at | 2,0 | 0,0106 |
