# Supplementary material for: Canine Uterine Bacterial Infection Induces Upregulation of Proteolysis-Related Genes and Downregulation of Homeobox and Zinc Finger Factors
Source: PLoS One. 2009 Nov 26;4(11):e8039. doi: 10.1371/journal.pone.0008039 (PMC2777310; doi:10.1371/journal.pone.0008039)
Supplement: Table S4 — All genes showing significant (adj p<0.05) and more than 2-fold downregulation, in comparison with healthy controls, in uteri from animals diagnosed with uterine bacterial infection (0.88 MB DOC) [file pone.0008039.s004.doc]

Table S4. All genes showing significant (adj p<0.05) and more than 2-fold downregulation, in comparison with healthy controls, in uteri from animals diagnosed with uterine bacterial infection

| **Gene Title** | **Gene**  **Symbol** | **ID** | **fold**  **change** | **adj.P.**  **Val** |
| --- | --- | --- | --- | --- |
| sulfotransferase | SULT1D1 | Cfa.3502.1.S1_at | -30,6 | 0,018 |
| EPH receptor A7 | EPHA7 | CfaAffx.6057.1.S1_s_at | -26,0 | 0,003 |
| transcription factor CP2-like 1 | TFCP2L1 | Cfa.15666.1.A1_at | -15,2 | 0,002 |
| synuclein, alpha interacting protein | SNCAIP | Cfa.13718.1.S1_s_at | -10,9 | 0,001 |
| fasciculation and elongation protein zeta 1 (zygin I) | FEZ1 | Cfa.18202.2.S1_a_at | -10,7 | 0,042 |
| similar to phosphatidylethanolamine-binding protein 4 | LOC608950 | Cfa.17117.1.S1_at | -10,6 | 0,004 |
| similar to esophageal cancer related gene 4 protein | LOC611190 | CfaAffx.4062.1.S1_at | -10,5 | 0,001 |
| trefoil factor 2 | TFF2 | Cfa.201.1.S1_at | -9,2 | 0,048 |
| regulator of G-protein signaling 22 | RGS22 | CfaAffx.1736.1.S1_at | -8,9 | 0,002 |
| epoxide hydrolase 2, cytoplasmic | EPHX2 | Cfa.574.1.A1_at | -8,3 | 0,001 |
| Norrie disease (pseudoglioma) | NDP | Cfa.4725.1.S1_at | -8,3 | 0,007 |
| ankyrin 3, node of Ranvier (ankyrin G) | ANK3 | CfaAffx.19834.1.S1_s_at | -8,1 | 0,003 |
| rhophilin, Rho GTPase binding protein 2 | RHPN2 | Cfa.15.1.S1_at | -8,0 | 0,006 |
| hydroxypyruvate isomerase homolog (E. coli) | HYI | CfaAffx.8731.1.S1_s_at | -7,9 | 0,004 |
| ectonucleotide pyrophosphatase/phosphodiesterase 6 | ENPP6 | CfaAffx.12520.1.S1_at | -7,9 | 0,012 |
| msh homeobox 2 | MSX2 | Cfa.3529.1.S1_at | -7,9 | 0,014 |
| forkhead box A2 | FOXA2 | Cfa.13382.1.A1_at | -7,8 | 0,007 |
| similar to Homeobox protein DLX-6 | LOC482312 | CfaAffx.4169.1.S1_at | -7,5 | 0,001 |
| distal-less homeobox 5 | DLX5 | CfaAffx.4173.1.S1_at | -7,4 | 0,006 |
| solute carrier family 30 (zinc transporter), member 2 | SLC30A2 | Cfa.5561.1.A1_at | -7,4 | 0,014 |
| lymphoid enhancer-binding factor 1 | LEF1 | CfaAffx.17535.1.S1_s_at | -7,4 | 0,019 |
| F-box and WD repeat domain containing 10 | FBXW10 | Cfa.11549.1.A1_at | -7,3 | 0,026 |
| cholecystokinin | CCK | CfaAffx.8825.1.S1_s_at | -7,1 | 0,027 |
| aldehyde dehydrogenase 1 family, member A1 | ALDH1A1 | Cfa.1715.1.S1_at | -7,1 | 0,018 |
| epoxide hydrolase 2, cytoplasmic | EPHX2 | CfaAffx.13394.1.S1_s_at | -7,0 | 0,001 |
| glutamate-cysteine ligase, catalytic subunit | GCLC | CfaAffx.4309.1.S1_s_at | -7,0 | 0,014 |
| phosphatidic acid phosphatase type 2 domain containing 1A | PPAPDC1A | Cfa.5652.1.A1_at | -6,9 | 0,023 |
| similar to RIKEN cDNA 5133401N09 | LOC484150 | CfaAffx.3054.1.S1_at | -6,6 | 0,042 |
| protein phosphatase 1, regulatory (inhibitor) subunit 1B | PPP1R1B | Cfa.20636.1.S1_at | -6,6 | 0,027 |
| similar to Epithelial-cadherin precursor (E-cadherin) | LOC489647 | CfaAffx.30291.1.S1_at | -6,6 | 0,009 |
| DEP domain containing 7 | DEPDC7 | CfaAffx.11821.1.S1_at | -6,6 | 0,044 |
| similar to EGFR-coamplified and overexpressed protein | LOC608562 | Cfa.20305.1.S1_at | -6,6 | 0,009 |
| betaine-homocysteine methyltransferase | BHMT | Cfa.11111.1.A1_at | -6,5 | 0,002 |
| N-acetylated alpha-linked acidic dipeptidase 2 | NAALAD2 | CfaAffx.7397.1.S1_s_at | -6,4 | 0,038 |
| similar to dachshund homolog 1 isoform a | LOC485489 | CfaAffx.8384.1.S1_at | -6,4 | 0,016 |
| carboxylesterase 2 (intestine, liver) | CES2 | Cfa.19114.1.S1_at | -6,3 | 0,005 |
| msh homeobox 1 | MSX1 | CfaAffx.24056.1.S1_at | -6,3 | 0,010 |
| spermatid perinuclear RNA binding protein | STRBP | Cfa.19369.1.S1_at | -6,2 | 0,002 |
| EF-hand domain (C-terminal) containing 2 | EFHC2 | Cfa.9584.1.A1_s_at | -6,1 | 0,001 |
| cystathionase (cystathionine gamma-lyase) | CTH | Cfa.359.1.S1_at | -6,1 | 0,005 |
| SH3 domain binding glutamic acid-rich protein like 2 | SH3BGRL2 | CfaAffx.5151.1.S1_s_at | -6,0 | 0,033 |
| glutamate receptor interacting protein 1 | GRIP1 | Cfa.8622.1.A1_s_at | -5,9 | 0,006 |
| CKLF-like MARVEL transmembrane domain containing 8 | CMTM8 | Cfa.8530.1.A1_s_at | -5,7 | 0,017 |
| tumor-associated calcium signal transducer 1 | TACSTD1 | CfaAffx.4844.1.S1_at | -5,7 | 0,004 |
| similar to myosin 18A isoform b | LOC475308 | Cfa.1186.1.A1_s_at | -5,6 | 0,003 |
| similar to ankyrin repeat domain 26 | LOC610965 | CfaAffx.12169.1.S1_at | -5,6 | 0,009 |
| WNT inhibitory factor 1 | WIF1 | Cfa.4881.1.A1_at | -5,5 | 0,018 |
| similar to Arg/Abl-interacting protein 2 isoform 1 | LOC482906 | CfaAffx.12091.1.S1_s_at | -5,4 | 0,012 |
| RAS guanyl releasing protein 1 (calcium and DAG-regulated) | RASGRP1 | Cfa.7809.1.A1_at | -5,4 | 0,040 |
| similar to membrane-associated guanylate kinase-related (MAGI-3) | LOC479761 | CfaAffx.24689.1.S1_at | -5,4 | 0,008 |
| TSC22 domain family, member 1 | TSC22D1 | CfaAffx.7831.1.S1_s_at | -5,4 | 0,007 |
| similar to odd Oz/ten-m homolog 4 | LOC485166 | CfaAffx.8044.1.S1_s_at | -5,4 | 0,004 |
| SLIT and NTRK-like family, member 6 | SLITRK6 | CfaAffx.8878.1.S1_at | -5,3 | 0,009 |
| tetratricopeptide repeat domain 23-like | TTC23L | CfaAffx.28729.1.S1_s_at | -5,3 | 0,017 |
| homeobox A6 | HOXA6 | CfaAffx.5356.1.S1_at | -5,3 | 0,001 |
| N-acetylated alpha-linked acidic dipeptidase-like 2 | NAALADL2 | CfaAffx.23823.1.S1_at | -5,3 | 0,001 |
| zinc finger, CCHC domain containing 12 | ZCCHC12 | Cfa.6433.1.A1_at | -5,2 | 0,022 |
| metallophosphoesterase domain containing 2 | MPPED2 | CfaAffx.12168.1.S1_at | -5,2 | 0,001 |
| RAN binding protein 17 | RANBP17 | Cfa.6781.1.A1_at | -5,2 | 0,016 |
| zinc finger protein 704 | ZNF704 | Cfa.17854.1.S1_at | -5,2 | 0,027 |
| microfibrillar-associated protein 3-like | MFAP3L | CfaAffx.12367.1.S1_at | -5,1 | 0,005 |
| homeobox A4 | HOXA4 | Cfa.14028.1.A1_at | -5,1 | 0,003 |
| homeobox A5 | HOXA5 | CfaAffx.5350.1.S1_at | -5,1 | 0,001 |
| catenin (cadherin-associated protein), delta 2 | CTNND2 | CfaAffx.15659.1.S1_at | -5,1 | 0,011 |
| MAM domain containing 2 | MAMDC2 | CfaAffx.3753.1.S1_at | -5,0 | 0,014 |
| pleiotrophin | PTN | CfaAffx.5983.1.S1_s_at | -4,9 | 0,007 |
| wingless-type MMTV integration site family, member 5B | WNT5B | CfaAffx.24413.1.S1_at | -4,9 | 0,008 |
| secretogranin V (7B2 protein) | SCG5 | Cfa.10794.1.A1_at | -4,9 | 0,002 |
| cold inducible RNA binding protein | CIRBP | Cfa.4292.2.A1_a_at | -4,8 | 0,009 |
| microtubule-associated protein 7 | MAP7 | Cfa.1404.1.A1_at | -4,8 | 0,040 |
| brain-specific angiogenesis inhibitor 3 | BAI3 | Cfa.11087.1.A1_s_at | -4,8 | 0,003 |
| erythrocyte membrane protein band 4.1 like 4B | EPB41L4B | Cfa.13723.1.A1_at | -4,8 | 0,011 |
| similar to tigger transposable element derived 4 | LOC475466 | Cfa.19693.1.S1_s_at | -4,7 | 0,010 |
| formin homology 2 domain containing 3 | FHOD3 | Cfa.9772.1.A1_at | -4,7 | 0,027 |
| cadherin 18, type 2 | CDH18 | CfaAffx.29091.1.S1_at | -4,7 | 0,001 |
| kallikrein-related peptidase 4 | KLK4 | Cfa.12289.1.A1_at | -4,6 | 0,024 |
| zinc finger protein 618 | ZNF618 | CfaAffx.5854.1.S1_s_at | -4,5 | 0,004 |
| protein tyrosine phosphatase, receptor type, F | PTPRF | CfaAffx.8627.1.S1_s_at | -4,5 | 0,010 |
| calcium/calmodulin-dependent protein kinase ID | CAMK1D | Cfa.4396.1.A1_at | -4,5 | 0,003 |
| pseudouridylate synthase 7 homolog (S. cerevisiae)-like | PUS7L | Cfa.2715.1.S1_s_at | -4,5 | 0,020 |
| UDP-N-acetyl-alpha-D-galactosamine:polypeptide N-acetylgalactosaminyltransferase 13 (GalNAc-T13) | GALNT13 | Cfa.13327.1.A1_s_at | -4,5 | 0,001 |
| IQ motif containing GTPase activating protein 2 | IQGAP2 | CfaAffx.14655.1.S1_s_at | -4,5 | 0,006 |
| myosin VB | MYO5B | Cfa.15484.1.A1_at | -4,4 | 0,047 |
| leucine rich repeat containing 3B | LRRC3B | CfaAffx.9449.1.S1_at | -4,4 | 0,012 |
| transcription elongation factor A (SII)-like 3 | TCEAL3 | Cfa.17956.1.S1_x_at | -4,4 | 0,012 |
| zinc finger, matrin type 1 | ZMAT1 | CfaAffx.27138.1.S1_at | -4,4 | 0,019 |
| protein tyrosine phosphatase, non-receptor type 3 | PTPN3 | Cfa.10873.1.A1_at | -4,4 | 0,005 |
| adenylate cyclase 8 (brain) | ADCY8 | CfaAffx.2517.1.S1_at | -4,4 | 0,019 |
| neurotrophic tyrosine kinase, receptor, type 3 | NTRK3 | Cfa.2717.1.A1_a_at | -4,4 | 0,008 |
| similar to NPD014 protein isoform 2 | LOC478182 | CfaAffx.19970.1.S1_at | -4,3 | 0,016 |
| interferon regulatory factor 6 | IRF6 | Cfa.13948.1.A1_at | -4,3 | 0,036 |
| nudix (nucleoside diphosphate linked moiety X)-type motif 2 | NUDT2 | CfaAffx.3791.1.S1_at | -4,3 | 0,004 |
| UPF3 regulator of nonsense transcripts homolog B (yeast) | UPF3B | CfaAffx.28188.1.S1_at | -4,3 | 0,025 |
| similar to Adapter-related protein complex 1 sigma 1B subunit (Sigma-adaptin 1B) | LOC611468 | CfaAffx.18992.1.S1_s_at | -4,3 | 0,008 |
| zinc finger protein 331 | ZNF331 | Cfa.1108.1.S1_s_at | -4,3 | 0,004 |
| beta-carotene oxygenase 2 | BCO2 | Cfa.2388.1.A1_at | -4,3 | 0,024 |
| ABI gene family, member 3 (NESH) binding protein | ABI3BP | Cfa.2104.1.S1_at | -4,2 | 0,022 |
| nei like 2 (E. coli) | NEIL2 | Cfa.2134.1.S1_at | -4,2 | 0,015 |
| enolase superfamily member 1 | ENOSF1 | Cfa.18734.1.S1_at | -4,2 | 0,007 |
| SATB homeobox 1 | SATB1 | CfaAffx.9726.1.S1_s_at | -4,1 | 0,005 |
| solute carrier family 44, member 3 | SLC44A3 | CfaAffx.30778.1.S1_s_at | -4,1 | 0,021 |
| fibrillin 2 | FBN2 | Cfa.19619.1.A1_at | -4,1 | 0,024 |
| similar to CG31751-PA, isoform A | LOC475133 | Cfa.2465.1.A1_at | -4,1 | 0,001 |
| suppression of tumorigenicity 7 | ST7 | CfaAffx.6058.1.S1_s_at | -4,1 | 0,003 |
| similar to Adseverin (Scinderin) | LOC475245 | Cfa.21551.1.S1_s_at | -4,1 | 0,010 |
| von Willebrand factor A domain containing 2 | VWA2 | CfaAffx.17618.1.S1_at | -4,1 | 0,007 |
| ecotropic viral integration site 1 | EVI1 | Cfa.13855.1.S1_s_at | -4,1 | 0,008 |
| zinc finger protein 606 | ZNF606 | CfaAffx.4453.1.S1_at | -4,0 | 0,000 |
| leucine-rich repeat-containing G protein-coupled receptor 4 | LGR4 | Cfa.14080.1.A1_at | -4,0 | 0,004 |
| coiled-coil domain containing 46 | CCDC46 | Cfa.18528.1.S1_at | -4,0 | 0,001 |
| CDP-diacylglycerol synthase (phosphatidate cytidylyltransferase) 1 | CDS1 | Cfa.10965.1.A1_at | -4,0 | 0,046 |
| similar to dynein, cytoplasmic, intermediate polypeptide 1 | LOC475236 | Cfa.10179.1.A1_at | -4,0 | 0,020 |
| cysteine conjugate-beta lyase, cytoplasmic | CCBL1 | CfaAffx.30657.1.S1_at | -4,0 | 0,011 |
| calcium channel, voltage-dependent, alpha 2/delta subunit 3 | CACNA2D3 | Cfa.11051.1.A1_at | -3,9 | 0,002 |
| similar to Small conductance calcium-activated potassium channel protein 2 (SK2) | LOC474640 | Cfa.5095.1.A1_s_at | -3,9 | 0,004 |
| zinc finger protein 594 | ZNF594 | CfaAffx.960.1.S1_at | -3,9 | 0,006 |
| glycine N-methyltransferase | GNMT | CfaAffx.3512.1.S1_s_at | -3,9 | 0,032 |
| roundabout, axon guidance receptor, homolog 1 (Drosophila) | ROBO1 | CfaAffx.12712.1.S1_s_at | -3,9 | 0,004 |
| arsenic (+3 oxidation state) methyltransferase | AS3MT | CfaAffx.16116.1.S1_at | -3,9 | 0,019 |
| odd-skipped related 2 (Drosophila) | OSR2 | CfaAffx.1696.1.S1_at | -3,8 | 0,026 |
| coxsackie virus and adenovirus receptor | CXADR | Cfa.942.1.S1_at | -3,8 | 0,040 |
| storkhead box 2 | STOX2 | CfaAffx.12530.1.S1_s_at | -3,8 | 0,012 |
| RAS-like, estrogen-regulated, growth inhibitor | RERG | CfaAffx.19898.1.S1_at | -3,8 | 0,015 |
| nuclear factor I/A | NFIA | CfaAffx.28793.1.S1_s_at | -3,8 | 0,007 |
| nuclear factor I/C (CCAAT-binding transcription factor) | NFIC | CfaAffx.29368.1.S1_s_at | -3,7 | 0,004 |
| NEDD4 binding protein 2 | N4BP2 | CfaAffx.24408.1.S1_at | -3,7 | 0,002 |
| similar to Rho GTPase-activating protein | LOC489283 | CfaAffx.16021.1.S1_at | -3,7 | 0,001 |
| tubby like protein 4 | TULP4 | Cfa.19872.1.A1_at | -3,7 | 0,018 |
| zinc finger protein 662 | ZNF662 | CfaAffx.8989.1.S1_s_at | -3,7 | 0,014 |
| myeloid zinc finger 1 | MZF1 | Cfa.11535.1.A1_at | -3,7 | 0,006 |
| similar to Gamma-taxilin (Lipopolysaccharide specific response protein 5) | LOC480853 | CfaAffx.19577.1.S1_s_at | -3,7 | 0,008 |
| similar to protocadherin 11 X-linked isoform a precursor | LOC608881 | CfaAffx.26710.1.S1_s_at | -3,7 | 0,003 |
| Regulation of nuclear pre-mRNA domain containing 1A | RPRD1A | CfaAffx.27347.1.S1_at | -3,6 | 0,001 |
| inositol hexaphosphate kinase 2 | IHPK2 | Cfa.18263.1.S1_s_at | -3,6 | 0,002 |
| CUG triplet repeat, RNA binding protein 2 | CUGBP2 | CfaAffx.8440.1.S1_s_at | -3,6 | 0,042 |
| TIA1 cytotoxic granule-associated RNA binding protein | TIA1 | Cfa.18602.2.S1_s_at | -3,6 | 0,019 |
| protein phosphatase 1J (PP2C domain containing) | PPM1J | Cfa.12037.1.A1_at | -3,6 | 0,023 |
| homeobox A7 | HOXA7 | CfaAffx.5364.1.S1_at | -3,6 | 0,002 |
| tetraspanin 8 | TSPAN8 | Cfa.6237.1.S1_at | -3,6 | 0,030 |
| homeobox B5 | HOXB5 | CfaAffx.25767.1.S1_at | -3,6 | 0,001 |
| transmembrane protein with EGF-like and two follistatin-like domains 2 | TMEFF2 | CfaAffx.15844.1.S1_s_at | -3,5 | 0,037 |
| p21 protein (Cdc42/Rac)-activated kinase 1 | PAK1 | Cfa.3213.1.A1_at | -3,5 | 0,038 |
| RNA binding motif protein 26 | RBM26 | Cfa.19484.1.S1_at | -3,5 | 0,000 |
| tumor necrosis factor, alpha-induced protein 8-like 3 | TNFAIP8L3 | Cfa.10601.2.S1_a_at | -3,5 | 0,019 |
| CDC-like kinase 4 | CLK4 | Cfa.3071.1.S1_at | -3,5 | 0,017 |
| NMDA receptor regulated 1-like | NARG1L | Cfa.19752.1.S1_s_at | -3,5 | 0,002 |
| B-cell CLL/lymphoma 9 | BCL9 | CfaAffx.17255.1.S1_at | -3,5 | 0,038 |
| wingless-type MMTV integration site family, member 11 | WNT11 | CfaAffx.8886.1.S1_at | -3,5 | 0,002 |
| leucine-rich, glioma inactivated 1 | LGI1 | Cfa.1413.1.A1_s_at | -3,5 | 0,008 |
| cyclin G2 | CCNG2 | CfaAffx.13734.1.S1_s_at | -3,4 | 0,009 |
| phosphoribosyl pyrophosphate synthetase-associated protein 1 | PRPSAP1 | Cfa.15699.1.A1_at | -3,4 | 0,010 |
| nuclear autoantigenic sperm protein (histone-binding) | NASP | Cfa.2896.1.S1_at | -3,4 | 0,003 |
| Ras-related GTP binding B | RRAGB | CfaAffx.25259.1.S1_s_at | -3,4 | 0,005 |
| similar to glutamic pyruvic transaminase 1, soluble | LOC609914 | Cfa.20911.2.S1_at | -3,4 | 0,006 |
| G protein-coupled receptor associated sorting protein 1 | GPRASP1 | CfaAffx.27192.1.S1_at | -3,4 | 0,019 |
| similar to CG9005-PA | LOC487557 | CfaAffx.24307.1.S1_at | -3,4 | 0,013 |
| similar to Protein FAM13C1 | LOC488991 | CfaAffx.19277.1.S1_at | -3,4 | 0,004 |
| similar to CG14853-PB, isoform B | LOC478793 | Cfa.20586.1.S1_at | -3,4 | 0,021 |
| solute carrier family 27 (fatty acid transporter), member 5 | SLC27A5 | Cfa.14551.1.A1_at | -3,3 | 0,036 |
| tetratricopeptide repeat domain 14 | TTC14 | Cfa.683.1.A1_at | -3,3 | 0,008 |
| homeobox B7 | HOXB7 | Cfa.15630.1.A1_at | -3,3 | 0,013 |
| RAB3A interacting protein (rabin3) | RAB3IP | Cfa.1634.2.A1_s_at | -3,3 | 0,014 |
| suppressor of cytokine signaling 6 | SOCS6 | Cfa.2103.1.A1_at | -3,3 | 0,037 |
| R-spondin 3 homolog (Xenopus laevis) | RSPO3 | Cfa.14613.1.A1_s_at | -3,3 | 0,029 |
| vascular endothelial zinc finger 1 | VEZF1 | Cfa.5843.1.A2_at | -3,3 | 0,006 |
| occludin | OCLN | Cfa.3606.1.S1_at | -3,3 | 0,033 |
| mutS homolog 5 (E. coli) | MSH5 | Cfa.5530.1.A1_at | -3,3 | 0,016 |
| transducin-like enhancer of split 1 (E(sp1) homolog, Drosophila) | TLE1 | Cfa.11290.1.A1_s_at | -3,3 | 0,004 |
| DEAQ box polypeptide 1 (RNA-dependent ATPase) | DQX1 | Cfa.8542.1.A1_at | -3,3 | 0,030 |
| lin-7 homolog A (C. elegans) | LIN7A | CfaAffx.9679.1.S1_at | -3,3 | 0,030 |
| cyclin G1 | CCNG1 | Cfa.872.1.A1_at | -3,3 | 0,034 |
| retinoblastoma binding protein 7 | RBBP7 | Cfa.7590.1.A1_at | -3,3 | 0,008 |
| neurobeachin | NBEA | Cfa.15635.1.A1_s_at | -3,2 | 0,012 |
| transcription elongation factor A (SII), 2 | TCEA2 | CfaAffx.23377.1.S1_s_at | -3,2 | 0,009 |
| ovo-like 2 (Drosophila) | OVOL2 | CfaAffx.9115.1.S1_at | -3,2 | 0,019 |
| WD repeat domain 60 | WDR60 | Cfa.12294.1.A1_at | -3,2 | 0,003 |
| similar to Protein C1orf43 | LOC480136 | Cfa.10125.4.A1_at | -3,2 | 0,009 |
| methionine sulfoxide reductase B2 | MSRB2 | Cfa.10136.2.S1_a_at | -3,2 | 0,015 |
| tubulin, gamma complex associated protein 4 | TUBGCP4 | Cfa.11058.1.A1_at | -3,2 | 0,006 |
| mitochondrial intermediate peptidase | MIPEP | Cfa.12350.1.A1_at | -3,2 | 0,004 |
| coiled-coil domain containing 18 | CCDC18 | CfaAffx.30843.1.S1_s_at | -3,2 | 0,024 |
| similar to MYST histone acetyltransferase monocytic leukemia 4 | LOC479254 | CfaAffx.23612.1.S1_s_at | -3,2 | 0,022 |
| zinc finger protein 208 | ZNF208 | CfaAffx.4444.1.S1_at | -3,2 | 0,012 |
| transmembrane protein 55A | TMEM55A | CfaAffx.14248.1.S1_s_at | -3,1 | 0,003 |
| adenosine deaminase-like | ADAL | Cfa.15786.1.A1_at | -3,1 | 0,021 |
| ATPase, class V, type 10A | ATP10A | Cfa.15944.1.A1_at | -3,1 | 0,014 |
| collagen and calcium binding EGF domains 1 | CCBE1 | CfaAffx.1108.1.S1_s_at | -3,1 | 0,007 |
| ATPase family, AAA domain containing 4 | ATAD4 | CfaAffx.25710.1.S1_at | -3,1 | 0,042 |
| threonyl-tRNA synthetase-like 2 | TARSL2 | CfaAffx.16400.1.S1_s_at | -3,1 | 0,003 |
| resistance to inhibitors of cholinesterase 8 homolog B (C. elegans) | RIC8B | Cfa.2520.1.A1_at | -3,1 | 0,001 |
| InaD-like (Drosophila) | INADL | CfaAffx.28758.1.S1_s_at | -3,1 | 0,014 |
| Ras association (RalGDS/AF-6) domain family member 6 | RASSF6 | CfaAffx.5492.1.S1_s_at | -3,1 | 0,028 |
| NOL1/NOP2/Sun domain family, member 7 | NSUN7 | Cfa.20111.1.S1_s_at | -3,1 | 0,032 |
| RNA binding motif protein 5 | RBM5 | CfaAffx.16999.1.S1_s_at | -3,1 | 0,007 |
| coiled-coil domain containing 146 | CCDC146 | Cfa.11687.1.A1_at | -3,1 | 0,020 |
| armadillo repeat containing, X-linked 5 | ARMCX5 | Cfa.2626.1.A1_a_at | -3,1 | 0,003 |
| basonuclin 2 | BNC2 | CfaAffx.3241.1.S1_s_at | -3,1 | 0,033 |
| similar to family with sequence similarity 36, member A | LOC480099 | Cfa.14430.1.S1_at | -3,1 | 0,001 |
| transmembrane protein 67 | TMEM67 | Cfa.1725.1.S1_at | -3,1 | 0,004 |
| similar to Sh3 domain YSC-like 1 | LOC606804 | Cfa.3339.1.A1_at | -3,1 | 0,012 |
| zinc finger protein 182 | ZNF182 | CfaAffx.23416.1.S1_at | -3,1 | 0,007 |
| myotubularin related protein 7 | MTMR7 | CfaAffx.11263.1.S1_at | -3,1 | 0,012 |
| ectonucleoside triphosphate diphosphohydrolase 5 | ENTPD5 | Cfa.20908.1.A1_at | -3,0 | 0,004 |
| RIO kinase 2 (yeast) | RIOK2 | Cfa.18727.1.S1_at | -3,0 | 0,023 |
| similar to chromosome 9 open reading frame 61 | LOC609015 | Cfa.17597.1.S1_at | -3,0 | 0,020 |
| D-aspartate oxidase | DDO | Cfa.12618.1.A1_at | -3,0 | 0,021 |
| similar to TAR DNA binding protein isoform 4 | LOC478234 | CfaAffx.25618.1.S1_at | -3,0 | 0,004 |
| palmdelphin | PALMD | Cfa.17587.1.S1_at | -3,0 | 0,013 |
| programmed cell death 4 (neoplastic transformation inhibitor) | PDCD4 | Cfa.19830.1.S1_s_at | -3,0 | 0,033 |
| actin-like 7A | ACTL7A | Cfa.7390.1.A1_s_at | -3,0 | 0,021 |
| estrogen receptor 1 | ESR1 | Cfa.3504.1.S1_s_at | -3,0 | 0,023 |
| myeloid/lymphoid or mixed-lineage leukemia 3 | MLL3 | CfaAffx.8351.1.S1_s_at | -3,0 | 0,034 |
| phospholipase C-like 2 | PLCL2 | CfaAffx.9752.1.S1_at | -3,0 | 0,037 |
| StAR-related lipid transfer (START) domain containing 6 | STARD6 | CfaAffx.1194.1.S1_at | -3,0 | 0,012 |
| zinc finger protein 37A | ZNF37A | CfaAffx.15623.1.S1_s_at | -3,0 | 0,006 |
| galactose mutarotase (aldose 1-epimerase) | GALM | Cfa.18790.1.S1_s_at | -3,0 | 0,008 |
| ATP-binding cassette, sub-family G (WHITE), member 2 | ABCG2 | CfaAffx.15156.1.S1_at | -3,0 | 0,018 |
| similar to TATA-binding protein-like factor-interacting protein isoform 2 | LOC612738 | CfaAffx.9498.1.S1_at | -3,0 | 0,002 |
| target of myb1 (chicken)-like 1 | TOM1L1 | Cfa.9874.1.S1_at | -3,0 | 0,046 |
| BRCA1 associated RING domain 1 | BARD1 | Cfa.8203.1.A1_at | -3,0 | 0,011 |
| RNA binding motif protein 35B | RBM35B | Cfa.7717.1.A1_at | -3,0 | 0,016 |
| similar to Nuclear factor 1 B-type (Nuclear factor 1/B) (NF1-B) | LOC474709 | CfaAffx.3189.1.S1_s_at | -3,0 | 0,019 |
| nucleoporin 133kDa | NUP133 | Cfa.315.1.A1_at | -3,0 | 0,007 |
| microtubule associated serine/threonine kinase family member 4 | MAST4 | CfaAffx.12239.1.S1_s_at | -3,0 | 0,029 |
| 3-hydroxymethyl-3-methylglutaryl-Coenzyme A lyase-like 1 | HMGCLL1 | Cfa.18674.1.S1_s_at | -3,0 | 0,026 |
| similar to chromosome 1 open reading frame 21 | LOC610661 | CfaAffx.20797.1.S1_at | -3,0 | 0,011 |
| pleckstrin homology domain containing, family A, member 1 | PLEKHA1 | CfaAffx.19448.1.S1_s_at | -3,0 | 0,017 |
| integrin, alpha 6 | ITGA6 | Cfa.2219.1.A1_at | -2,9 | 0,002 |
| nipsnap homolog 1 (C. elegans) | NIPSNAP1 | Cfa.11282.1.A1_at | -2,9 | 0,002 |
| splicing factor, arginine/serine-rich 18 | SFRS18 | Cfa.15354.1.S1_s_at | -2,9 | 0,005 |
| parathyroid hormone receptor 1 | PTHR1 | Cfa.3643.1.S1_s_at | -2,9 | 0,044 |
| dystonin | DST | Cfa.15054.1.A1_s_at | -2,9 | 0,032 |
| activated leukocyte cell adhesion molecule | ALCAM | Cfa.3742.1.S1_s_at | -2,9 | 0,029 |
| SEC22 vesicle trafficking protein homolog C (S. cerevisiae) | SEC22C | Cfa.2909.1.A1_at | -2,9 | 0,006 |
| heterogeneous nuclear ribonucleoprotein A2/B1 | HNRNPA2B1 | Cfa.10247.2.S1_at | -2,9 | 0,016 |
| proline rich 15 | PRR15 | Cfa.12462.1.A1_at | -2,9 | 0,040 |
| protein tyrosine phosphatase domain containing 1 | PTPDC1 | Cfa.12384.1.A1_at | -2,9 | 0,042 |
| zinc finger protein 643 | ZNF643 | CfaAffx.5393.1.S1_s_at | -2,9 | 0,007 |
| zinc finger, MYM-type 1 | ZMYM1 | Cfa.20320.1.S1_at | -2,9 | 0,004 |
| zinc finger protein 99 | ZNF99 | Cfa.292.2.S1_s_at | -2,9 | 0,018 |
| calmodulin regulated spectrin-associated protein 1 | CAMSAP1 | Cfa.11486.1.A1_at | -2,9 | 0,013 |
| kelch domain containing 1 | KLHDC1 | CfaAffx.21923.1.S1_at | -2,9 | 0,019 |
| similar to Kinesin-like protein KIFC2 | LOC609880 | Cfa.8094.1.A1_at | -2,9 | 0,005 |
| protein kinase D1 | PRKD1 | CfaAffx.19461.1.S1_at | -2,9 | 0,006 |
| membrane protein, palmitoylated 6 (MAGUK p55 subfamily member 6) | MPP6 | Cfa.19439.1.S1_s_at | -2,9 | 0,005 |
| clathrin, heavy chain-like 1 | CLTCL1 | Cfa.1340.1.S1_at | -2,9 | 0,013 |
| centrosomal protein 290kDa | CEP290 | CfaAffx.10052.1.S1_s_at | -2,8 | 0,002 |
| nuclear receptor co-repressor 1 | NCOR1 | CfaAffx.27681.1.S1_at | -2,8 | 0,010 |
| SRY (sex determining region Y)-box 5 | SOX5 | CfaAffx.18082.1.S1_s_at | -2,8 | 0,013 |
| latrophilin 3 | LPHN3 | CfaAffx.4423.1.S1_s_at | -2,8 | 0,007 |
| similar to Zinc finger protein 432 | LOC484338 | CfaAffx.5099.1.S1_at | -2,8 | 0,004 |
| minichromosome maintenance complex component 8 | MCM8 | Cfa.6086.1.A1_s_at | -2,8 | 0,016 |
| transmembrane protein 126A | TMEM126A | Cfa.19473.1.S1_at | -2,8 | 0,002 |
| nemo-like kinase | NLK | Cfa.2815.1.S1_at | -2,8 | 0,007 |
| syntaxin 16 | STX16 | Cfa.10962.1.A1_at | -2,8 | 0,007 |
| acid phosphatase 6, lysophosphatidic | ACP6 | Cfa.14387.1.A1_s_at | -2,8 | 0,003 |
| similar to Rho-guanine nucleotide exchange factor (RhoGEF) (RIP2) | LOC487245 | CfaAffx.13141.1.S1_at | -2,8 | 0,018 |
| LEM domain containing 3 | LEMD3 | Cfa.2925.1.A1_at | -2,8 | 0,002 |
| homeobox A2 | HOXA2 | CfaAffx.5335.1.S1_s_at | -2,8 | 0,012 |
| acyl-Coenzyme A binding domain containing 7 | ACBD7 | CfaAffx.7953.1.S1_at | -2,8 | 0,027 |
| similar to catenin, alpha-like 1 | LOC610662 | Cfa.1402.1.S1_at | -2,8 | 0,009 |
| myosin, light chain 6B, alkali, smooth muscle and non-muscle | MYL6B | Cfa.16968.1.S1_s_at | -2,8 | 0,004 |
| nuclear factor I/X (CCAAT-binding transcription factor) | NFIX | CfaAffx.26064.1.S1_s_at | -2,8 | 0,044 |
| similar to Alcohol dehydrogenase class III chi chain | LOC474946 | CfaAffx.20142.1.S1_s_at | -2,8 | 0,027 |
| similar to amyotrophic lateral sclerosis 2 (juvenile) chromosome region, candidate 4 | LOC488476 | CfaAffx.19118.1.S1_at | -2,8 | 0,009 |
| syntaxin 18 | STX18 | CfaAffx.24052.1.S1_s_at | -2,8 | 0,037 |
| sperm associated antigen 9 | SPAG9 | CfaAffx.26465.1.S1_s_at | -2,8 | 0,011 |
| DEAH (Asp-Glu-Ala-His) box polypeptide 9 | DHX9 | Cfa.14207.1.A1_at | -2,8 | 0,020 |
| zinc finger protein 583 | ZNF583 | CfaAffx.4594.1.S1_s_at | -2,8 | 0,032 |
| sperm specific antigen 2 | SSFA2 | CfaAffx.21963.1.S1_at | -2,8 | 0,018 |
| HIRA interacting protein 3 | HIRIP3 | Cfa.10826.1.A1_at | -2,8 | 0,008 |
| rotatin | RTTN | CfaAffx.1027.1.S1_at | -2,8 | 0,012 |
| amyotrophic lateral sclerosis 2 (juvenile) | ALS2 | CfaAffx.19197.1.S1_at | -2,7 | 0,043 |
| PRP4 pre-mRNA processing factor 4 homolog B (yeast) | PRPF4B | Cfa.1509.1.A1_s_at | -2,7 | 0,003 |
| splicing factor, arginine/serine-rich 14 | SFRS14 | CfaAffx.16723.1.S1_s_at | -2,7 | 0,011 |
| ubiquitin specific peptidase 13 (isopeptidase T-3) | USP13 | Cfa.15947.1.A1_s_at | -2,7 | 0,003 |
| homeobox containing 1 | HMBOX1 | CfaAffx.13080.1.S1_s_at | -2,7 | 0,007 |
| outer dense fiber of sperm tails 2-like | ODF2L | CfaAffx.31038.1.S1_s_at | -2,7 | 0,004 |
| TAF7 RNA polymerase II, TATA box binding protein (TBP)-associated factor, 55kDa | TAF7 | CfaAffx.974.1.S1_at | -2,7 | 0,018 |
| biphenyl hydrolase-like (serine hydrolase; breast epithelial mucin-associated antigen) | BPHL | CfaAffx.14710.1.S1_at | -2,7 | 0,036 |
| microphthalmia-associated transcription factor | MITF | Cfa.2682.2.S1_at | -2,7 | 0,013 |
| chromodomain helicase DNA binding protein 6 | CHD6 | CfaAffx.14589.1.S1_at | -2,7 | 0,014 |
| RNA binding motif protein, X-linked | RBMX | Cfa.1583.1.S1_at | -2,7 | 0,004 |
| congenital dyserythropoietic anemia, type I | CDAN1 | Cfa.15834.1.S1_at | -2,7 | 0,003 |
| zinc finger protein 30 | ZNF30 | CfaAffx.5988.1.S1_at | -2,7 | 0,003 |
| POU class 2 homeobox 1 | POU2F1 | CfaAffx.23833.1.S1_at | -2,7 | 0,003 |
| RNA binding motif protein 6 | RBM6 | Cfa.19772.1.S1_s_at | -2,7 | 0,007 |
| eukaryotic translation initiation factor 2B, subunit 4 delta, 67kDa | EIF2B4 | Cfa.2488.1.A1_at | -2,7 | 0,007 |
| zinc finger protein 665 | ZNF665 | CfaAffx.5129.1.S1_s_at | -2,7 | 0,017 |
| coiled-coil domain containing 141 | CCDC141 | CfaAffx.21768.1.S1_at | -2,7 | 0,011 |
| vestigial like 4 (Drosophila) | VGLL4 | CfaAffx.8432.1.S1_s_at | -2,7 | 0,015 |
| methylenetetrahydrofolate dehydrogenase (NADP+ dependent) 1-like | MTHFD1L | CfaAffx.1580.1.S1_s_at | -2,7 | 0,035 |
| carnitine palmitoyltransferase 1B (muscle) | CPT1B | Cfa.20603.1.S1_s_at | -2,7 | 0,011 |
| pterin-4 alpha-carbinolamine dehydratase/dimerization cofactor of hepatocyte nuclear factor 1 alpha | PCBD1 | CfaAffx.21771.1.S1_s_at | -2,7 | 0,007 |
| formin binding protein 1-like | FNBP1L | Cfa.2466.1.S1_at | -2,7 | 0,004 |
| epoxide hydrolase 1, microsomal (xenobiotic) | EPHX1 | CfaAffx.24797.1.S1_at | -2,7 | 0,009 |
| phosphatidylinositol glycan anchor biosynthesis, class P | PIGP | CfaAffx.15236.1.S1_at | -2,7 | 0,008 |
| SUMO/sentrin specific peptidase family member 8 | SENP8 | CfaAffx.27008.1.S1_at | -2,7 | 0,014 |
| similar to Galectin-5 (RL-18) | LOC481389 | CfaAffx.5708.1.S1_at | -2,7 | 0,024 |
| ATP-binding cassette, sub-family B (MDR/TAP), member 1 | ABCB1 | Cfa.19.1.S1_s_at | -2,7 | 0,025 |
| similar to CG14025-PB, isoform B | LOC477006 | Cfa.10510.1.A1_at | -2,7 | 0,007 |
| zinc finger and BTB domain containing 20 | ZBTB20 | Cfa.5314.1.A1_at | -2,7 | 0,020 |
| hook homolog 2 (Drosophila) | HOOK2 | Cfa.8017.1.A1_at | -2,7 | 0,012 |
| guanosine monophosphate reductase | GMPR | Cfa.18008.1.S1_s_at | -2,7 | 0,033 |
| Fas apoptotic inhibitory molecule | FAIM | Cfa.3417.1.S1_at | -2,7 | 0,005 |
| pinin, desmosome associated protein | PNN | CfaAffx.21428.1.S1_s_at | -2,7 | 0,003 |
| poly(A) binding protein interacting protein 1 | PAIP1 | Cfa.16793.1.S1_at | -2,7 | 0,003 |
| phenylalanyl-tRNA synthetase 2, mitochondrial | FARS2 | CfaAffx.14922.1.S1_s_at | -2,7 | 0,003 |
| phosphorylase kinase, alpha 2 (liver) | PHKA2 | Cfa.5653.1.A1_at | -2,7 | 0,011 |
| La ribonucleoprotein domain family, member 7 | LARP7 | Cfa.20816.2.S1_at | -2,7 | 0,018 |
| WW and C2 domain containing 1 | WWC1 | CfaAffx.26144.1.S1_at | -2,7 | 0,035 |
| oxysterol binding protein-like 9 | OSBPL9 | Cfa.18565.1.S1_at | -2,7 | 0,021 |
| mitogen-activated protein kinase kinase kinase kinase 3 | MAP4K3 | CfaAffx.10633.1.S1_s_at | -2,7 | 0,005 |
| period homolog 1 (Drosophila) | PER1 | CfaAffx.25981.1.S1_at | -2,7 | 0,010 |
| zinc finger protein 550 | ZNF550 | CfaAffx.4494.1.S1_s_at | -2,6 | 0,006 |
| Kruppel-like factor 11 | KLF11 | Cfa.20312.1.S1_at | -2,6 | 0,046 |
| retinitis pigmentosa GTPase regulator | RPGR | Cfa.3592.3.S1_s_at | -2,6 | 0,011 |
| CAP-GLY domain containing linker protein family, member 4 | CLIP4 | Cfa.2421.1.A1_at | -2,6 | 0,004 |
| MAM domain containing glycosylphosphatidylinositol anchor 2 | MDGA2 | CfaAffx.21857.1.S1_s_at | -2,6 | 0,022 |
| aminomethyltransferase | AMT | Cfa.11059.1.A1_at | -2,6 | 0,017 |
| far upstream element (FUSE) binding protein 1 | FUBP1 | Cfa.19489.1.S1_at | -2,6 | 0,001 |
| zinc finger protein 23 (KOX 16) | ZNF23 | CfaAffx.30900.1.S1_at | -2,6 | 0,006 |
| myeloid/lymphoid or mixed-lineage leukemia 2 | MLL2 | CfaAffx.13812.1.S1_s_at | -2,6 | 0,017 |
| islet cell autoantigen 1, 69kDa | ICA1 | CfaAffx.4267.1.S1_s_at | -2,6 | 0,019 |
| zinc finger, matrin type 4 | ZMAT4 | CfaAffx.9559.1.S1_at | -2,6 | 0,011 |
| serine/threonine/tyrosine interacting-like 1 | STYXL1 | CfaAffx.20862.1.S1_s_at | -2,6 | 0,042 |
| leucine rich repeat containing 48 | LRRC48 | Cfa.11662.1.A1_s_at | -2,6 | 0,005 |
| TSPY-like 2 | TSPYL2 | CfaAffx.24672.1.S1_at | -2,6 | 0,027 |
| chromodomain helicase DNA binding protein 9 | CHD9 | CfaAffx.15230.1.S1_at | -2,6 | 0,010 |
| heterogeneous nuclear ribonucleoprotein H3 (2H9) | HNRNPH3 | CfaAffx.21010.1.S1_s_at | -2,6 | 0,009 |
| similar to tudor domain containing 3 | LOC491346 | Cfa.10519.3.A1_a_at | -2,6 | 0,002 |
| bromodomain containing 3 | BRD3 | Cfa.11698.1.A1_at | -2,6 | 0,003 |
| high mobility group nucleosomal binding domain 3 | HMGN3 | Cfa.18489.1.S1_at | -2,6 | 0,011 |
| zinc finger and BTB domain containing 12 | ZBTB12 | CfaAffx.823.1.S1_at | -2,6 | 0,002 |
| SAFB-like, transcription modulator | SLTM | Cfa.2978.1.A1_at | -2,6 | 0,003 |
| similar to B0511.12 | LOC480341 | Cfa.6408.1.S1_s_at | -2,6 | 0,010 |
| coiled-coil domain containing 121 | CCDC121 | CfaAffx.8677.1.S1_at | -2,6 | 0,012 |
| axin 2 | AXIN2 | CfaAffx.17502.1.S1_at | -2,6 | 0,011 |
| keratinocyte associated protein 3 | KRTCAP3 | Cfa.6261.1.S1_at | -2,6 | 0,009 |
| MAX gene associated | MGA | CfaAffx.15182.1.S1_s_at | -2,6 | 0,032 |
| tripartite motif-containing 13 | TRIM13 | Cfa.1021.1.A1_at | -2,6 | 0,001 |
| metallophosphoesterase 1 | MPPE1 | Cfa.9403.1.A1_at | -2,6 | 0,028 |
| discs, large (Drosophila) homolog-associated protein 1 | DLGAP1 | Cfa.1358.1.A1_at | -2,6 | 0,047 |
| jumonji, AT rich interactive domain 2 | JARID2 | Cfa.15750.1.A1_at | -2,6 | 0,002 |
| suppressor of variegation 4-20 homolog 1 (Drosophila) | SUV420H1 | CfaAffx.17055.1.S1_s_at | -2,6 | 0,008 |
| zinc finger protein 300 | ZNF300 | CfaAffx.27633.1.S1_x_at | -2,5 | 0,008 |
| similar to zinc finger protein 154 (pHZ-92) | LOC491462 | CfaAffx.21926.1.S1_s_at | -2,5 | 0,008 |
| general transcription factor IIH, polypeptide 2, 44kDa | GTF2H2 | Cfa.20989.1.S1_s_at | -2,5 | 0,004 |
| zinc finger, MYND-type containing 8 | ZMYND8 | CfaAffx.16809.1.S1_at | -2,5 | 0,022 |
| selenium binding protein 1 | SELENBP1 | CfaAffx.19652.1.S1_s_at | -2,5 | 0,021 |
| cardiolipin synthase 1 | CRLS1 | Cfa.2596.1.S1_at | -2,5 | 0,004 |
| zinc finger, BED-type containing 5 | ZBED5 | Cfa.1579.1.A1_at | -2,5 | 0,009 |
| similar to SLC2A4 regulator | LOC485979 | Cfa.5458.2.A1_at | -2,5 | 0,007 |
| zinc finger CCCH-type containing 13 | ZC3H13 | CfaAffx.7719.1.S1_at | -2,5 | 0,048 |
| SH3-domain GRB2-like 3 | SH3GL3 | Cfa.16912.1.S1_at | -2,5 | 0,008 |
| protein kinase C and casein kinase substrate in neurons 3 | PACSIN3 | Cfa.2125.1.S1_at | -2,5 | 0,021 |
| similar to CG11156-PA | LOC609424 | CfaAffx.12837.1.S1_at | -2,5 | 0,013 |
| immunoglobulin superfamily, member 9 | IGSF9 | CfaAffx.18405.1.S1_s_at | -2,5 | 0,012 |
| melanoma antigen family F, 1 | MAGEF1 | Cfa.12274.1.A1_at | -2,5 | 0,003 |
| EF-hand calcium binding domain 7 | EFCAB7 | CfaAffx.28578.1.S1_at | -2,5 | 0,010 |
| similar to zinc finger protein 91 (HPF7, HTF10) | LOC485606 | CfaAffx.8697.1.S1_s_at | -2,5 | 0,022 |
| non-metastatic cells 5, protein expressed in (nucleoside-diphosphate kinase) | NME5 | CfaAffx.2635.1.S1_at | -2,5 | 0,030 |
| TIMP metallopeptidase inhibitor 3 | TIMP3 | Cfa.2272.1.A1_at | -2,5 | 0,005 |
| Alstrom syndrome 1 | ALMS1 | Cfa.11533.1.A1_at | -2,5 | 0,003 |
| Rho GTPase activating protein 8 | ARHGAP8 | CfaAffx.2220.1.S1_s_at | -2,5 | 0,023 |
| similar to Transmembrane protein 8 precursor (M83 protein) | LOC611963 | CfaAffx.4253.1.S1_at | -2,5 | 0,002 |
| pygopus homolog 1 (Drosophila) | PYGO1 | CfaAffx.24415.1.S1_at | -2,5 | 0,009 |
| EPH receptor B1 | EPHB1 | CfaAffx.11392.1.S1_s_at | -2,5 | 0,004 |
| canopy 2 homolog (zebrafish) | CNPY2 | Cfa.5346.1.A1_at | -2,5 | 0,010 |
| influenza virus NS1A binding protein | IVNS1ABP | Cfa.15025.1.S1_at | -2,5 | 0,022 |
| ATPase, Cu++ transporting, alpha polypeptide | ATP7A | Cfa.4545.1.S1_s_at | -2,5 | 0,030 |
| similar to Golgi autoantigen, golgin subfamily A member 4 | LOC477020 | CfaAffx.8158.1.S1_s_at | -2,5 | 0,024 |
| chromatin modifying protein 4C | CHMP4C | Cfa.9318.1.A1_at | -2,5 | 0,022 |
| SET domain containing 3 | SETD3 | CfaAffx.27273.1.S1_s_at | -2,5 | 0,028 |
| WW domain containing E3 ubiquitin protein ligase 2 | WWP2 | Cfa.13879.1.S1_at | -2,5 | 0,015 |
| RAD52 homolog (S. cerevisiae) | RAD52 | Cfa.17160.1.S1_at | -2,5 | 0,012 |
| SET domain containing 2 | SETD2 | Cfa.18898.1.S1_at | -2,5 | 0,021 |
| RWD domain containing 2B | RWDD2B | CfaAffx.13607.1.S1_at | -2,5 | 0,017 |
| Mdm1 nuclear protein homolog (mouse) | MDM1 | Cfa.1999.1.A1_at | -2,5 | 0,002 |
| cyclin D1 | CCND1 | Cfa.16248.1.S1_at | -2,5 | 0,018 |
| kelch domain containing 5 | KLHDC5 | Cfa.11100.1.A1_at | -2,5 | 0,003 |
| SWI/SNF related, matrix associated, actin dependent regulator of chromatin, subfamily a, member 2 | SMARCA2 | CfaAffx.3952.1.S1_s_at | -2,5 | 0,004 |
| Meis homeobox 2 | MEIS2 | CfaAffx.13535.1.S1_s_at | -2,5 | 0,041 |
| molybdenum cofactor sulfurase | MOCOS | CfaAffx.27238.1.S1_at | -2,5 | 0,015 |
| vesicle-associated membrane protein 2 (synaptobrevin 2) | VAMP2 | Cfa.20442.1.S1_at | -2,5 | 0,015 |
| nicotinamide nucleotide transhydrogenase | NNT | Cfa.15220.2.A1_at | -2,5 | 0,021 |
| aldehyde dehydrogenase 7 family, member A1 | ALDH7A1 | Cfa.15706.1.A1_at | -2,5 | 0,007 |
| carbonic anhydrase XI | CA11 | CfaAffx.6852.1.S1_s_at | -2,5 | 0,021 |
| enolase 3 (beta, muscle) | ENO3 | CfaAffx.24225.1.S1_s_at | -2,5 | 0,007 |
| armadillo repeat containing 8 | ARMC8 | Cfa.13309.1.A1_at | -2,5 | 0,001 |
| tetratricopeptide repeat domain 3 | TTC3 | Cfa.251.1.S1_at | -2,4 | 0,003 |
| ankyrin repeat and SOCS box-containing 1 | ASB1 | CfaAffx.19331.1.S1_at | -2,4 | 0,006 |
| LPS-responsive vesicle trafficking, beach and anchor containing | LRBA | Cfa.1974.1.A1_at | -2,4 | 0,019 |
| taspase, threonine aspartase, 1 | TASP1 | CfaAffx.9349.1.S1_at | -2,4 | 0,012 |
| similar to ribosomal protein L30 /// ribosomal protein L30 | RPL30 | Cfa.5994.3.S1_at | -2,4 | 0,001 |
| proteasome (prosome, macropain) 26S subunit, ATPase, 6 | PSMC6 | Cfa.638.1.S1_at | -2,4 | 0,013 |
| similar to dynein, cytoplasmic, heavy polypeptide 2 | LOC479461 | CfaAffx.23098.1.S1_s_at | -2,4 | 0,009 |
| SH3-domain binding protein 4 | SH3BP4 | CfaAffx.18664.1.S1_at | -2,4 | 0,046 |
| nischarin | NISCH | Cfa.4335.1.S1_at | -2,4 | 0,010 |
| PRP40 pre-mRNA processing factor 40 homolog B (S. cerevisiae) | PRPF40B | CfaAffx.13485.1.S1_s_at | -2,4 | 0,008 |
| SWI/SNF related, matrix associated, actin dependent regulator of chromatin, subfamily e, member 1 | SMARCE1 | Cfa.8883.1.A1_at | -2,4 | 0,019 |
| sirtuin (silent mating type information regulation 2 homolog) 3 (S. cerevisiae) | SIRT3 | Cfa.11529.1.A1_at | -2,4 | 0,003 |
| endosulfine alpha | ENSA | Cfa.15126.1.S1_at | -2,4 | 0,046 |
| adaptor protein, phosphotyrosine interaction, PH domain and leucine zipper containing 2 | APPL2 | CfaAffx.3751.1.S1_at | -2,4 | 0,015 |
| casein kinase 2, alpha 1 polypeptide | CSNK2A1 | Cfa.10060.1.A1_at | -2,4 | 0,005 |
| reticulocalbin 2, EF-hand calcium binding domain | RCN2 | Cfa.21641.1.S1_at | -2,4 | 0,037 |
| SFT2 domain containing 1 | SFT2D1 | Cfa.9210.2.A1_at | -2,4 | 0,021 |
| centrosomal protein 110kDa | CEP110 | CfaAffx.6370.1.S1_s_at | -2,4 | 0,020 |
| similar to heat shock protein 8 | LOC609874 | Cfa.18310.1.S1_s_at | -2,4 | 0,041 |
| CTP synthase II | CTPS2 | CfaAffx.19379.1.S1_s_at | -2,4 | 0,010 |
| ADP-ribosylhydrolase like 2 | ADPRHL2 | CfaAffx.6222.1.S1_at | -2,4 | 0,001 |
| tau tubulin kinase 2 | TTBK2 | Cfa.10083.1.S1_at | -2,4 | 0,016 |
| enhancer of polycomb homolog 1 (Drosophila) | EPC1 | CfaAffx.7061.1.S1_at | -2,4 | 0,009 |
| frizzled homolog 6 (Drosophila) | FZD6 | Cfa.3472.1.S1_s_at | -2,4 | 0,017 |
| membrane-associated ring finger (C3HC4) 7 | MARCH7 | Cfa.18596.2.S1_s_at | -2,4 | 0,033 |
| replication factor C (activator 1) 1, 145kDa | RFC1 | Cfa.2351.1.S1_at | -2,4 | 0,014 |
| similar to myeloid/lymphoid or mixed-lineage leukemia 3 isoform 1 | LOC611073 | CfaAffx.8349.1.S1_at | -2,4 | 0,033 |
| similar to Netrin G1 precursor (Laminet-1) | LOC490132 | Cfa.9115.1.A1_at | -2,4 | 0,016 |
| poly(A) binding protein interacting protein 2B | PAIP2B | Cfa.18113.1.S1_at | -2,4 | 0,028 |
| tetratricopeptide repeat domain 21A | TTC21A | Cfa.7003.1.A1_at | -2,4 | 0,004 |
| threonine synthase-like 1 (S. cerevisiae) | THNSL1 | Cfa.11067.1.A1_at | -2,4 | 0,011 |
| solute carrier family 2 (facilitated glucose transporter), member 12 | SLC2A12 | CfaAffx.1284.1.S1_at | -2,4 | 0,033 |
| transcriptional adaptor 1 (HFI1 homolog, yeast)-like | TADA1L | CfaAffx.23924.1.S1_at | -2,4 | 0,017 |
| similar to Temporarily Assigned Gene name family member (tag-278) | LOC476274 | Cfa.20379.1.S1_s_at | -2,4 | 0,013 |
| REV3-like, catalytic subunit of DNA polymerase zeta (yeast) | REV3L | CfaAffx.6861.1.S1_s_at | -2,4 | 0,014 |
| A kinase (PRKA) anchor protein (yotiao) 9 | AKAP9 | Cfa.3334.1.A1_s_at | -2,4 | 0,011 |
| betaine-homocysteine methyltransferase 2 | BHMT2 | CfaAffx.14367.1.S1_at | -2,4 | 0,040 |
| HLA-B associated transcript 1 | BAT1 | Cfa.10131.1.S1_at | -2,4 | 0,011 |
| phosphorylase, glycogen, liver | PYGL | Cfa.10412.1.A1_s_at | -2,4 | 0,014 |
| S phase cyclin A-associated protein in the ER | SCAPER | CfaAffx.27748.1.S1_s_at | -2,4 | 0,007 |
| fibroblast growth factor 12 | FGF12 | Cfa.19396.1.S1_s_at | -2,4 | 0,013 |
| phosphatidylinositol glycan anchor biosynthesis, class G | PIGG | Cfa.13664.1.A1_at | -2,4 | 0,008 |
| SMEK homolog 1, suppressor of mek1 (Dictyostelium) | SMEK1 | CfaAffx.26898.1.S1_at | -2,4 | 0,002 |
| prostaglandin D2 synthase 21kDa (brain) | PTGDS | Cfa.1282.1.A1_s_at | -2,4 | 0,035 |
| ubiquitin family domain containing 1 | UBFD1 | Cfa.2057.1.A1_at | -2,4 | 0,015 |
| solute carrier family 25, member 13 (citrin) | SLC25A13 | CfaAffx.4146.1.S1_s_at | -2,3 | 0,047 |
| RWD domain containing 3 | RWDD3 | CfaAffx.30765.1.S1_at | -2,3 | 0,015 |
| zinc finger and BTB domain containing 16 | ZBTB16 | CfaAffx.20922.1.S1_at | -2,3 | 0,010 |
| similar to TG-interacting factor isoform c | LOC490537 | CfaAffx.18412.1.S1_s_at | -2,3 | 0,005 |
| zinc finger family member 674 | ZNF674 | CfaAffx.22707.1.S1_s_at | -2,3 | 0,019 |
| PARK2 co-regulated | PACRG | Cfa.12073.1.A1_at | -2,3 | 0,031 |
| fructosamine 3 kinase | FN3K | CfaAffx.20168.1.S1_s_at | -2,3 | 0,040 |
| myotubularin related protein 15 | MTMR15 | Cfa.10556.1.A1_at | -2,3 | 0,024 |
| O-linked N-acetylglucosamine (GlcNAc) transferase (UDP-N-acetylglucosamine:polypeptide-N-acetylglucosaminyl transferase) | OGT | Cfa.15244.1.S1_at | -2,3 | 0,007 |
| SEC14 and spectrin domains 1 | SESTD1 | CfaAffx.21778.1.S1_at | -2,3 | 0,007 |
| similar to CG6454-PB, isoform B | LOC477671 | Cfa.504.1.S1_at | -2,3 | 0,001 |
| WD repeat domain 35 | WDR35 | CfaAffx.6650.1.S1_at | -2,3 | 0,031 |
| similar to proline-rich cyclin A1-interacting protein | LOC610597 | Cfa.7025.1.A1_at | -2,3 | 0,026 |
| methyltransferase like 5 | METTL5 | Cfa.3271.1.S1_at | -2,3 | 0,007 |
| LATS, large tumor suppressor, homolog 1 (Drosophila) | LATS1 | Cfa.2347.1.S1_at | -2,3 | 0,005 |
| similar to KIAA1875 protein | LOC482088 | CfaAffx.3203.1.S1_at | -2,3 | 0,016 |
| similar to EMSY protein | LOC476800 | Cfa.2221.1.A1_s_at | -2,3 | 0,009 |
| armadillo repeat containing 4 | ARMC4 | CfaAffx.22402.1.S1_s_at | -2,3 | 0,020 |
| BMI1 polycomb ring finger oncogene | BMI1 | CfaAffx.7166.1.S1_s_at | -2,3 | 0,005 |
| xeroderma pigmentosum, complementation group C | XPC | Cfa.9082.1.A1_at | -2,3 | 0,005 |
| similar to CG13018-PA | LOC611656 | Cfa.9381.1.A1_at | -2,3 | 0,012 |
| similar to CG15120-PA | LOC479130 | Cfa.1857.1.S1_at | -2,3 | 0,007 |
| spermatogenesis associated 7 | SPATA7 | CfaAffx.26551.1.S1_s_at | -2,3 | 0,008 |
| pericentriolar material 1 | PCM1 | Cfa.20183.1.S1_at | -2,3 | 0,007 |
| hect domain and RLD 2 | HERC2 | Cfa.5938.1.A1_at | -2,3 | 0,027 |
| dimethylarginine dimethylaminohydrolase 2 | DDAH2 | Cfa.19622.1.S1_at | -2,3 | 0,004 |
| intraflagellar transport 172 homolog (Chlamydomonas) | IFT172 | Cfa.2982.1.S1_s_at | -2,3 | 0,012 |
| zinc finger, RAN-binding domain containing 1 | ZRANB1 | CfaAffx.19764.1.S1_s_at | -2,3 | 0,008 |
| mitogen-activated protein kinase kinase kinase 4 | MAP3K4 | Cfa.12563.1.A1_at | -2,3 | 0,022 |
| chondroitin sulfate N-acetylgalactosaminyltransferase 1 | CSGALNACT1 | CfaAffx.8950.1.S1_at | -2,3 | 0,021 |
| spastic paraplegia 20 (Troyer syndrome) | SPG20 | Cfa.339.1.S1_at | -2,3 | 0,005 |
| hepatocyte nuclear factor 4, gamma | HNF4G | CfaAffx.13163.1.S1_at | -2,3 | 0,031 |
| tectonic family member 2 | TCTN2 | Cfa.5605.1.A1_at | -2,3 | 0,034 |
| alpha thalassemia/mental retardation syndrome X-linked (RAD54 homolog, S. cerevisiae) | ATRX | CfaAffx.26393.1.S1_s_at | -2,3 | 0,009 |
| F-box and leucine-rich repeat protein 10 | FBXL10 | Cfa.6161.1.S1_s_at | -2,3 | 0,001 |
| similar to fusion (involved in t(12;16) in malignant liposarcoma) (predicted) | LOC479778 | Cfa.245.1.S1_at | -2,3 | 0,002 |
| MYCBP associated protein | MYCBPAP | Cfa.6719.1.A1_a_at | -2,3 | 0,048 |
| discoidin domain receptor tyrosine kinase 1 | DDR1 | Cfa.7473.1.A1_at | -2,3 | 0,019 |
| mediator complex subunit 25 | MED25 | CfaAffx.6062.1.S1_s_at | -2,3 | 0,007 |
| TSC22 domain family, member 3 | TSC22D3 | Cfa.10310.1.S1_at | -2,3 | 0,020 |
| LYR motif containing 2 | LYRM2 | CfaAffx.5601.1.S1_at | -2,3 | 0,003 |
| solute carrier family 22, member 17 | SLC22A17 | Cfa.3972.1.A1_at | -2,3 | 0,027 |
| glioma tumor suppressor candidate region gene 2 | GLTSCR2 | CfaAffx.7037.1.S1_s_at | -2,3 | 0,010 |
| aftiphilin | AFTPH | Cfa.4112.1.S1_at | -2,3 | 0,001 |
| WD repeat domain 90 | WDR90 | Cfa.12102.1.A1_at | -2,3 | 0,010 |
| E2F transcription factor 5, p130-binding | E2F5 | CfaAffx.13633.1.S1_at | -2,3 | 0,034 |
| SET domain containing 6 | SETD6 | Cfa.10445.1.A1_at | -2,3 | 0,013 |
| IQ motif containing B1 | IQCB1 | CfaAffx.18102.1.S1_at | -2,3 | 0,029 |
| plakophilin 4 | PKP4 | CfaAffx.14770.1.S1_at | -2,3 | 0,026 |
| peroxisomal biogenesis factor 1 | PEX1 | Cfa.1438.1.S1_at | -2,3 | 0,004 |
| similar to Kinesin-Like Protein family member (klp-6) | LOC490381 | Cfa.12415.1.A1_at | -2,3 | 0,013 |
| oligonucleotide/oligosaccharide-binding fold containing 1 | OBFC1 | Cfa.2571.1.A1_at | -2,3 | 0,009 |
| A kinase (PRKA) anchor protein 7 | AKAP7 | CfaAffx.2601.1.S1_at | -2,3 | 0,004 |
| bromodomain PHD finger transcription factor | BPTF | CfaAffx.17996.1.S1_s_at | -2,3 | 0,022 |
| splicing factor, arginine/serine-rich 8 (suppressor-of-white-apricot homolog, Drosophila) | SFRS8 | CfaAffx.10892.1.S1_s_at | -2,3 | 0,009 |
| ATP-binding cassette, sub-family C (CFTR/MRP), member 5 | ABCC5 | Cfa.19115.1.S1_at | -2,3 | 0,033 |
| G patch domain containing 1 | GPATCH1 | Cfa.10315.1.A1_at | -2,3 | 0,002 |
| calcium/calmodulin-dependent serine protein kinase (MAGUK family) | CASK | CfaAffx.22202.1.S1_at | -2,3 | 0,001 |
| ankyrin repeat domain 10 | ANKRD10 | CfaAffx.10145.1.S1_at | -2,3 | 0,001 |
| laminin, beta 1 | LAMB1 | Cfa.18707.1.S1_s_at | -2,3 | 0,041 |
| Dmx-like 1 | DMXL1 | Cfa.19662.1.S1_at | -2,3 | 0,010 |
| similar to amyotrophic lateral sclerosis 2 (juvenile) chromosome region, candidate 8 | LOC478877 | CfaAffx.19456.1.S1_at | -2,3 | 0,010 |
| antizyme inhibitor 1 | AZIN1 | Cfa.4399.1.S1_at | -2,3 | 0,026 |
| small nuclear ribonucleoprotein 70kDa polypeptide (RNP antigen) | SNRP70 | Cfa.17797.1.S1_at | -2,2 | 0,030 |
| Sin3A-associated protein, 130kDa | SAP130 | CfaAffx.7367.1.S1_s_at | -2,2 | 0,014 |
| similar to mixed lineage kinase-related kinase MRK-beta isoform 2 | LOC478802 | Cfa.21372.1.S1_s_at | -2,2 | 0,026 |
| meningioma expressed antigen 5 (hyaluronidase) | MGEA5 | Cfa.12884.1.S1_at | -2,2 | 0,019 |
| zinc finger protein 638 | ZNF638 | CfaAffx.14326.1.S1_s_at | -2,2 | 0,010 |
| zinc and ring finger 3 | ZNRF3 | CfaAffx.18602.1.S1_s_at | -2,2 | 0,002 |
| trinucleotide repeat containing 6B | TNRC6B | CfaAffx.2813.1.S1_s_at | -2,2 | 0,027 |
| MAP3K12 binding inhibitory protein 1 | MBIP | Cfa.20159.1.S1_s_at | -2,2 | 0,010 |
| tumor protein p53 binding protein, 2 | TP53BP2 | CfaAffx.25128.1.S1_s_at | -2,2 | 0,012 |
| t-complex-associated-testis-expressed 1-like | TCTE1L | Cfa.217.1.S1_s_at | -2,2 | 0,005 |
| spectrin repeat containing, nuclear envelope 2 | SYNE2 | Cfa.9691.1.A1_at | -2,2 | 0,009 |
| peroxisomal biogenesis factor 6 | PEX6 | CfaAffx.3520.1.S1_at | -2,2 | 0,004 |
| similar to CG14182-PA | LOC608733 | CfaAffx.27161.1.S1_at | -2,2 | 0,008 |
| reticulon 4 interacting protein 1 | RTN4IP1 | Cfa.13506.1.A1_s_at | -2,2 | 0,012 |
| proliferation-associated 2G4, 38kDa | PA2G4 | CfaAffx.1122.1.S1_at | -2,2 | 0,048 |
| AT rich interactive domain 1A (SWI-like) | ARID1A | Cfa.6100.1.A1_at | -2,2 | 0,014 |
| cholinergic receptor, nicotinic, epsilon | CHRNE | CfaAffx.24261.1.S1_at | -2,2 | 0,005 |
| nicotinate phosphoribosyltransferase domain containing 1 | NAPRT1 | Cfa.17805.1.S1_at | -2,2 | 0,005 |
| polyhomeotic homolog 1 (Drosophila) | PHC1 | Cfa.1968.1.S1_at | -2,2 | 0,004 |
| centrosome and spindle pole associated protein 1 | CSPP1 | CfaAffx.11995.1.S1_s_at | -2,2 | 0,002 |
| glucosidase, beta (bile acid) 2 | GBA2 | Cfa.7734.1.A1_at | -2,2 | 0,024 |
| Wiskott-Aldrich syndrome-like | WASL | Cfa.20419.1.A1_s_at | -2,2 | 0,008 |
| zinc finger with KRAB and SCAN domains 5 | ZKSCAN5 | CfaAffx.23053.1.S1_s_at | -2,2 | 0,014 |
| telomerase-associated protein 1 | TEP1 | CfaAffx.9036.1.S1_s_at | -2,2 | 0,033 |
| zinc finger protein 283 | ZNF283 | CfaAffx.8009.1.S1_s_at | -2,2 | 0,041 |
| coiled-coil domain containing 89 | CCDC89 | Cfa.2611.1.A1_at | -2,2 | 0,012 |
| similar to Nck-associated protein 5 | LOC483893 | CfaAffx.8422.1.S1_at | -2,2 | 0,038 |
| G protein-coupled receptor kinase 4 | GRK4 | CfaAffx.22661.1.S1_s_at | -2,2 | 0,019 |
| kelch-like 11 (Drosophila) | KLHL11 | CfaAffx.24279.1.S1_s_at | -2,2 | 0,042 |
| similar to signal peptide peptidase 3 | LOC486301 | Cfa.7149.1.A1_at | -2,2 | 0,011 |
| zinc finger protein 33A | ZNF33A | CfaAffx.15585.1.S1_s_at | -2,2 | 0,023 |
| deoxyguanosine kinase | DGUOK | Cfa.14435.1.A1_at | -2,2 | 0,001 |
| coiled-coil domain containing 55 | CCDC55 | CfaAffx.28983.1.S1_at | -2,2 | 0,004 |
| similar to CDC-like kinase 1 isoform 1 | LOC478866 | Cfa.17292.2.S1_s_at | -2,2 | 0,035 |
| nuclear receptor coactivator 1 | NCOA1 | Cfa.10398.1.A1_at | -2,2 | 0,006 |
| coiled-coil domain containing 34 | CCDC34 | CfaAffx.15928.1.S1_s_at | -2,2 | 0,013 |
| zinc finger, AN1-type domain 5 | ZFAND5 | Cfa.10843.1.A1_at | -2,2 | 0,016 |
| IQ motif containing K | IQCK | CfaAffx.27617.1.S1_at | -2,2 | 0,042 |
| zinc finger protein 227 | ZNF227 | CfaAffx.7983.1.S1_s_at | -2,2 | 0,014 |
| EPH receptor B3 | EPHB3 | CfaAffx.20214.1.S1_s_at | -2,2 | 0,019 |
| similar to oxidored-nitro domain-containing protein isoform 1 | LOC475334 | CfaAffx.6001.1.S1_at | -2,2 | 0,037 |
| DEP domain containing 5 | DEPDC5 | Cfa.11556.1.A1_at | -2,2 | 0,014 |
| lon peptidase 2, peroxisomal | LONP2 | CfaAffx.15744.1.S1_s_at | -2,2 | 0,020 |
| RING1 and YY1 binding protein | RYBP | Cfa.2540.1.S1_at | -2,2 | 0,012 |
| RNA binding motif protein 4B | RBM4B | CfaAffx.19209.1.S1_s_at | -2,2 | 0,010 |
| matrilin 2 | MATN2 | Cfa.9487.1.A1_at | -2,2 | 0,002 |
| amyloid beta (A4) precursor protein-binding, family B, member 3 | APBB3 | Cfa.8054.1.S1_s_at | -2,2 | 0,005 |
| ES cell expressed Ras | ERAS | Cfa.7205.1.A1_at | -2,2 | 0,020 |
| Sp1 transcription factor | SP1 | Cfa.7309.1.A1_at | -2,2 | 0,049 |
| serine/arginine repetitive matrix 2 | SRRM2 | Cfa.21637.1.S1_s_at | -2,2 | 0,031 |
| rabphilin 3A-like (without C2 domains) | RPH3AL | Cfa.11038.1.A1_at | -2,2 | 0,013 |
| glutaredoxin 5 | GLRX5 | CfaAffx.306.1.S1_s_at | -2,2 | 0,044 |
| Ewing sarcoma breakpoint region 1 | EWSR1 | Cfa.9398.1.A1_at | -2,2 | 0,007 |
| leucine zipper, down-regulated in cancer 1 | LDOC1 | Cfa.4103.1.A1_at | -2,2 | 0,001 |
| dishevelled associated activator of morphogenesis 1 | DAAM1 | CfaAffx.23818.1.S1_s_at | -2,2 | 0,000 |
| protein phosphatase 1, regulatory (inhibitor) subunit 9A | PPP1R9A | CfaAffx.4074.1.S1_at | -2,2 | 0,013 |
| similar to Protein C14orf93 precursor | LOC480245 | Cfa.4800.1.A1_at | -2,2 | 0,014 |
| GC-rich promoter binding protein 1 | GPBP1 | Cfa.1155.1.S1_at | -2,2 | 0,002 |
| phosphatidylinositol glycan anchor biosynthesis, class N | PIGN | Cfa.3287.1.A1_at | -2,2 | 0,007 |
| endomucin | EMCN | CfaAffx.16621.1.S1_s_at | -2,2 | 0,037 |
| POZ (BTB) and AT hook containing zinc finger 1 | PATZ1 | CfaAffx.20256.1.S1_s_at | -2,2 | 0,020 |
| proline rich 6 | PRR6 | CfaAffx.27600.1.S1_at | -2,2 | 0,030 |
| DCP1 decapping enzyme homolog B (S. cerevisiae) | DCP1B | Cfa.965.1.S1_at | -2,2 | 0,004 |
| similar to zinc finger, DHHC domain containing 1 | LOC479686 | CfaAffx.31178.1.S1_at | -2,2 | 0,008 |
| similar to Zinc finger protein 38 (Zinc finger protein KOX25) (Zinc finger protein HF.12) (Zinc finger protein 3) (HZF3.1 protein) | LOC489879 | Cfa.15728.1.A1_at | -2,2 | 0,027 |
| Wolf-Hirschhorn syndrome candidate 1-like 1 | WHSC1L1 | Cfa.1712.1.A1_at | -2,2 | 0,018 |
| similar to limkain b1 isoform 1 | LOC479837 | Cfa.19892.1.S1_at | -2,2 | 0,040 |
| zinc finger, CCHC domain containing 11 | ZCCHC11 | Cfa.2227.1.A1_s_at | -2,2 | 0,008 |
| NCK-associated protein 1 | NCKAP1 | Cfa.19712.1.S1_s_at | -2,2 | 0,009 |
| tRNA-yW synthesizing protein 3 homolog (S. cerevisiae) | TYW3 | Cfa.12354.1.A1_a_at | -2,2 | 0,018 |
| coiled-coil domain containing 77 | CCDC77 | CfaAffx.24191.1.S1_s_at | -2,1 | 0,017 |
| mitofusin 1 | MFN1 | Cfa.19756.1.S1_at | -2,1 | 0,018 |
| WW domain containing E3 ubiquitin protein ligase 1 | WWP1 | CfaAffx.13908.1.S1_at | -2,1 | 0,046 |
| cardiomyopathy associated 5 | CMYA5 | Cfa.2645.1.S1_at | -2,1 | 0,006 |
| nucleolar protein 12 | NOL12 | CfaAffx.3108.1.S1_s_at | -2,1 | 0,002 |
| SHC (Src homology 2 domain containing) transforming protein 2 | SHC2 | Cfa.17635.1.S1_at | -2,1 | 0,041 |
| Tctex1 domain containing 2 | TCTEX1D2 | Cfa.19204.1.S1_s_at | -2,1 | 0,007 |
| CWC15 homolog (S. cerevisiae) | CWC15 | Cfa.12763.1.A1_s_at | -2,1 | 0,009 |
| TRAF2 and NCK interacting kinase | TNIK | CfaAffx.23355.1.S1_s_at | -2,1 | 0,023 |
| heterogeneous nuclear ribonucleoprotein K | HNRNPK | Cfa.221.1.A1_at | -2,1 | 0,012 |
| gephyrin | GPHN | Cfa.10313.1.A1_s_at | -2,1 | 0,003 |
| zinc finger, MYM-type 4 | ZMYM4 | CfaAffx.6330.1.S1_s_at | -2,1 | 0,003 |
| microtubule-actin crosslinking factor 1 | MACF1 | CfaAffx.5703.1.S1_at | -2,1 | 0,029 |
| heat shock 70kDa protein 4-like | HSPA4L | Cfa.19944.1.S1_s_at | -2,1 | 0,012 |
| keratin 24 | KRT24 | CfaAffx.24570.1.S1_at | -2,1 | 0,020 |
| integrator complex subunit 8 | INTS8 | CfaAffx.14816.1.S1_s_at | -2,1 | 0,007 |
| TM2 domain containing 2 | TM2D2 | Cfa.2002.1.S1_at | -2,1 | 0,013 |
| ubiquitously transcribed tetratricopeptide repeat, X chromosome | UTX | CfaAffx.22557.1.S1_s_at | -2,1 | 0,014 |
| protein (peptidylprolyl cis/trans isomerase) NIMA-interacting, 4 (parvulin) | PIN4 | Cfa.12056.1.A1_s_at | -2,1 | 0,010 |
| DAZ interacting protein 3, zinc finger | DZIP3 | CfaAffx.16078.1.S1_s_at | -2,1 | 0,035 |
| PDZ and LIM domain 4 | PDLIM4 | CfaAffx.2165.1.S1_s_at | -2,1 | 0,041 |
| monoglyceride lipase | MGLL | Cfa.4090.1.A1_s_at | -2,1 | 0,035 |
| asparaginyl-tRNA synthetase 2, mitochondrial (putative) | NARS2 | CfaAffx.8096.1.S1_s_at | -2,1 | 0,014 |
| polycomb group ring finger 1 | PCGF1 | Cfa.14740.1.A1_s_at | -2,1 | 0,033 |
| asparagine-linked glycosylation 13 homolog (S. cerevisiae) | ALG13 | CfaAffx.27861.1.S1_at | -2,1 | 0,017 |
| similar to Zinc finger protein 208 | LOC484461 | CfaAffx.7990.1.S1_x_at | -2,1 | 0,016 |
| centromere protein C 1 | CENPC1 | Cfa.1767.2.A1_at | -2,1 | 0,011 |
| similar to YLP motif containing protein 1 (Nuclear protein ZAP3) (ZAP113) | LOC480387 | CfaAffx.25967.1.S1_at | -2,1 | 0,012 |
| Rho GTPase activating protein 5 | ARHGAP5 | CfaAffx.20369.1.S1_s_at | -2,1 | 0,013 |
| similar to zinc finger and BTB domain containing 8 /// zinc finger and BTB domain containing 8 | ZBTB8 | CfaAffx.16345.1.S1_s_at | -2,1 | 0,009 |
| poly (ADP-ribose) polymerase 2 | PARP2 | Cfa.11307.1.A1_at | -2,1 | 0,006 |
| choline kinase alpha | CHKA | Cfa.8353.1.A1_at | -2,1 | 0,003 |
| similar to SH3 domain-containing adapter protein | LOC611699 | CfaAffx.20262.1.S1_at | -2,1 | 0,005 |
| polybromo 1 | PBRM1 | Cfa.8067.1.S1_at | -2,1 | 0,032 |
| methylphosphate capping enzyme | MEPCE | Cfa.16135.1.S1_at | -2,1 | 0,008 |
| interleukin-1 receptor-associated kinase 1 binding protein 1 | IRAK1BP1 | CfaAffx.5105.1.S1_at | -2,1 | 0,008 |
| general transcription factor II, i | GTF2I | Cfa.4193.1.S1_at | -2,1 | 0,002 |
| MIT, microtubule interacting and transport, domain containing 1 | MITD1 | Cfa.13593.1.S1_s_at | -2,1 | 0,010 |
| stromal antigen 1 | STAG1 | CfaAffx.11998.1.S1_at | -2,1 | 0,030 |
| similar to CG11638-PA | LOC480414 | Cfa.18211.1.A1_s_at | -2,1 | 0,041 |
| homeobox B4 | HOXB4 | CfaAffx.25756.1.S1_at | -2,1 | 0,002 |
| bromodomain containing 1 | BRD1 | Cfa.2453.1.A1_at | -2,1 | 0,019 |
| similar to eukaryotic translation initiation factor 3, subunit 5 epsilon, 47kDa | LOC476840 | CfaAffx.11227.1.S1_at | -2,1 | 0,004 |
| RNA binding motif protein 17 | RBM17 | Cfa.3967.1.A1_at | -2,1 | 0,010 |
| CREB binding protein | CREBBP | Cfa.8999.1.A1_at | -2,1 | 0,037 |
| CDK5 regulatory subunit associated protein 1-like 1 | CDKAL1 | CfaAffx.16006.1.S1_s_at | -2,1 | 0,002 |
| arylsulfatase family, member K | ARSK | CfaAffx.12692.1.S1_at | -2,1 | 0,001 |
| FERM and PDZ domain containing 4 | FRMPD4 | CfaAffx.18076.1.S1_at | -2,1 | 0,021 |
| growth factor receptor-bound protein 7 | GRB7 | CfaAffx.25031.1.S1_at | -2,1 | 0,028 |
| SWI/SNF related, matrix associated, actin dependent regulator of chromatin, subfamily c, member 2 | SMARCC2 | Cfa.16860.1.S1_s_at | -2,1 | 0,046 |
| GTF2I repeat domain containing 2 | GTF2IRD2 | CfaAffx.694.1.S1_at | -2,1 | 0,036 |
| Nipped-B homolog (Drosophila) | NIPBL | CfaAffx.28575.1.S1_at | -2,1 | 0,019 |
| ATPase, Ca++ transporting, cardiac muscle, slow twitch 2 | ATP2A2 | Cfa.97.1.S1_s_at | -2,1 | 0,011 |
| phosphatidic acid phosphatase type 2B | PPAP2B | Cfa.10489.1.A1_s_at | -2,1 | 0,021 |
| similar to CG6931-PA | LOC480156 | CfaAffx.27185.1.S1_s_at | -2,1 | 0,022 |
| ADP-ribosylation factor-like 6 interacting protein 2 | ARL6IP2 | Cfa.603.1.S1_at | -2,1 | 0,004 |
| zinc finger, FYVE domain containing 20 | ZFYVE20 | Cfa.9689.1.S1_at | -2,1 | 0,003 |
| similar to Core histone macro-H2A.2 (Histone macroH2A2) (mH2A2) | LOC489024 | CfaAffx.21622.1.S1_at | -2,1 | 0,009 |
| TAR (HIV-1) RNA binding protein 1 | TARBP1 | CfaAffx.17925.1.S1_at | -2,1 | 0,010 |
| apoptotic chromatin condensation inducer 1 | ACIN1 | Cfa.20285.1.S1_s_at | -2,1 | 0,046 |
| PHD finger protein 17 | PHF17 | Cfa.1602.1.A1_at | -2,1 | 0,006 |
| eukaryotic translation initiation factor 4E nuclear import factor 1 | EIF4ENIF1 | Cfa.4972.1.A1_at | -2,1 | 0,004 |
| sorting nexin family member 21 | SNX21 | Cfa.14233.1.A1_at | -2,1 | 0,027 |
| similar to CG15133-PA | LOC483843 | CfaAffx.6962.1.S1_at | -2,1 | 0,039 |
| zinc finger, SWIM-type containing 7 | ZSWIM7 | Cfa.11779.1.A1_at | -2,1 | 0,044 |
| CWF19-like 2, cell cycle control (S. pombe) | CWF19L2 | Cfa.9648.1.A1_at | -2,1 | 0,028 |
| component of oligomeric golgi complex 1 | COG1 | Cfa.10537.1.A1_at | -2,1 | 0,022 |
| ubiquitin specific peptidase 19 | USP19 | CfaAffx.18105.1.S1_s_at | -2,1 | 0,002 |
| ubiquitin specific peptidase 42 | USP42 | CfaAffx.24026.1.S1_at | -2,1 | 0,005 |
| RPGRIP1-like | RPGRIP1L | CfaAffx.14928.1.S1_s_at | -2,1 | 0,008 |
| metal response element binding transcription factor 2 | MTF2 | Cfa.1889.2.S1_at | -2,1 | 0,026 |
| myosin IXA | MYO9A | CfaAffx.27006.1.S1_at | -2,1 | 0,049 |
| chromodomain helicase DNA binding protein 1 | CHD1 | Cfa.2790.1.A1_s_at | -2,0 | 0,031 |
| similar to mitochondrial ferritin | LOC608550 | Cfa.10904.1.S1_s_at | -2,0 | 0,045 |
| BEX family member 4 | BEX4 | CfaAffx.7554.1.S1_s_at | -2,0 | 0,003 |
| gem (nuclear organelle) associated protein 6 | GEMIN6 | Cfa.10443.1.A1_s_at | -2,0 | 0,009 |
| zinc finger protein 333 | ZNF333 | CfaAffx.24917.1.S1_at | -2,0 | 0,021 |
| RAD54-like 2 (S. cerevisiae) | RAD54L2 | Cfa.11800.1.A1_s_at | -2,0 | 0,019 |
| vacuolar protein sorting 13 homolog A (S. cerevisiae) | VPS13A | CfaAffx.3360.1.S1_s_at | -2,0 | 0,034 |
| zinc finger, MYND domain containing 11 | ZMYND11 | Cfa.4247.1.A1_at | -2,0 | 0,001 |
| eukaryotic translation initiation factor 2 alpha kinase 4 | EIF2AK4 | Cfa.19193.1.S1_s_at | -2,0 | 0,002 |
| poly (ADP-ribose) polymerase family, member 6 | PARP6 | CfaAffx.27066.1.S1_s_at | -2,0 | 0,006 |
| heat shock 70kDa protein 1-like | HSPA1L | Cfa.8759.1.A1_at | -2,0 | 0,016 |
| leucine rich repeat containing 42 | LRRC42 | CfaAffx.29114.1.S1_at | -2,0 | 0,046 |
| intraflagellar transport 81 homolog (Chlamydomonas) | IFT81 | CfaAffx.13362.1.S1_at | -2,0 | 0,034 |
| CSRP2 binding protein | CSRP2BP | Cfa.10378.1.A1_at | -2,0 | 0,003 |
| CCR4-NOT transcription complex, subunit 4 | CNOT4 | CfaAffx.5770.1.S1_s_at | -2,0 | 0,018 |
| tripartite motif-containing 45 | TRIM45 | Cfa.7338.1.A1_at | -2,0 | 0,034 |
| asteroid homolog 1 (Drosophila) | ASTE1 | CfaAffx.10139.1.S1_at | -2,0 | 0,009 |
| pre-B-cell leukemia homeobox 3 | PBX3 | Cfa.7313.1.A1_at | -2,0 | 0,019 |
| adenomatous polyposis coli | APC | CfaAffx.11870.1.S1_s_at | -2,0 | 0,041 |
| similar to soluble adenylyl cyclase | LOC608939 | Cfa.9975.1.A1_at | -2,0 | 0,019 |
| nuclear receptor coactivator 6 | NCOA6 | Cfa.1642.2.S1_s_at | -2,0 | 0,009 |
| similar to CG6454-PB, isoform B | LOC477671 | CfaAffx.18368.1.S1_s_at | -2,0 | 0,038 |
| ubiquitin specific peptidase 34 | USP34 | CfaAffx.5486.1.S1_s_at | -2,0 | 0,010 |
| DEAH (Asp-Glu-Ala-His) box polypeptide 37 | DHX37 | CfaAffx.11316.1.S1_at | -2,0 | 0,007 |
| PHD finger protein 10 | PHF10 | Cfa.10525.1.A1_s_at | -2,0 | 0,023 |
| coiled-coil domain containing 14 | CCDC14 | CfaAffx.18947.1.S1_at | -2,0 | 0,013 |
| SFRS protein kinase 2 | SRPK2 | Cfa.10743.1.S1_s_at | -2,0 | 0,003 |
| DNA (cytosine-5-)-methyltransferase 3 alpha | DNMT3A | CfaAffx.7164.1.S1_s_at | -2,0 | 0,031 |
| adiponectin receptor 2 | ADIPOR2 | Cfa.10882.1.A1_at | -2,0 | 0,018 |
| COBW domain containing 2 | CBWD2 | Cfa.1676.1.A1_at | -2,0 | 0,046 |
| WD repeat domain 48 | WDR48 | Cfa.2333.1.S1_a_at | -2,0 | 0,013 |
| similar to H23L24.3a | LOC491355 | CfaAffx.31060.1.S1_at | -2,0 | 0,007 |
| similar to CG12822-PA, isoform A | LOC474773 | Cfa.11753.1.A1_at | -2,0 | 0,034 |
| RNA-binding region (RNP1, RRM) containing 3 | RNPC3 | CfaAffx.30574.1.S1_at | -2,0 | 0,030 |
| serologically defined colon cancer antigen 10 | SDCCAG10 | CfaAffx.11815.1.S1_at | -2,0 | 0,023 |
| tetratricopeptide repeat domain 8 | TTC8 | CfaAffx.26740.1.S1_s_at | -2,0 | 0,015 |
| retinoid X receptor, beta | RXRB | Cfa.9205.1.A1_at | -2,0 | 0,012 |
| MLX interacting protein-like | MLXIPL | Cfa.5237.1.A1_at | -2,0 | 0,030 |
| F-box and WD repeat domain containing 4 | FBXW4 | Cfa.6134.1.S1_at | -2,0 | 0,008 |
| similar to B0507.2 | LOC478996 | Cfa.2636.1.S1_at | -2,0 | 0,031 |
| JAZF zinc finger 1 | JAZF1 | Cfa.9214.1.A1_at | -2,0 | 0,017 |
| similar to T06D8.1a | LOC479089 | Cfa.15778.1.A1_s_at | -2,0 | 0,040 |
| collagen, type X, alpha 1 | COL10A1 | CfaAffx.7009.1.S1_at | -2,0 | 0,035 |
| nuclear transcription factor, X-box binding 1 | NFX1 | CfaAffx.3681.1.S1_at | -2,0 | 0,011 |
| cell division cycle 2-like 5 (cholinesterase-related cell division controller) | CDC2L5 | Cfa.834.1.A1_s_at | -2,0 | 0,011 |
| casein kinase 1, gamma 1 | CSNK1G1 | CfaAffx.26128.1.S1_at | -2,0 | 0,037 |
| splicing factor, arginine/serine-rich 5 | SFRS5 | Cfa.4157.1.S1_at | -2,0 | 0,009 |
|  |  |  | -1 |  |
