# Supplementary figures and images for: Canine Uterine Bacterial Infection Induces Upregulation of Proteolysis-Related Genes and Downregulation of Homeobox and Zinc Finger Factors
Source: PLoS One. 2009 Nov 26;4(11):e8039. doi: 10.1371/journal.pone.0008039 (PMC2777310; doi:10.1371/journal.pone.0008039)

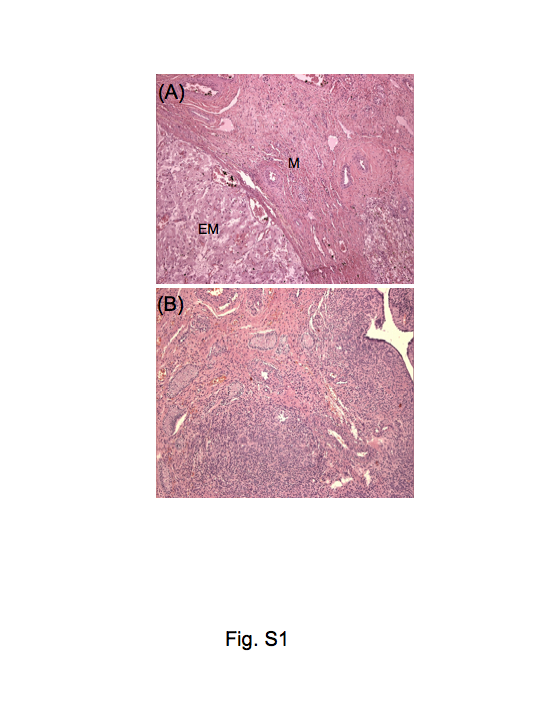

Supplement: Figure S1 — Histology of uteri from control (A) dogs and dogs diagnosed with uterine bacterial infection (pyometra; B). Note the extensive infiltration of mononuclear cells in infected animals, as well as the extensive loss of tissue organization. (0.34 MB TIF) [file pone.0008039.s005.tif]

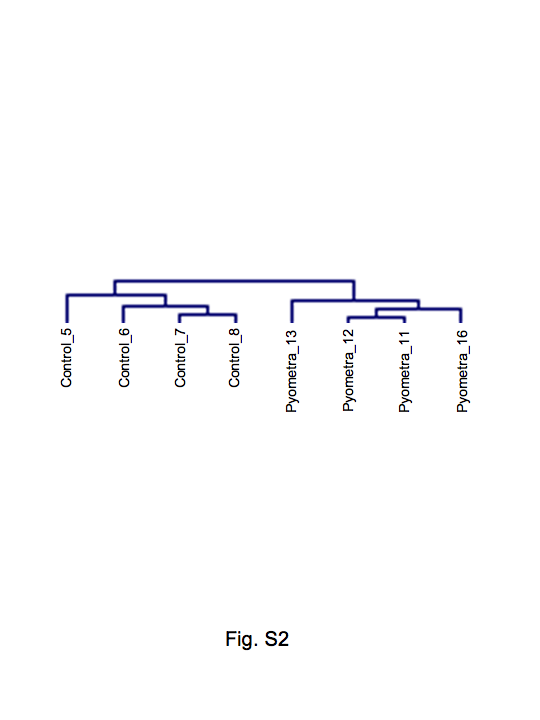

Supplement: Figure S2 — Unsupervised clustering of gene expression in uteri from control animals and animal diagnosed with uterine bacterial infection (pyometra), data derived from the Affymetrix Canine Genome 2.0 Array. (0.05 MB TIF) [file pone.0008039.s006.tif]
